# Application of artificial intelligence to decode the relationships between smell, olfactory receptors and small molecules

**DOI:** 10.1038/s41598-022-23176-y

**Published:** 2022-11-05

**Authors:** Rayane Achebouche, Anne Tromelin, Karine Audouze, Olivier Taboureau

**Affiliations:** 1 Université Paris Cité, CNRS, INSERM U1133, Unité de Biologie Fonctionnelle et Adaptative, 75013 Paris, France; 2 Centre Des Sciences du Goût Et de L’Alimentation, CNRS, INRAE, Institut Agro, Université Bourgogne Franche-Comté, 21000 Dijon, France; 3 Université Paris Cité, T3S, Inserm UMR S-1124, 75006 Paris, France

**Keywords:** Chemical biology, Computational biology and bioinformatics

## Abstract

Deciphering the relationship between molecules, olfactory receptors (ORs) and corresponding odors remains a challenging task. It requires a comprehensive identification of ORs responding to a given odorant. With the recent advances in artificial intelligence and the growing research in decoding the human olfactory perception from chemical features of odorant molecules, the applications of advanced machine learning have been revived. In this study, Convolutional Neural Network (CNN) and Graphical Convolutional Network (GCN) models have been developed on odorant molecules-odors and odorant molecules-olfactory receptors using a large set of 5955 molecules, 160 odors and 106 olfactory receptors. The performance of such models is promising with a Precision/Recall Area Under Curve of 0.66 for the odorant-odor and 0.91 for the odorant-olfactory receptor GCN models respectively. Furthermore, based on the correspondence of odors and ORs associated for a set of 389 compounds, an odor-olfactory receptor pairwise score was computed for each odor-OR combination allowing to suggest a combinatorial relationship between olfactory receptors and odors. Overall, this analysis demonstrate that artificial intelligence may pave the way in the identification of the smell perception and the full repertoire of receptors for a given odorant molecule.

## Introduction

Smell is a sense that allows the perception and discrimination of a large number of volatile environmental chemicals in the air by using the nose. It has been observed that smell is involved in the social behavior of many species but also in the location of food, ability to detect dangerous situations like fire, identification of predators, toxic compounds, mate choice and mother-infant recognition^1^. For humans, olfaction influences our well-being (looking for pleasantness) and play a major role in eating behavior with the perception of food quality and for social communication with the use of fragrance^2^. The smell impairment has a strong impact on the quality of life and it has been recently highlighted with COVID-19 causing the loss of smell of many individuals^3^.

The sense of smell is commonly associated with large and diverse families of odorant receptors that detect odor stimuli in the nose and transform them into patterns of neuronal activity that are recognized in the brain^4–7^.

In humans, it is estimated that millions and perhaps billion of odorant molecules are recognized by around 400 different human olfactory receptors (hORs)^8–11^. Odorants, commonly present in food, fragrance and cosmetic products, stimulate G-protein-coupled olfactory receptors (ORs) located in the olfactory sensory neurons of the nasal epithelium^12,13^. It has been reported than the olfactory system uses a combinatorial olfactory receptors code to encode an odor^14–16^. One odorant can interact with several different ORs and one OR can be activated by a large panel of molecules. Although recent optimizations in functional expression of ORs for the screening of odorant compound libraries have been made, investigating all combinations is still expensive, time consuming and remains therefore a tremendous challenge^17^.

It is important to notice that the semantic is a source of complexity for the verbal description of odors^18,19^. Indeed, the description of the odor of a molecule involves several odor attributes, or odor notes, which are “odor objects” i.e., the odors perceived in our environment^20–22^. Yet, these odors result from the perception of numerous odorant molecules, which increase the difficulty to have reliable odors descriptions.

Despite some experimental studies have identified odorant-OR interactions in some organisms (mainly in mammals and insects)^23–26^, the link between activation of ORs and odor perception remains limited^9,27–31^. Considering that the perception depends on chemistry, several studies have attempted to connect odorant physicochemical properties to the olfactory perceptions^32–38^. Crowd-sourced DREAM Olfaction Prediction Challenge was organized in the aim to predict human olfactory perception for 19 semantic descriptors for odors as well as intensity and pleasantness based on chemical features and machine learning models^39^. Such analysis can then be used to identify new structural motifs for ligands during large virtual screening campaigns^40,41^. Recently, artificial intelligence technology using deep neural networks (DNN)^42^, graph neural networks (GNN)^43^ or convolutional neural networks (CNN)^44,45^ have been performed to underlie the relationship between the structure of chemicals and odors. They reported that such machine learning approaches outperformed classical methods applied to chemical-odor relationships.

Based on these observations, we decided to go one step further and to analyze the relationships of chemical-odors and chemical-olfactory receptors based on the chemical structure of odorant using deep learning approaches such as graph neural networks (GNN), and convolutional neural networks (CNN). The relationship between chemicals—olfactory receptors and odor perception is of high interest in the determination of (i) chemical properties—odor relationships, (ii) chemical properties—olfactory receptors relationships and (iii) olfactory receptors—odor relationships. Furthermore, the global chemicals-olfactory receptors-odors relationship has been investigated using a confidence score proposing a combination of receptors that can play a role in the perception of odors.

## Materials and methods

### Datasets

This study is based on the integration of two different data sets (i) data for chemical-odor relationships and (ii) data for chemical-olfactory receptor relationships.

#### Chemical-Odor

We extracted chemical-odors from two separate sources: The Good Scents Company (TGSC) Database^46^ (as of January 2021), and Leffingwell Database^47^. Both databases contained information linking the compound and its chemical structure to the odor description as several odor notes. From the TGSC database, we got 27,779 chemicals of which 5659 are related to one or several odor notes. From the Leffingwell database, we got 6054 compounds that are related to one or several odors notes. We merged the outcomes from both databases, eliminating duplicated information. Compounds occurring with the same structure (based on Inchi Key encoding^48^) but with different names (synonyms) were removed. Odor notes from Leffingwell database was matched with TGSC as reference. To limit the complexity of the models and avoid mis-classification due to poor representation of an odor note, odor notes with less than 20 chemicals were not considered in this analysis. After all these steps, we obtained a dataset made up of 5955 compounds and 160 odors. Each compound is related from 1 to at the maximum 10 odor notes using the order proposed by TGSC.

#### Compound-olfactory receptor

Compounds tested experimentally on olfactory receptors were gathered from different data sources. It included information from OdorDB^49^, ODORactor^50^, OlfactionDB^51^ and from the literature. To the purpose of the study we considered, first, human receptors in the construction of learning models. We collected 74 human olfactory receptors for 365 compounds. In a second step, human receptors that are orthologs to rodent olfactory receptors, and on which bioactivity has been measured, were also included in the learning model development. With the aggregation of this data, we reached a dataset of 445 different compounds tested on 106 different olfactory receptors.

The datasets generated and analysed during the current study are available in the Table S1 in supplementary.

### Methods

#### Global overview of the odorant molecules

To visualize the distribution of the molecules according to their odors and their activity on olfactory receptors, the structure of each molecule was encoded into 1024 ECFP (Extended Connectivity Fingerprint) fingerprints^52^. Then, the matrix of fingerprint was projected into a 2D map using a reduction technique, UMAP (the Uniform Manifold Approximation and Projection), that was applied recently with smell compounds^53^. Such projection allows to look over the distribution of the molecules in a 2D space and to map corresponding odors and olfactory receptors associated to each molecule.

#### Machine learning models

Different machine learning models have been generated in order to assess their performance in the prediction of compound-odor and compound-olfactory receptor relationships. Since one compound can be related to one or more odors, it raised a multi-label classification problem. Consequently, we developed 3 types of models adapted for multiclass: (i) a Random Forest model, based on RDKit descriptors^54^ and ECFP (Extended Connectivity Fingerprint) fingerprint, (ii) a Convolutional Neural Network (CNN) based on ECFP and (iii) a Graph-based Neural Network (GNN). Random Forest were built using scikit-learn python package^55^, GNN with DeepChem^56^ and CNN with Tensorflow^57^. The evaluation metric used was the Area Under ROC Curve (AUROC) and the Precision/Recall Area Under Curve (PRC-AUC). For CNN and GNN, an internal validation of the models was carried out using a fivefold Cross Validation for each of them.

#### Random forest (RF) models

For the RF models, in a first strategy, the molecule’s structure was encoded in 154 2D descriptors (using RDKit) and the odor notes labels binarized in 0 or 1. Then, a RF model was built using 500 mtrees and 15 ntry. We optimized the hyperparameters in order to minimize the Out Of Bag Score (OOB Score) and maximize the AUROC score (Table 1).

**Table 1:** Parameters considered in the compound-odor Random Forest models.

n_estimators	criterion	max_depth	min_samples_split	min_samples_leaf	max_features
500	‘gini’	75	2	1	15

In a second strategy, the chemical structure was encoded into ECFP fingerprints. This type of fingerprint was chosen because it is a method of vectorial representation of molecules quite similar to the one used for the Graph-based Model (described below). Thus, a 1024-bit ECFP fingerprint was generated for each molecule and then a RF model was performed using the same parameters.

A similar RF protocol was also applied with olfactory receptors using the same parameters.

#### Convolutional neural network (CNN) model

A convolutional neural network (CNN) model was developed based on ECFP fingerprints encoding. At the difference of RF, CNN is a method based on neuron convolutions. In our CNN model, the architecture of the network is organized as follows: the concatenation of the message is done by 2 layers of dimensions [32, 32] with a rectifier linear unit activation function ‘RELU’, a batch normalization that standardize input data in order to reduce the number of epochs for training network, and finally a maxpooling parameter that reduce spatial size by some operations, preventing overfitting and reducing computational cost. The fully connected neural net consists in layers of a size 128 dots (Dense layer). The readout is done using a softmax function with 160 tasks for odors (and 106 tasks for olfactory receptors) and a Categorical Cross Entropy loss function. The model has been trained on 300 epochs and a 5 folds cross validation was performed (60 epochs for each fold). More information about the CNN implementation can be obtained here^58^.

#### Graph-based neural network (GNN) model

We decided to develop a graph-based Neural Network (GNN) model because it is close to the architecture of the model based on molecular graphs. By considering chemical bonds as edges and atoms as nodes, molecules can be represented as graphs. This type of representation can then be used to develop graph-based model. In our study we considered the implementation of a Graph Convolutional Network (GCN)^59^. GCN consists of message passing layers, followed by a reduce-sum operation to obtain at the end, a fully connected layer. In a first step, each molecule is featured into a set of fixed-length vectors where each vector is calculated for each atom. Once the molecule has been featured, a series of operations consisting of concatenating the message takes place. This is the convolutional part of the model. Then, each molecular graph is reduced to a vector that will yield a fully connected neural network for final prediction. The architecture of the network is as follows: the concatenation of the message is done by 2 layers of dimensions [64, 64] with *rectifier linear unit activation* function *‘RELU’*, a *batch normalization* that standardize input data in order to reduce the number of epochs for training network, a *dropout* that omit some units to prevent from overfitting and finally a *maxpooling* that reduce spatial size, prevent overfitting and reduces computational cost. The fully connected neural net consists of a layer of a size 128 (Dense layer) with *RELU* activation and *batch normalization*. The readout is done using a *softmax function* with 160 tasks for odors (and 106 tasks for olfactory receptors) and a *Softmax Cross Entropy* loss function.

The model has been trained on 300 epochs and a 5 folds cross validation was performed (60 epochs for each fold).

In addition to this model, a second GCN was created by grouping the odors by categories in order to predict the corresponding categories rather than each odor note individually. Thus, the parameters used for this model are the same as the GCN presented above. The odor notes have been grouped according to the correspondences shown in the Table 2.

**Table 2:** Grouping of odor notes in categories having a similar perceptual space.

Natural	"green", "herbal", "earthy", "natural", "leafy", "oakmoss", "weedy", "grassy", "hay", "ozone", "malty"
Woody	"cedar", "woody", "camphoreous", "pine", "fir needle", "sandalwood", "terpenic"
Fruity	"fruity", "pineapple", "apple", "currant", "peach", "berry", "grapefruit", "grape", "apricot", "banana", "cherry", "melon", "pear", "plum", "raspberry", "ripe", "strawberry", "coconut", "citrus", "lemon", "orange", "bergamot", "mandarin", "nutty", "hazelnut"
Spicy	"peperry", "spicy", "mustard"
Fresh	"fresh", "minty", "cooling", "mentholic"
Dairy	"milky", "dairy", "cheesy", "creamy", "buttery"
Floral	"floral", "rose'7'orangeflower", "chamomile", "lavender", "geranium", "hawthorn", "jasmin", "jasmine", "hyancinth", "lilac", "lily", "lily of the valley", "magnolia", "muguet", "narcissus", "orchid", "savory", "violet", "vetiver", "ylang", 'mimosa', "patchouli", "hyacinth", "tea", "cinnamyl", "orris", "tropical"
Alcohol	"alcoholic", "cognac", "brandy", "winey"
Spice	"cum in", "cinnamon", "rum my", "clove", "licorice", "tonka", "anisic"
Fat	"oily", "fatty", "waxy"
Sweet	"sweet", "caramellic", "honey", "vanilla", "almond", "cocoa", "chocolate", "popcorn"
Vegetable	"peppery", "radish", "cucumber", "cabbage", "tomato", "celery", "potato", "vegetable", "corn", "mushroom"
Marine	"marine", "fishy"
Meaty	"meaty", "beefy", "roasted", "smoky"
Animal	"animal", "musk", "tallow", "amber"
Clean	"clean", "bready", "soapy"
Sharp	"pungent", "sharp", "alliaceous", "garlic", "onion", "leathery", "bitter", "sour"
Cooked	"cooked", "toasted"
Odorless	« odorless »
Unpleasant	"musty", "sulfurous", "ammoniacal", "fermented"
Balsamic	« balsamic »
Large	"ethereal", "aromatic", "phenolic", "coumarinic", "aldehydic", "ketonic", "lactonic"
Other	burnt", "coffee", "cortex", "dry", "dusty", " medicinal", "metallic", "gassy ", "juicy", "solvent", "sweaty", "tobacco", "warm", "plastic", " powdery"

#### Odor-receptor model

From the two datasets, 383 compounds targeting olfactory receptors and also related to odor notes were identified. It means that for each molecule, odor notes and olfactory receptors correspondence can be highlighted. Given the imbalance in the two data sets and the imbalance in the binary classes (much more negative than positive outcomes), an odor-olfactory receptor pairwise (OORP) score was computed between the odor and receptor information based of the common active compounds using the equation below:

$${\text{OORP}}_{{{\text{OiORyP}}}} = \left( {{\text{C}}_{{{\text{OiORy}}}} /{\text{Ctot}}_{{{\text{Oi}}}} + {\text{C}}_{{{\text{OiORy}}}} /{\text{Ctot}}_{{{\text{ORy}}}} } \right)/{2}$$

With C_OiORy_ being the number of compounds common between an odor (O_i_) and an olfactory receptor (OR_y_), Ctot_Oi_ being the total number of compounds associated to the odor notes (O_i_), and Ctot_ORy_ the total number of compounds associated to the olfactory receptor (OR_y_).

The odor notes-olfactory receptor pairwise score is between 0 and 1. The closer to 1 is the score, the more significant is the relation between an olfactory receptor and an odor note.

## Results

### Global analysis of the data collected

**Figure 1:**
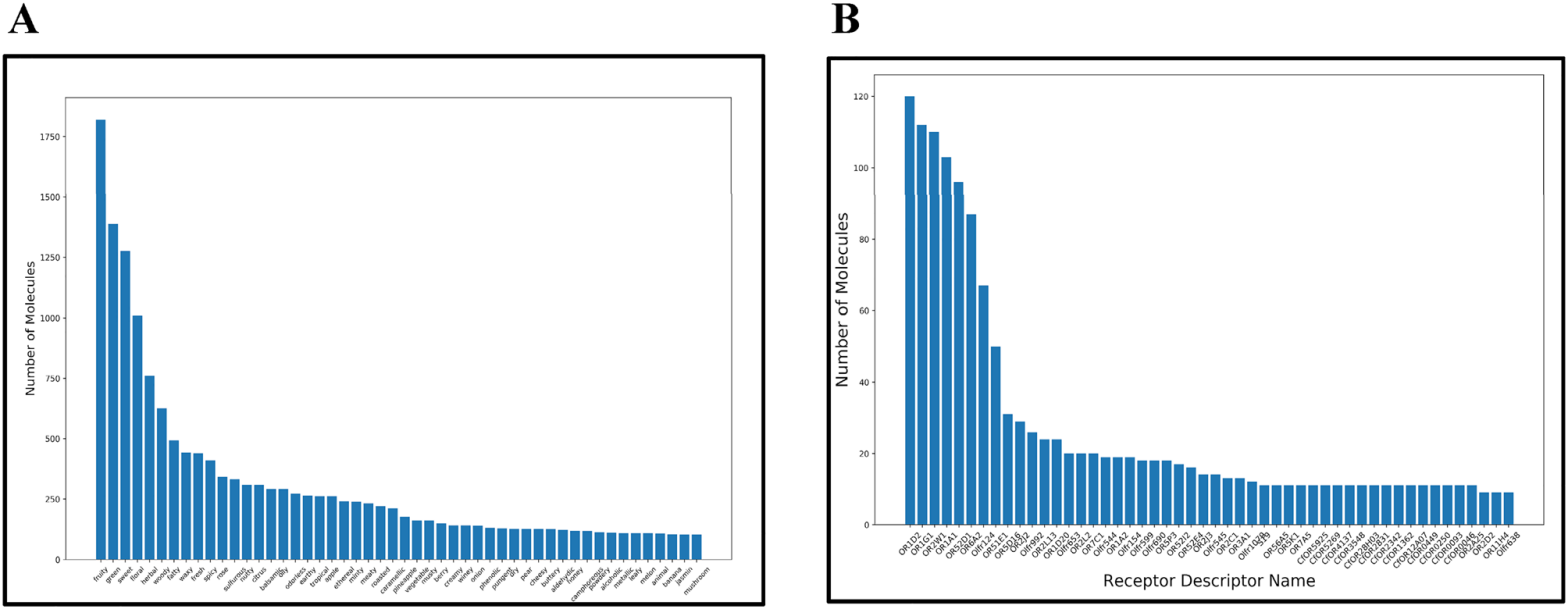
(**A**) Occurrence of compounds related to odors. (**B**) Occurrence of compounds related to olfactory receptors.

The data collected on chemicals, olfactory receptors and odor notes are very heterogeneous, with many molecules for some odor notes/receptors and very few for others. Fruity is the odor associated with the highest number of molecules (> 1750) (Fig. 1A). More than 1000 molecules are sweet, green and floral. At the difference, less than 200 molecules are associated with mushroom, jasmin or banana. We have to notice that a molecule is usually associated with several odor notes. On average, a molecule has 3, 4 odors which is in agreement with previous studies^37,38^. Some odor notes could be closely related to each other and an odor note could be a more specific term to a general category of odor note. Like for example banana, melon, pear or apple are specific odor notes but also belong to a more general fruity odor.

Similarly, looking on the relation between compounds and olfactory receptors, it is observed that OR1D2, OR1G1, OR2W1, OR1A1, OR52D1, OR6A2 and olfr124 (ortholog to OR2B4 in human) are receptors with more than 50 molecules interacting to them (Fig. 1B). On average a molecule interacts with 3,46 olfactory receptors.

**Figure 2:**
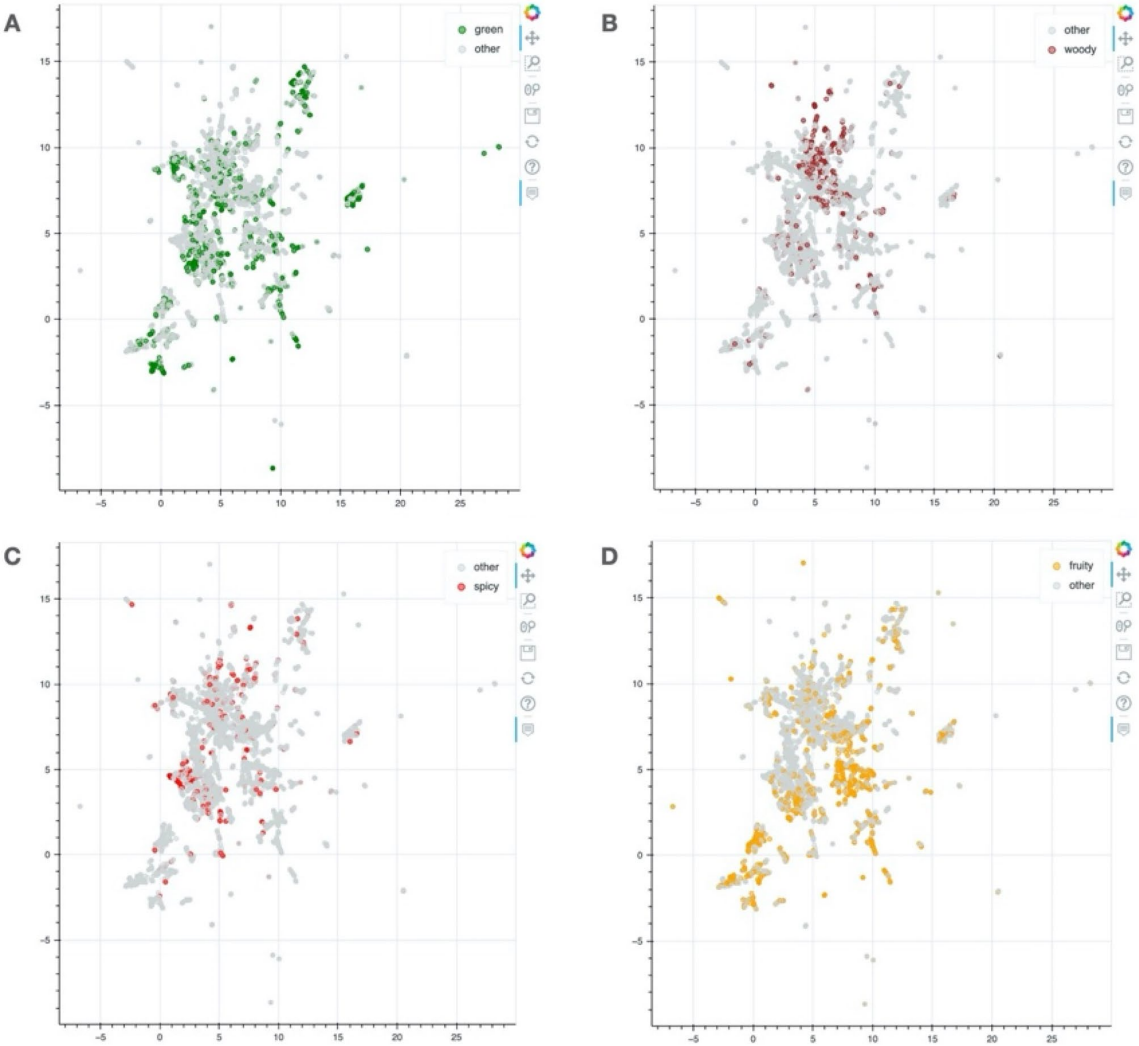
UMAP representation of compounds distribution in a 2D map projection. (**A**) Compounds with green odor note, (**B**) Compounds with woody odor note, (**C**) Compounds with spicy odor note, (**D**) Compounds with fruity odor note. The UMAP representations were developed with the python package bokeh (v.2.4.3): https://docs.bokeh.org/e^60^.

Using a UMAP visualization technique, the relation between chemical structure, odor notes and olfactory receptors can be depicted in an interactive 2D map. It is a way to represent the distribution of molecules in a 2D space. For example, comparing compounds having fruity, spicy, woody and green odor notes (Fig. 2), some compounds are more grouped in some area of the map and others compounds are more spread all over the chemical space. It means that there are some specific structural features for some compounds associated to a specific odor note compared to others odor notes for which it is more general.

**Figure 3:**
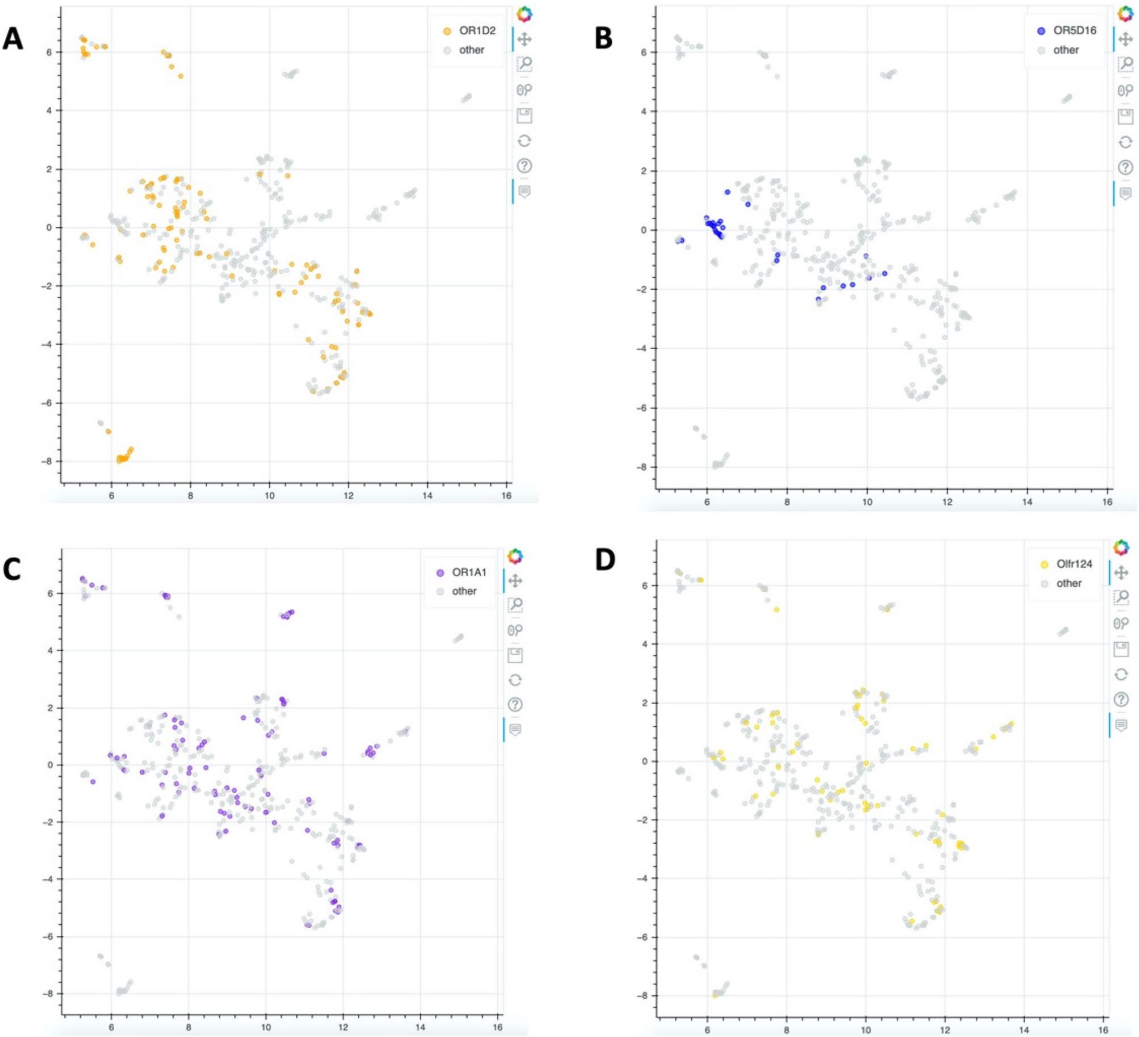
UMAP representation of compounds distribution in a 2D map projection. (**A**) Compounds active on OR1D2, (**B**) Compounds active on OR5D16, (**C**) Compounds active on OR1A1, (**D**) Compounds active on OR2D4. The UMAP representations were developed with the python package bokeh (v.2.4.3): https://docs.bokeh.org/e^60^.

A similar observation can be concluded for some olfactory receptors, notably the OR1D2, OR5D16 for which some bioactive compounds on theses receptors are grouped in some area of the map while others ORs (OR1A1, OR2B4) are more spread over the chemical space (Fig. 3).

**Figure 4:**
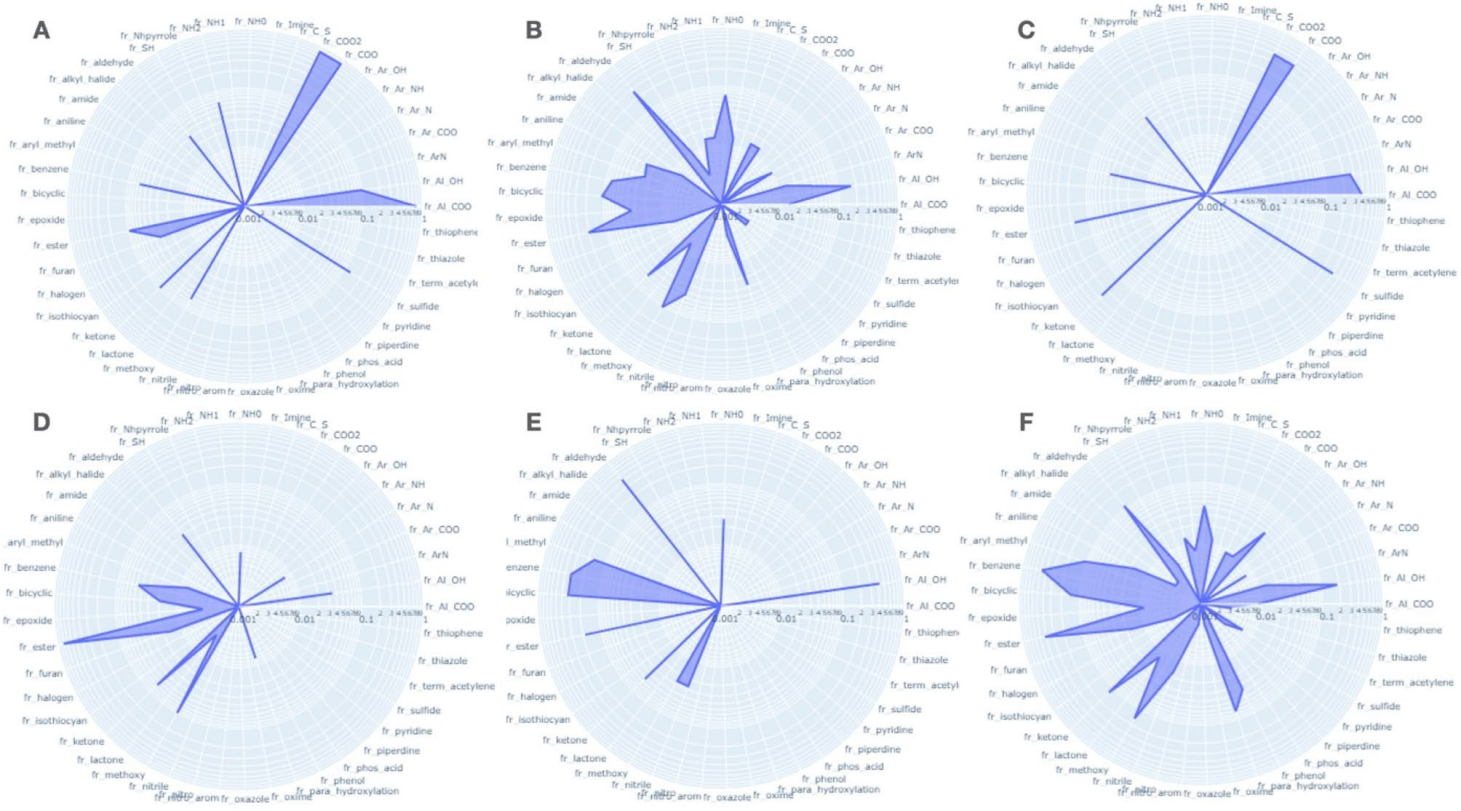
Radar plot based on the frequency of physicochemical properties observed on the set compounds associated to an odor note. (**A**) Acidic, (**B**) Citrus, (**C**) Cheese, (**D**) Apple, (**E**) Muguet, (**F**) Floral. The radarplots were developed with the python package plotly (v.5.3.1): https://plotly.com/^61^.

To look over the frequency of chemical groups related to odors and receptors, radar plots have been developed with 62 molecular substructure and group of atoms. Based on these plots, an ensemble of structural features that occur more frequently with some odors but also with some olfactory receptors can be observed (Figs. S1 & S2 in supplementary). For example, a majority of compounds associated to the odor note ‘acidic’ possess a COO group. However, compounds associated to ‘citrus’ odor note are represented by a sparser ensemble of group of atoms (OH, aldehyde, ester, methoxy, NH…). Interestingly, the ‘cheese’ odor note is also highly associated to the presence of a COO group in a compound. Globally, specific odor notes that are associated to a fruit (apple, apricot, banana), a vegetable (celery, cucumber) or a flower (rose, muguet, narcissus) are related to few groups of atoms while general class of odors i.e., fruity, floral, sweet, phenolic encompass larger groups of compounds with a higher diversity in physicochemical properties (Fig. 4).

**Figure 5:**
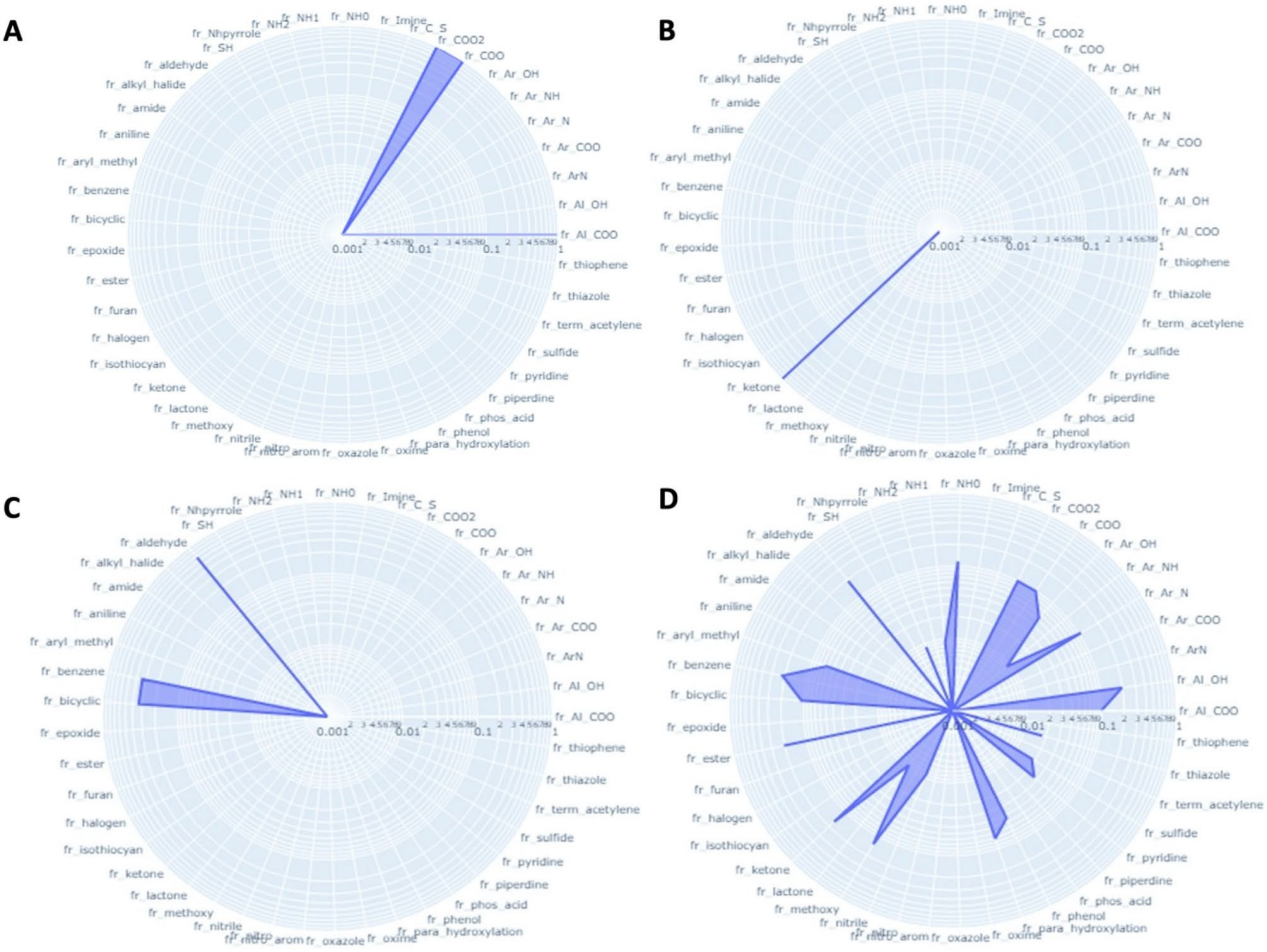
Radar plot based on the frequency of physicochemical properties observed on the set compounds associated to an olfactory receptor. (**A**) OR52E1, (**B**) OR4D6, (**C**) OR1D3, (**D**) OR1G1. The radarplots were developed with the python package plotly (v.5.3.1): https://plotly.com/^61^.

With olfactory receptors, some specific structural features are also more frequently observed with some ORs while other Ors are less specific and can be impacted by different groups of molecules. For example, a majority of molecules associated to OR52E1 possess a carboxylic group, OR4D6 ligands have a ketone, OR1D3 ligands have a benzene, a bicyclic and an aldehyde group. Similar to odors, it is observed that Ors with a large set of compounds (i.e., OR1G1, OR2W1, OR1A1, OR52D1, OR6A2) are also associated to compounds with diverse groups of atoms (Fig. 5). So, it could be assumed that some Ors are more selective to some ligands with specific features than others Ors that are more general^62^.

### Results on ligand-odor notes model

Once, the global analysis of these data was realized, machine learning models were developed to predict in one hand the odor notes and in the other hand the olfactory receptors, associated to a molecule. About the ligand-odor note models, 3 types of models were built i.e., Random Forest, Convolutional Neural Network (CNN) and Graph Convolutional Network (GCN). Based on the AUROC and the PRC-AUC estimation, the GCN showed the best performance of prediction, with an AUROC = 0.96 and a PRC-AUC = 0.49 (Table 3). Random Forest models have inferior performance with both Morgan Fingerprints and RDKit descriptors. CNN model based on Morgan fingerprints was the worst with an AUC = 0.53 and a PRC-AUC = 0.04. So, models based on neural network and graph-type information seems to have better performance. To evaluate the robustness of the models, A fivefold cross validation was performed. Although the AUROC is still high, the PRC-AUC went down to 0.24 respectively. The unbalanced data set might explain this reduction of PRC-AUC performance.

**Table 3:** Performance of the 4 models applied on the compound-odor note dataset. The results in bold are the performance on the full dataset. The value in brackets depicts the results of the fivefold cross validation model.

	Compound—Odor note	
	AUROC	PRC-AUC
GCN	**0.964** [0.82]	**0.497** [0.24]
RF-Morgan Fp	**0.631**	**0.222**
RF-RDKit Features	**0.644**	**0.241**
CNN-Morgan Fp	**0.532**	**0.042**

In more details, the performance for each odor note, odor notes with high PRC-AUC such as ‘malty’ (0.99), ‘odorless’ (0.89), ‘maple’ (0.85), ‘sandalwood’ (0.84), ‘alcoholic’ (0.83), ‘musk’ (0.83), ‘ambergris’ (0.81) and odors with low performance i.e., ‘tea’ (0.13), ripe (0.18), ‘chocolate’ (0.21), ‘metallic’ (0.21), ‘aromatic’ (0.22) can be identified (supplementary Table S2).

**Figure 6:**
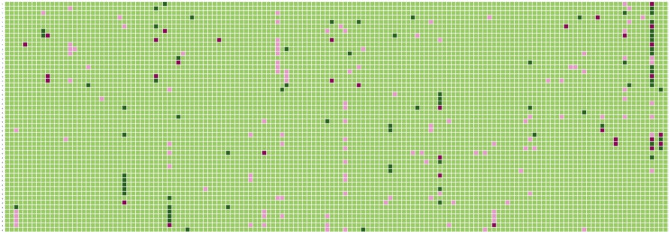
Heatmap representation of the performance with the GCN model for a subset of the compound-odor note associations. The odor notes are represented on the X-axis and the compounds on the Y axis. A compound associated to an odor note and correctly classified by the GCN model is colored with a dark green cell. A compound not associated to an odor note and correctly predicted has a light green color. A compound linked to an odor note and wrongly predicted by the model is represented with a pink color and a compound not linked to an odor note and predicted to be associated to this odor note is shown with a red color. The heatmap was developed with the python package seaborn (v.0.11.2): https://seaborn.pydata.org/^63,64^.

The prediction of odor notes associated for each molecule by the GCN model can also be depicted in a heatmap (supplementary Fig. S3). A representation for a subset of compounds is depicted in Fig. 6.

Based on this heatmap, we can observe that many compounds are predicted to ‘sweet’ and the ‘fruity’ odors with a mixed of good and bad prediction. Floral, fresh and herbal odors notes are also general classes of odors with many mis-classified compounds (pink color). For some compounds, the classification is excellent with no misclassification. This is the case for example, for 3-phenyl propyl alcohol which is correctly predicted to the odor note balsamic and sweet; butyl acetate which is related to banana, ethereal, fruity and solvent; (E)-isoeugenyl acetate which is correctly predicted to spicy and clove and (Z)-7-decenal which is predicted to citrus, aldehydic and cucumber among others. However, many compounds have a combination of good and bad predictions. At the opposite, some compounds are wrongly predicted and do not capture the odor note on which it has been associated with. This is the case for “benzyl acetone” which is not predicted by the model to be associated to balsamic and floral but for which the model is predicted the odor of almond and sweet. The model is not able to annotate the animal odor note for skatole compound, neither the fruity, fatty, cheesy, herbal coconut odor note for the 2-nonanone compound.

As some odors might be relatively close in perception (for example citrus vs lemon, cheese vs cheesy), a second GCN model was developed by grouping the 160 odors in 23 categories. The results in Table 4 depicts a good AUROC performance (0.92). Interestingly, the PRC-AUC performance is higher with a score of 0.67 (0.40 in cross validation). Therefore, the GCN model seems more robust and suitable with a reduced number of odors.

**Table 4:** Performance of the GCN model applied on the compound-odor dataset grouped on 23 categories. The results in bold are the performance on the full dataset. The value in brackets depicts the results of the fivefold cross validation model.

	Compound—Odor GROUP	
	AUROC	PRC-AUC
**GCN**	**0.92** [0.80]	**0.67** [0.40]

### Results on ligands-receptors model

Similarly, to the previous models developed on compound-odor relationships, RF, CNN and GCN models were developed on ligand-receptor information. At the difference of the compound-odors relationships models, the ligand-receptor dataset is smaller containing 365 odorants with known bioactivity on 74 human olfactory receptors. Developing a GCN model on this dataset we obtained a AUROC = 0.98 (0.67 in cross validation) and a PRC-AUC = 0.71 (0.22 in cross validation). The large drop observed for the PRC-AUC in cross validation indicate that the model is not too stable and might be due to a limited size of the data set. Therefore, we decided to enrich our dataset with the integration of chemicals having a bioactivity on rodent olfactory receptors orthologs to human receptors, assuming that they share a similar mechanism of action. With this step, predictive models were developed based on 445 compounds with known bioactivity on 106 olfactory receptors. The performances of the models are presented in Table 5. Again, the GCN model have higher AUROC (0.99) and PRC-AUC (0.91) than the other machine learning models. The GCN model conserved a good AUROC score in cross validation (0.71) and with a better PRC-AUC score (0.4). These results suggest that the model’s performance is dependent on the data inclusion. The scattering of the compound—olfactory receptors information might be a cause of the fall of the PRC-AUC when using a subset of the compound-OR data set.

**Table 5:** Performance of the 4 models applied on the compound-olfactory receptors dataset. The results in bold are the performance on the full dataset. The value in brackets depicts the results of the fivefold cross validation model.

	Compound—Receptor (human + orthologs)	
	AUROC	PRC-AUC
GCN	**0.99** [0.71]	**0.91** [0.40]
RF-Morgan Fp	**0.612**	**0.212**
RF-RDKit Features	**0.586**	**0.167**
CNN-ECFP4	**0.536**	-

Looking on the GCN model performance for each OR (Table S3 in supplementary), we observe that many ORs have the maximum AUROC and PRC-AUC score (OR5A2, OR4D6) while others ORs obtained low PRC-AUC (OR56A1, OR52M1, OR56A4). The fact that some ORs have few compounds associated may facilitate the good performance for these odors.

**Figure 7:**
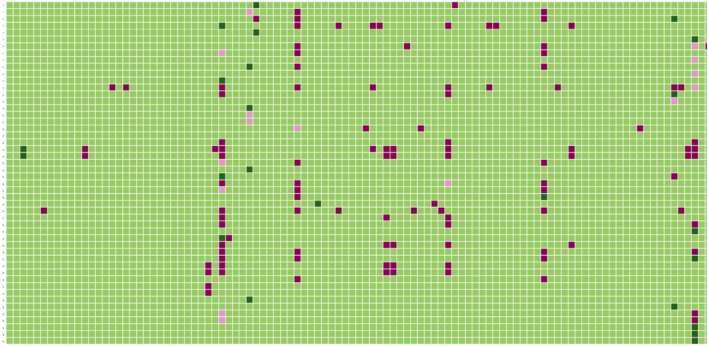
Heatmap representation of the performance prediction with the GCN model for a subset of the compound-olfactory receptors (ORs) associations. The ORs are represented on the X-axis and the compounds on the Y axis. A compound associated to an OR and correctly classified by the GCN model is colored with a dark green cell. A compound not associated to an OR and correctly predicted has a light green color. A compound linked to an OR and wrongly predicted by the model is represented in a pink color and a compound not linked to an OR and predicted to be associated to this odor is shown with a red color. The heatmap was developed with the python package seaborn (v.0.11.2): https://seaborn.pydata.org/^63,64^.

On the heatmap (Fig. S4 in supplementary), we can observe that some ligands are correctly predicted i.e., coffee difuran predicted active on OR1A1, butyrophenone on OR6A2, 4 phenyl-1 butanol on OR1G1, (E)-cinnamyl nitrile on OR1D2 and 4-tert-butyl cyclohexanone active on the human ortholog OR5D16 (olfr73 in mouse). A large set of compounds are wrongly predicted on OR5A1, OR52D1, OR56A2. In fact, these receptors are annotated to molecules with diverse physicochemical features, generating some difficulty to the models to discriminate between true positives and false positives. An example of the heatmap representation is depicted in Fig. 7.

### Results on receptors-odor notes relationship.

**Figure 8:**
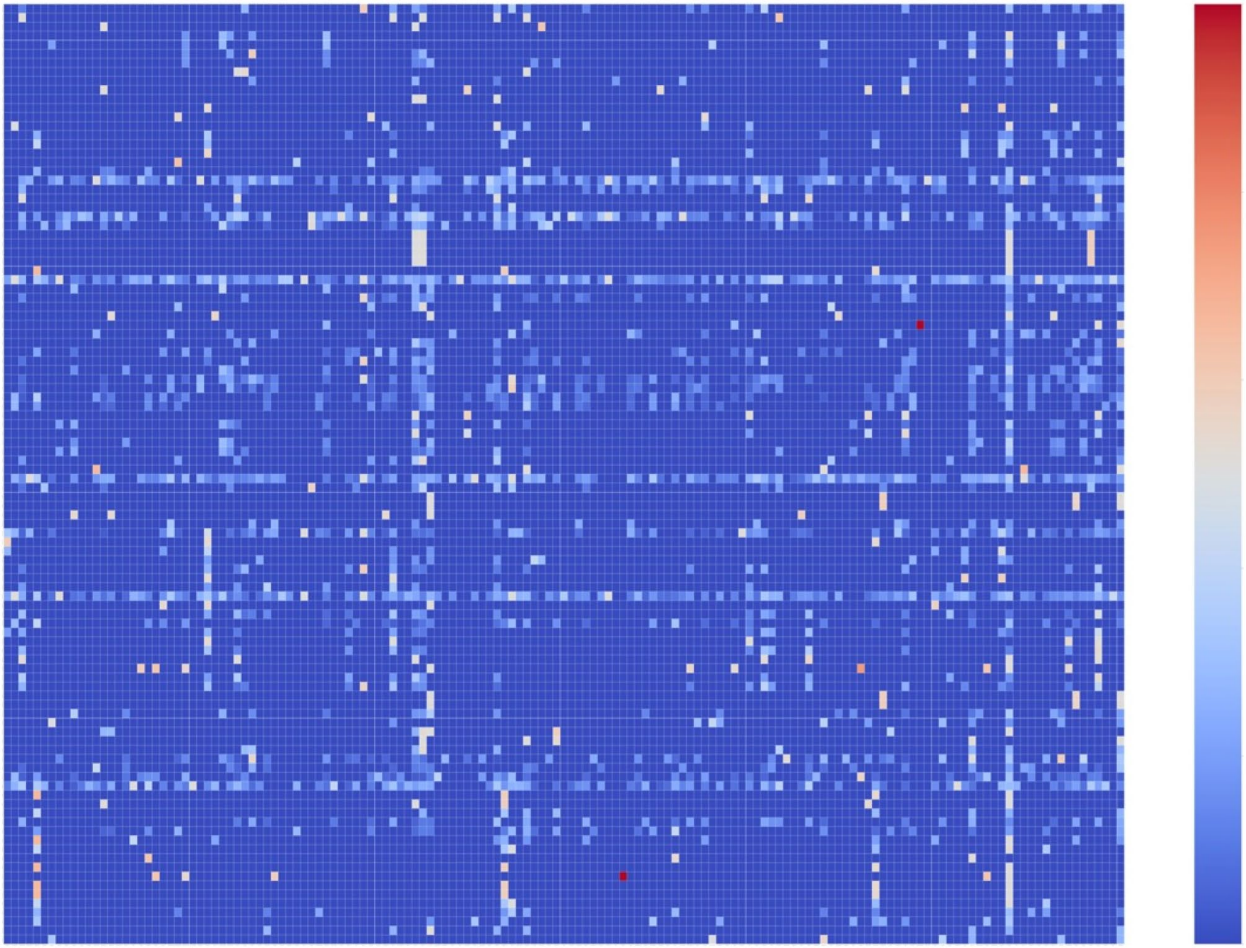
Heatmap representation of the odor note-olfactory receptor pairwise score for a set of 383 compounds targeting olfactory receptors and related to odor notes. The ORs are represented on the X-axis and the odor notes on the Y-axis. A red dot represents a high odor note-olfactory receptor pairwise score and a dark blue dot, no odor note-olfactory receptor relationship. The heatmap was developed with the python package seaborn (v.0.11.2): https://seaborn.pydata.org/^63–65^.

As 357 compounds targeting human olfactory receptors and related to odor notes were identified in our data sets, an odor-olfactory receptor pairwise score between each odor and each receptor i.e., the possible relation between odor notes and receptors, was computed (supplementary Table S4) and represented within a heatmap (Fig. 8). Globally, based on 151 odor notes and 104 ORs, such heatmap allows to suggest relation between olfactory receptors and odor notes due to the number of shared compounds. Some ORs seem more related to some odor notes than others. For example, the corn odor note is uniquely associate to OR1G1. The patchouli odor note is associated to OR5D16 and the cumin odor note is associated to OR1D2. The savory odor note is more associated with the OR1A1 receptor (OORP = 0.51) while waxy and woody odor notes are strongly associated with the OR2AT4 receptor (OORP = 0.51 and OORP = 0.52 respectively)*.* Interestingly such matrix gives a score for each OR on each odor note. It means that a set of ORs can be suggested to a set of odor notes. For example, OR1G1 and OR1D2 are associate to more than 70 odor notes reflecting no high specificity of these ORs to odors. At the difference, OR10A6 is linked to balsamic, floral and hyacinth. OR1E3 is linked to almond, hawthorn, pungent and sweet and OR8D1 is strongly associated to burnt, carmellic, coffee, maple, sugar and sweet. From the literature, some of these potential associations have been confirmed. Triller et al. 2008 mentioned that OR1D2 is highly related to muguet^65^. Veithen et al. show that OR1D2 might be also related to floral, fruity, citrus^66^. In our study, in addition to these odor notes, high relation with lactonic, rose and peach are also observed. A patent suggested that the olfactory receptors R52L1, OR52E8, OR52B2, OR5112, OR52E1, OR52A5, OR56A5 are involved in the perception of human sweat^67^. In addition, it is claimed that chemicals with a carboxylic acids group could be the relation between these ORs and the sweat odor. In our analysis, the olfactory receptors OR117P and OR52B2 contribute in majority with the sweaty odor note.

### Comparison of models’ performance

To assess the performance of these models, we compared the results of our chemical-odor models to the DREAM Olfaction Prediction Challenge^39^, and our chemical-odor and OR-odor models to the recent ones reported by Kowalewski et al^40^.

About the chemical-odor model, we used the same 69 test chemicals from the DREAM Olfaction Prediction Challenge^39^ to evaluate our model performance. For the odor prediction, we obtained an average balanced accuracy (BA) of 0.71 using as positive the compounds up to the top 10% perception for an odor^39^ (supplementary Table S5). Compared to the recent AUC of 0.78 obtained by Kowalewski et al. our model has a little lower performance. Looking at the 19 perceptions from DREAM, our models have a relatively good BA (> 0.7) for ‘bakery’, ‘fish’, ‘garlic’, ‘acid’, ‘sweaty’, ‘amonia/urine’ ‘wood’ and ‘grass’. For the other perceptions, the BA is weaker. It can be explained by the fact that matching the 160 odors used in our study to the 19 perceptual odors considered in Keller et al. publication^39^ might increase the number of false positive rate. For example, the odors “cold”, “decayed” and “warm” are not specifically annotated in our odors collection and grouping some of the odors in our dataset might bring some noise in this comparison exercise.

About the ligand-OR model, Kowalewski et al. used the same external set of 69 chemicals to predict associated olfactory receptors to them. Having only the chemical-ORs prediction from their study (and not the experimental value) we could only compared their prediction to our model’s result for 23 olfactory receptors (supplementary Table S6). Interestingly, half of their prediction was retrieved in our models. In general, there models predicted around 3 times more chemical-OR relationship compared to our model (354 versus 120 chemical-OR predictions) for this set of olfactory receptors.

Finally, about the OR-odor, in the Kowalewski et al. publication, 34 human ORs-perception were predicted. Interestingly, compared to our results, we can observe similar OR-odor note relationships like for example OR52D1 with ‘animal’, ‘sweaty’, ‘rose’ and ‘violet’, OR2B11 with ‘coffee’ and OR2W1 with ‘spicy’, ‘clove’, ‘caramel’ and ‘cheesy’ among others. At the difference for others ORs, we obtained different relationships. For example, our study suggests that OR1A2 contribute in priority with the odors ‘aldehydic’, fatty’, ‘grassy’, ‘hay’, ‘ozone’ whereas in their studied, important relationships between OR1A2 and ‘warm’ and ‘sweet’ were reported. We suggest also that OR1D2, OR1G1, OR52D1 and OR6A2 could contribute to the odor note ‘fishy’ whereas there heatmap showed a higher contribution of OR2T34 and OR51E1.

Overall, the fact that different data sets of ligand-odor notes and ligand-olfactory receptors are used in both studies has probably an impact on the results. Further experiments should help in the precision of these predictive models.

## Discussion–conclusion

Using, a large data set of 5955 compounds, 160 odors and 106 olfactory receptors, machine learning models based on artificial intelligence i.e., Random Forest, CNN and GCN approaches were developed. Such models can then be used to predict the odor note(s) and olfactory receptor(s) associated for a new compound using the chemical structure of it. In addition, based the correspondence of odor notes and ORs associated for a set of 389 compounds, a score was computed for each odor note-OR combination allowing to decipher the combinatorial relationship between olfactory receptors and odor notes.

Although the results are promising, there are still some limitations and the models will need to be optimized in the aim to increase their performance.

First, the perception of an odor is highly dependent of an individual and odors annotation to a compound are suggestive, depending of ethnicity, alimentary behavior, age^68–72^. Indeed, the definition of some odor notes might be fuzzy (cheese vs cheesy). Recently, 540 individuals were asked to rate the intensity and pleasantness of 9 musk compounds and their ORs were sequenced in the aim to identify genetic variations that could explain the genetic susceptibility to odor perception^73^. Furthermore, it is well admitted that an odor results from the perception of a mixture of molecules, which give more complexity in such classification^74^. Grouping some odors rationally, in more general categories, can improve the performance and the robustness of the GCN models.

Secondly, about the ORs, the number of compounds with known activity on ORs is still low. Mori estimated that more than 400 000 different compounds are odorous to the human nose^75^. Still, we collected only a couple of hundred of molecules with bioactivity on ORs. Increasing the number of functional ORs experiments for large set of compounds would definitively improve the quality of the models. We have noticed that some ORs are highly investigated and other less^9,76^. For example, OR1A1^77^, OR1D2^78^, OR1G1^79^, OR2W1^80^, OR2M3^81^ have been reported to be active by more than 100 compounds. At the opposite, there are 72 ORs for which only one compound has been tested active. Developing a GCN model with ORs having enough compounds tested (for example > 5) could improve the model performance on ORs. Another possibility would be to increase the chemical-OR bioactivities by studying the transcriptional profile modulation of ORs in vivo i.e., in olfactory sensory neurons (OSN) in vertebrates. Recent studies have been reported on this direction and identified the full repertoire of receptors activated by a given odorant^82,83^. Although encouraging, the number of compounds with transcriptional profile is still limited.

In third, the stereochemistry of a molecule is may be not optimal in our data set. It has been reported that stereoisomers of a chemical can be related to different odors^84,85^. For example, the R-carvone is related to minty odor while its enantiomer, the S-carvone, has a caraway odor^77^. Although enantiomeric compounds have similar chemical functions, it has been reported that as few as 5% of enantiomer couples have a similar smell^86,87^. It is possible that the racemic form of some of the compounds, used in this study, has been considered and it might cause a mis classification to some odors.

About machine learning approaches, CNN and GCN are the latest and powerful machine learning approaches. GCN seems to outperform CNN and RF in our study. Many odorant-odor notes models have been described recently. Sharma et al. have reported a model based on 5185 chemical and 542 smell using a Deep Neural Network (DNN) algorithm with promising results^42^. The performance is a little lower with a AUROC = 0.76. However, one advantage of DNN is, it automatically identifies optimal features overcoming the problem of feature selection. On a more restricted data set (476 chemicals and 21 odor notes), Keller et al. obtained an AUROC of 0.83 based on a Random Forest method^39^ and Sanchez-Lengeling et al. described a GNN model with an AUROC = 0.89 using 5030 chemicals and 138 smells^43^. Models based on olfactory receptors are more limited. Kowalewski et al. developed a SVM model using 150 odorants and 34 human olfactory receptors with an AUC = 0.88^40^. Recently, a conglomerate of artificial intelligence driven prediction engines for olfactory decoding was reported, including odorant-OR interactions predictions based on structure-based approaches^88^. The models showed good performance with an AUC = 0.87 for ORs and an AUC = 0.94 for smell based on DNN methods.

Overall, these results illustrate the potential of artificial intelligence to decipher the relationship of odorant molecules with olfactory receptors and smell perception. Associating to several previous studies carried out by other research groups^18,39–41^, our study provides an increase in the knowledge of the links between odor notes, molecular structures of odorants and target olfactory receptors of mammals. Especially, thanks to largest data as well in number of odorants than in number of olfactory receptors, we show that our model is able to correctly connect numerous pairs odorant-OR, and now to predict other new pairs.

However, models based on artificial intelligence can show some limits with odors and receptors that are not well represented by chemicals. As recently pointed by Gerkin^89^, it is necessary to use a large volume of odorant molecules with the corresponding odorant description as several as odor notes (or odor attributes). Moreover, the molecular properties of the odorants must be described by a large number of molecular descriptors able to report all their structural characteristics.

Expanding the knowledge of our sense of smell by combining different sources of data from chemical biology (proteome-transcriptome) and human perception with advanced computational approaches will move forward the identification of the complete olfactory repertoire associated to the human smell perception.

## Supplementary Information

Supplementary Information 1.

Supplementary Information 2.

Supplementary Information 3.

Supplementary Information 4.

Supplementary Information 5.

Supplementary Information 6.

Supplementary Information 7.

Supplementary Information 8.

Supplementary Information 9.

Supplementary Information 10.

## Data Availability

The datasets compiled in this study are available for the scientific community in supplementary Table S1. We hope that it will be a good resource for further investigations.
