# Supplementary material for: Application of artificial intelligence to decode the relationships between smell, olfactory receptors and small molecules
Source: Sci Rep. 2022 Nov 5;12:18817. doi: 10.1038/s41598-022-23176-y (PMC9637086; doi:10.1038/s41598-022-23176-y)

**Figure S1**: Radar plots based on the frequency of 64 physicochemical properties observed on the set compounds associated to an odor. Each radar plot corresponds to one odor.


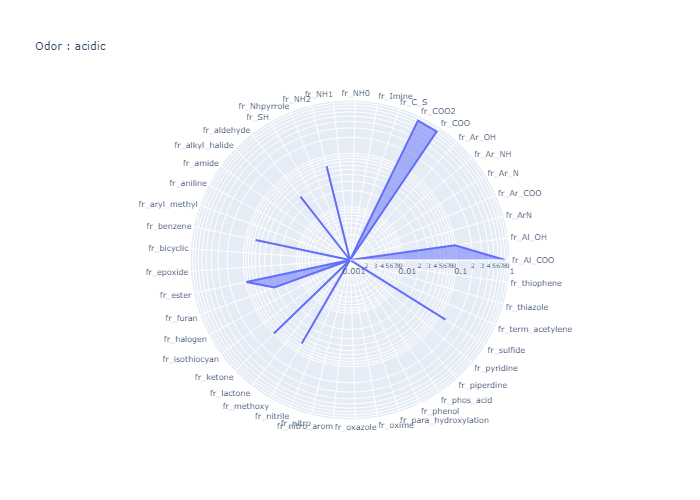

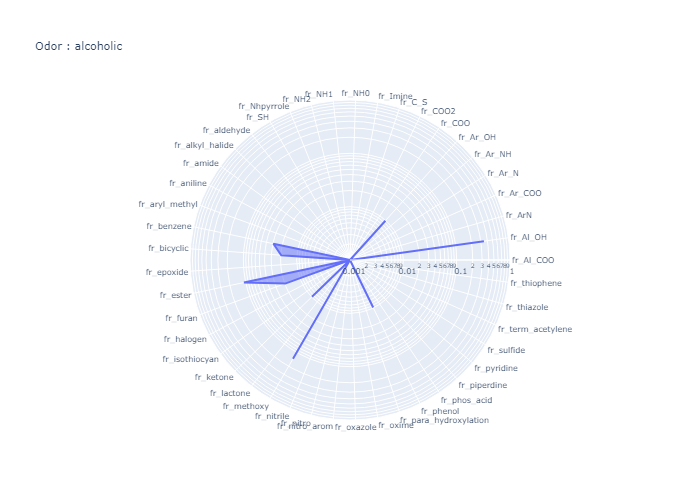


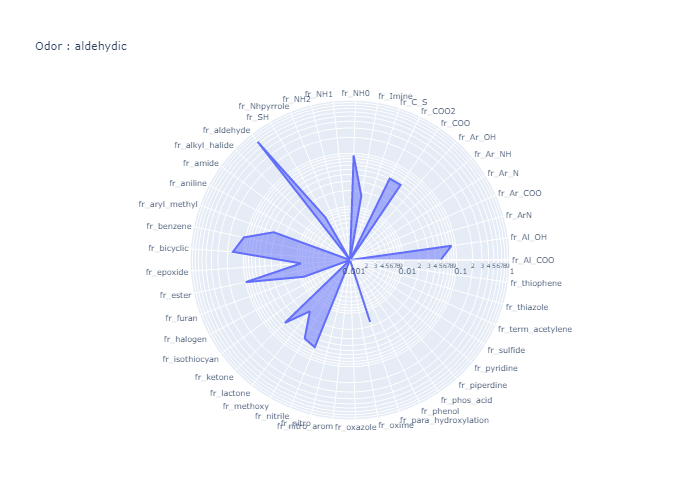

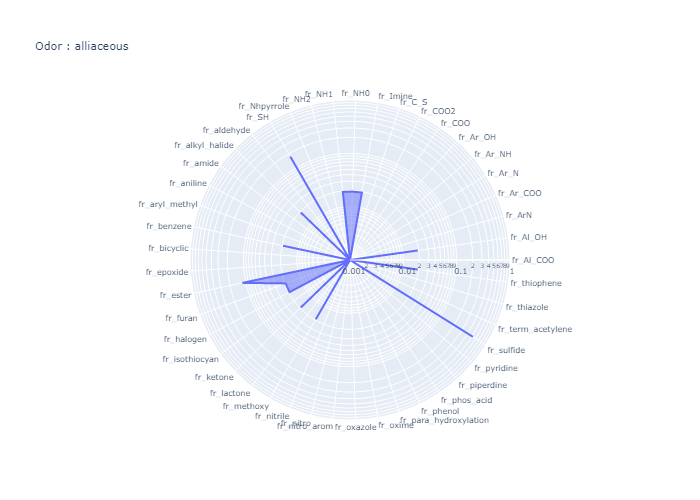


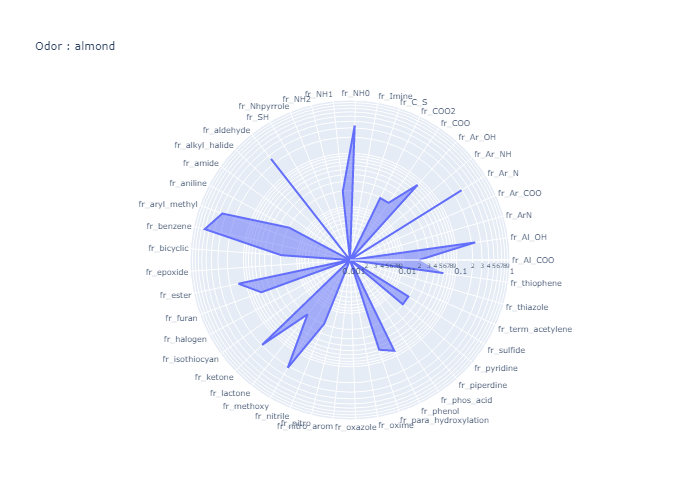

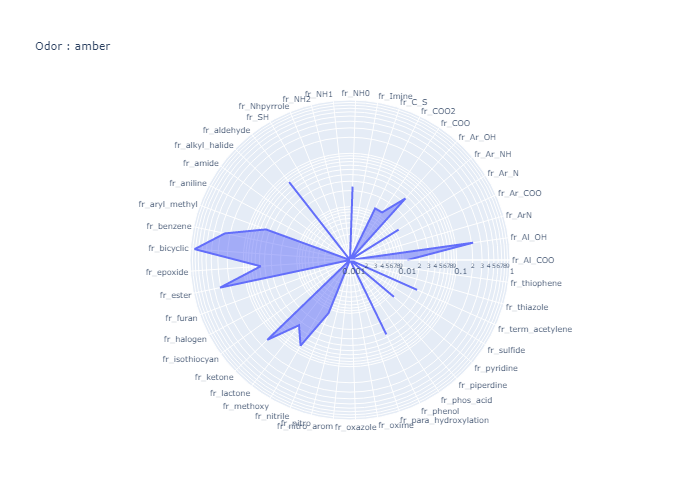


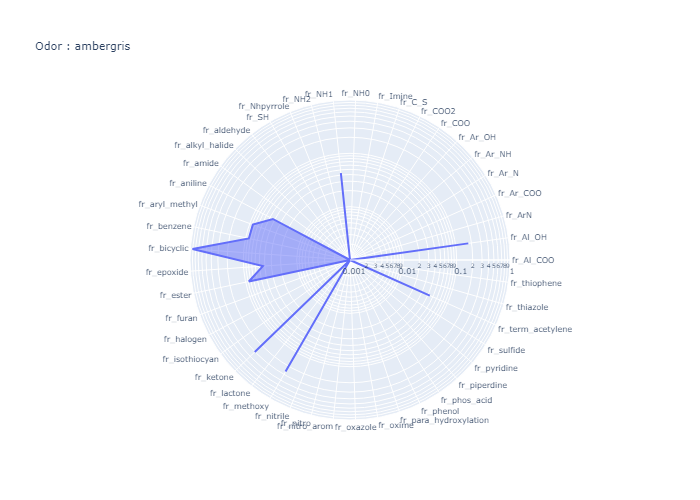

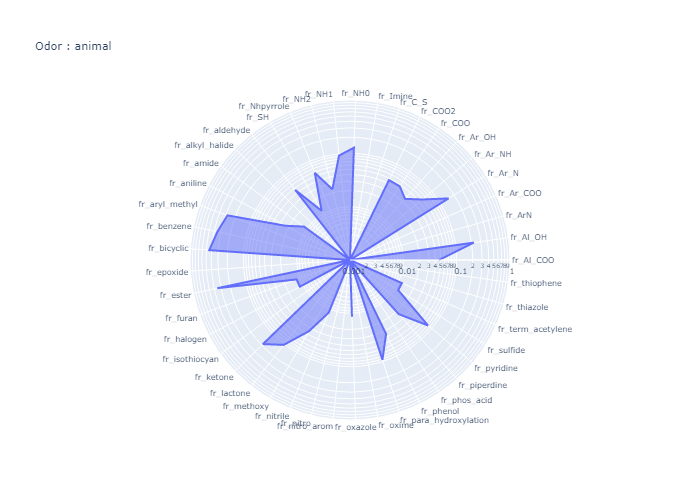


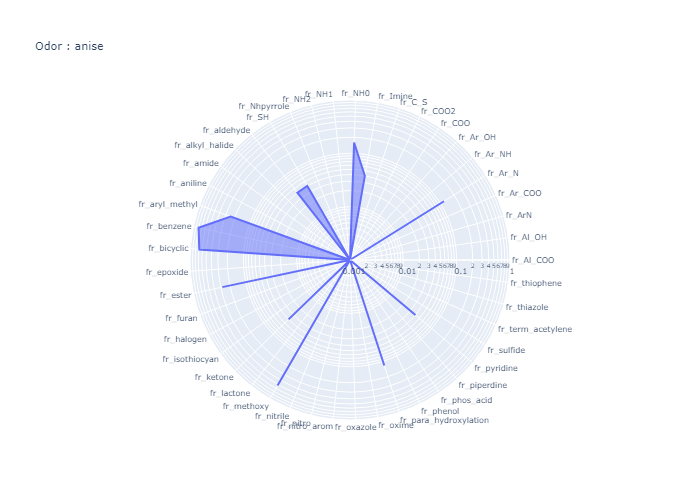

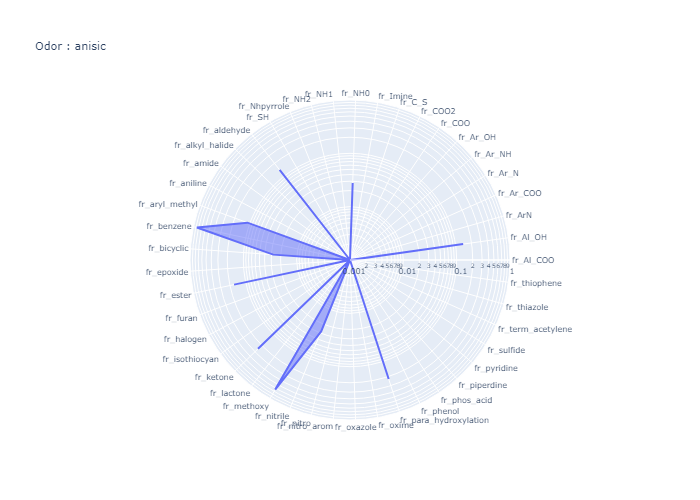


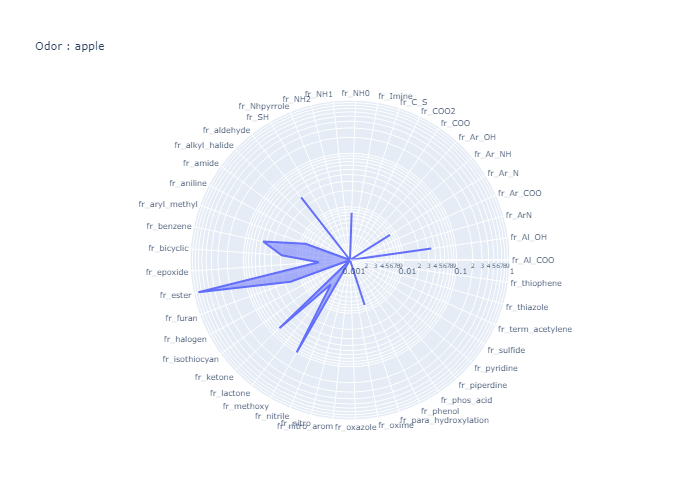

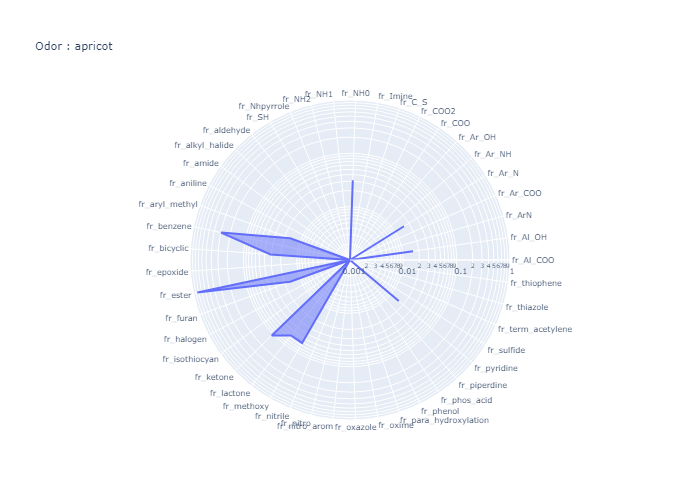


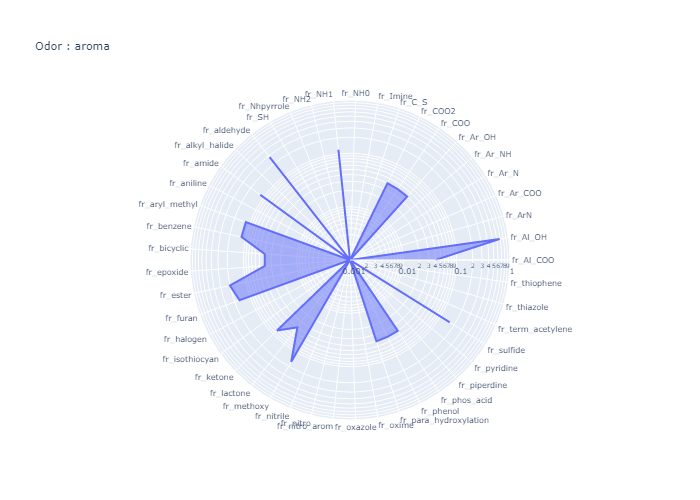

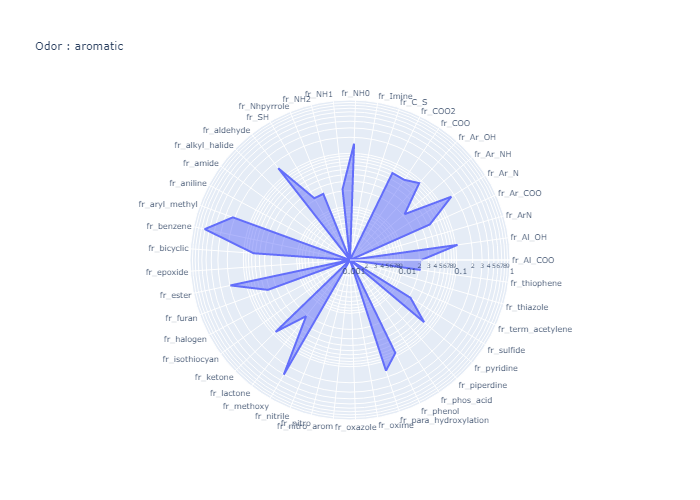


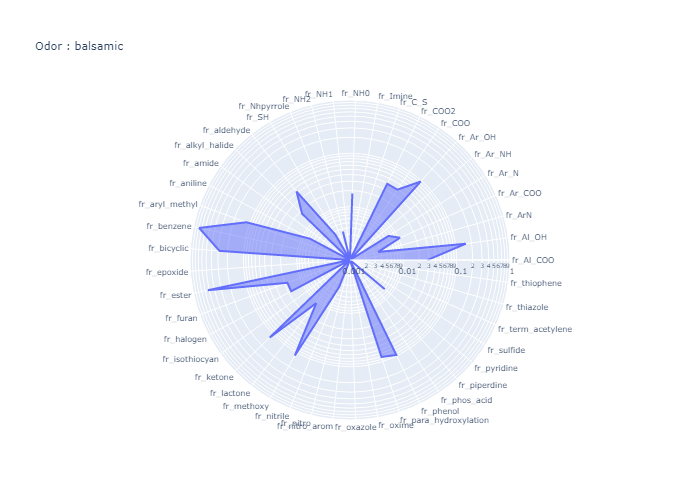

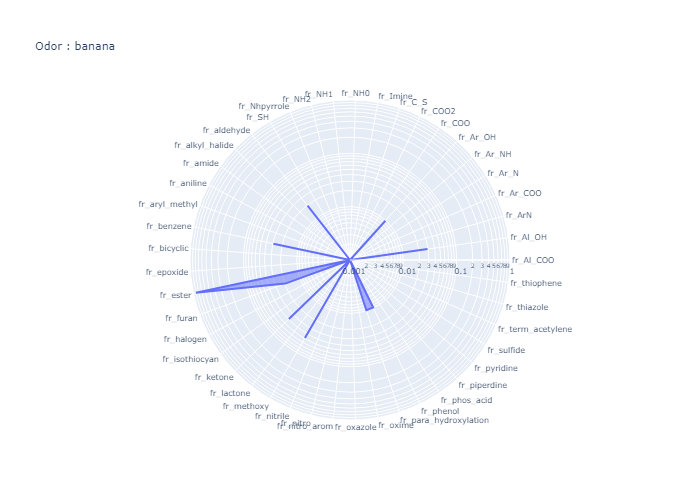


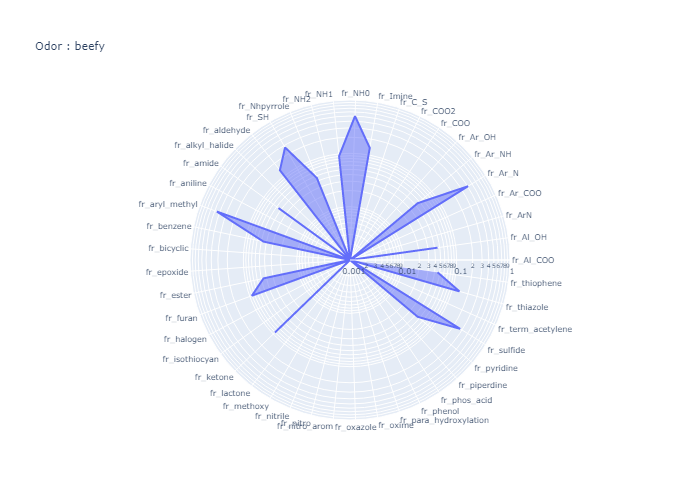

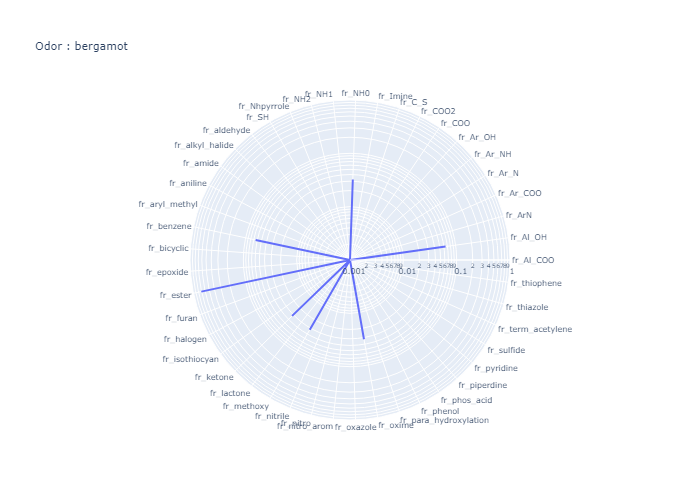


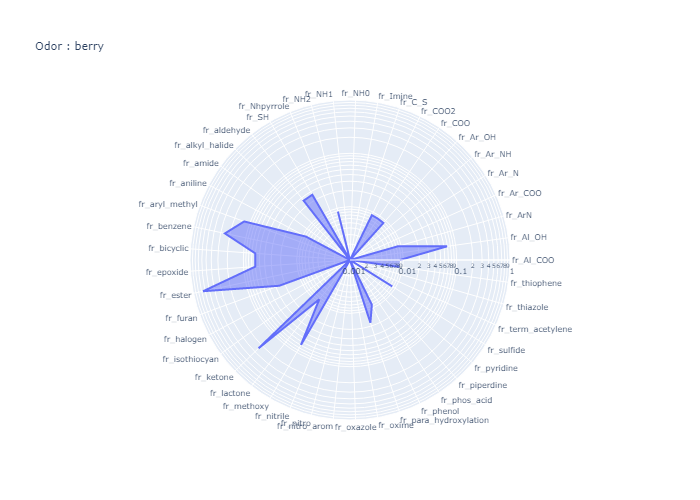

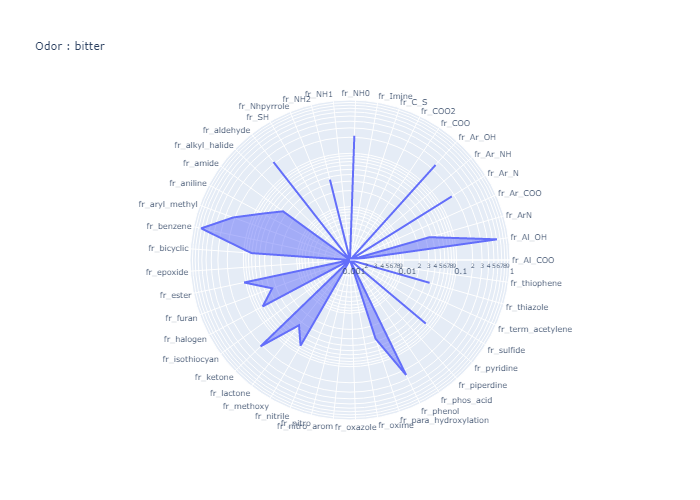


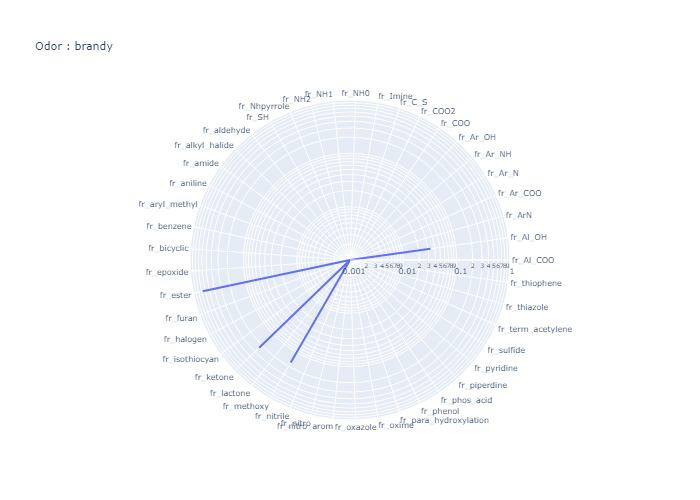

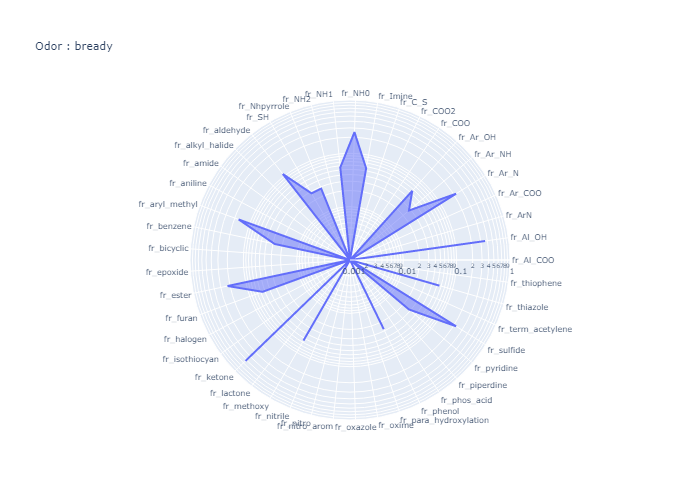


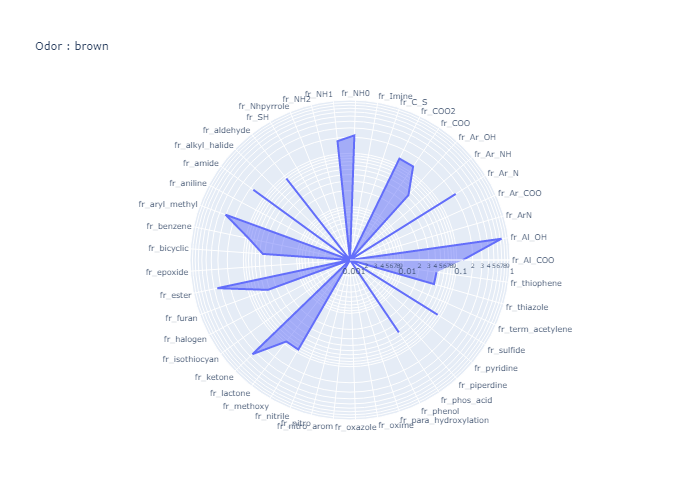

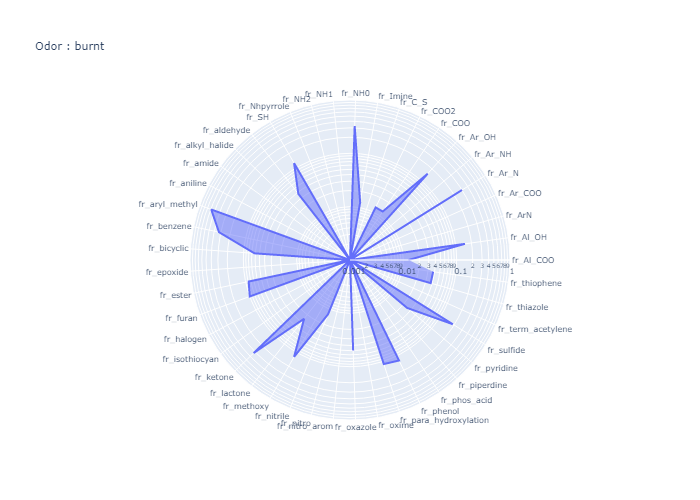


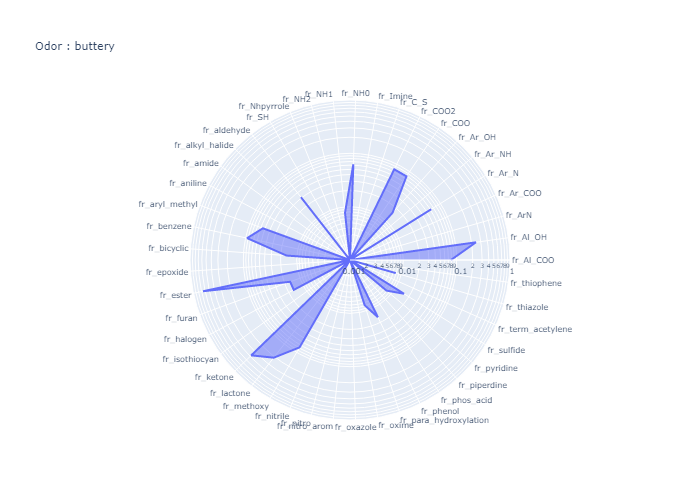

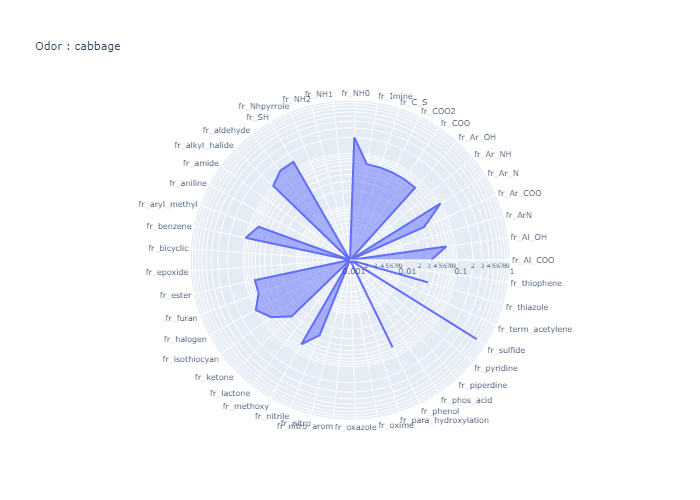


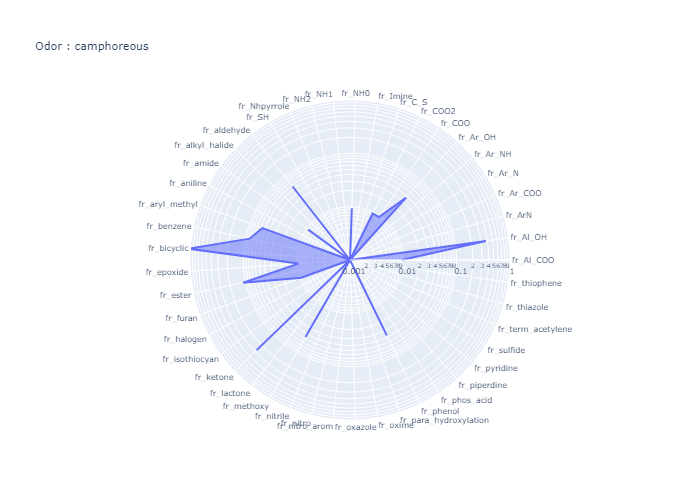

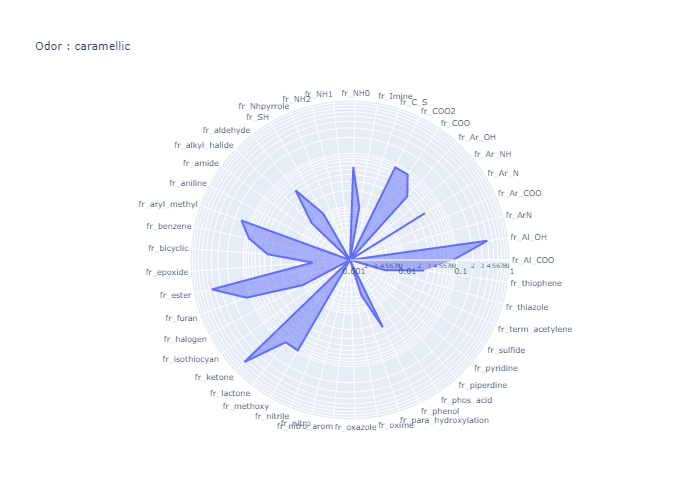


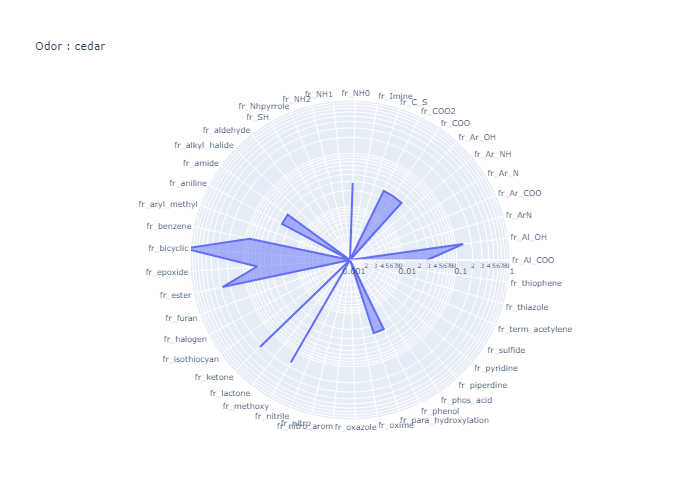

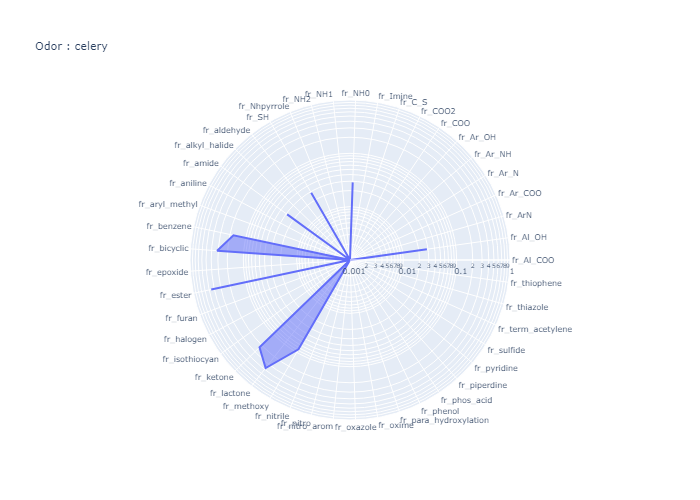

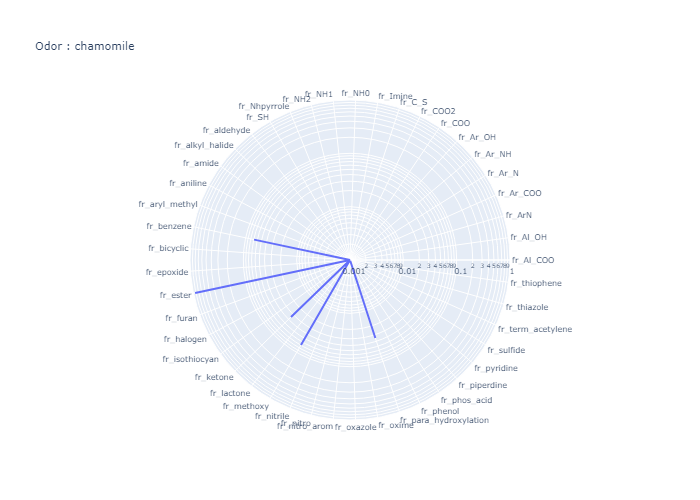

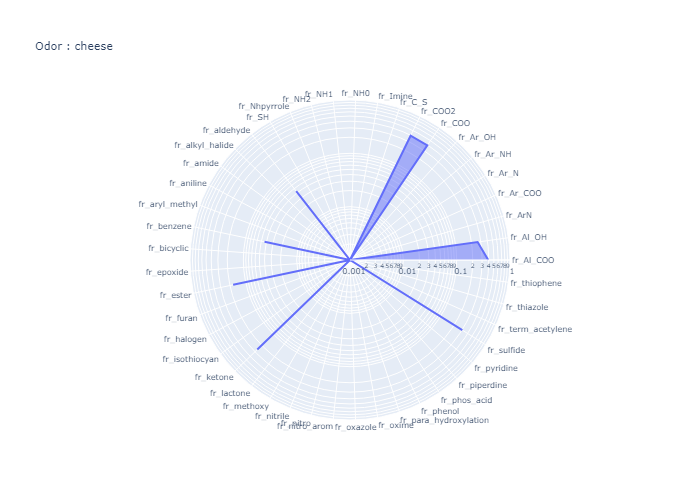


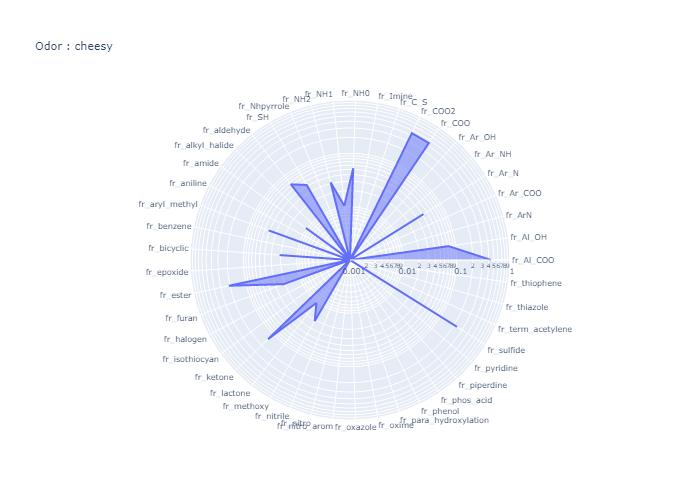

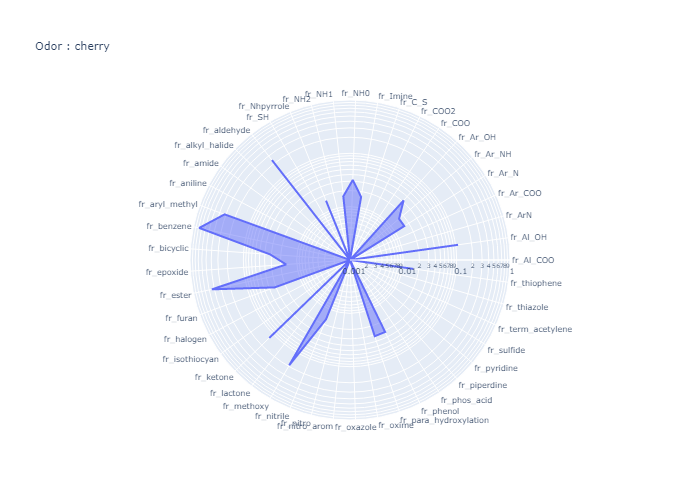


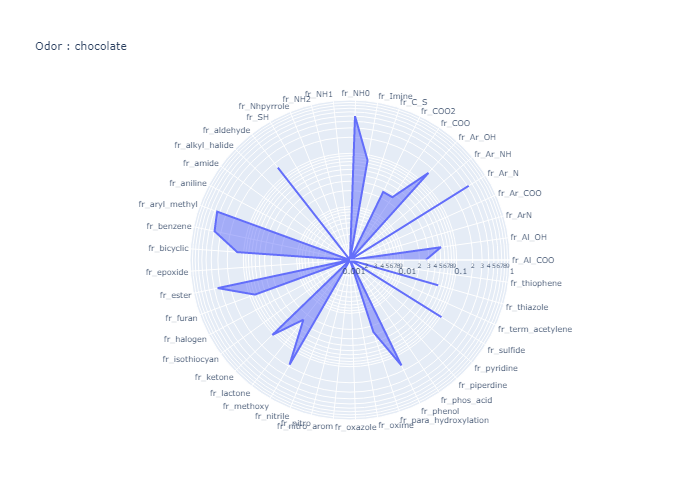

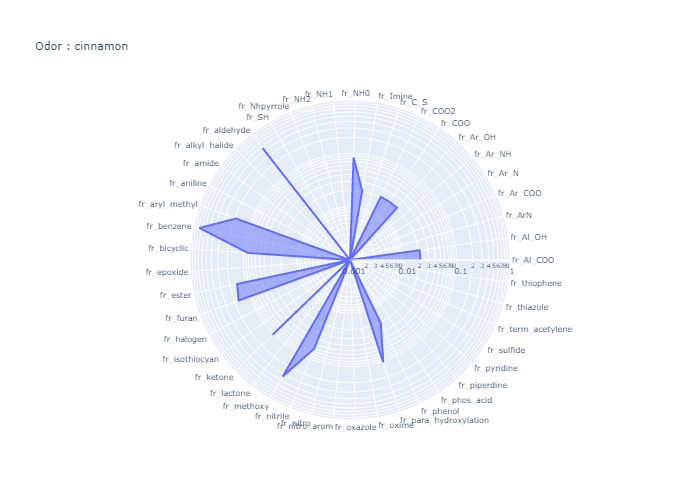


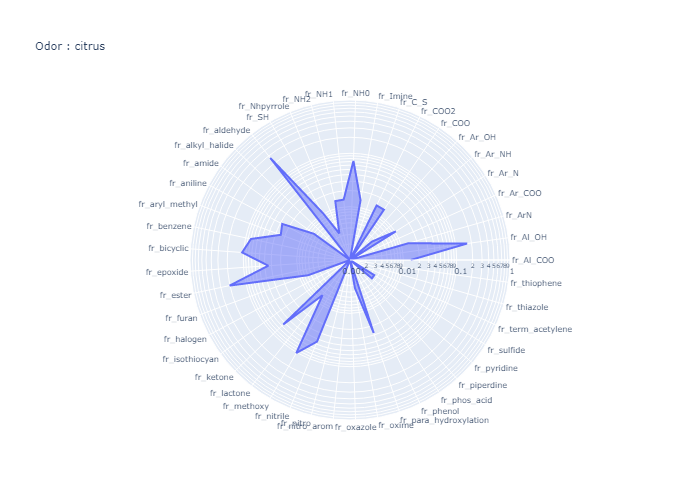

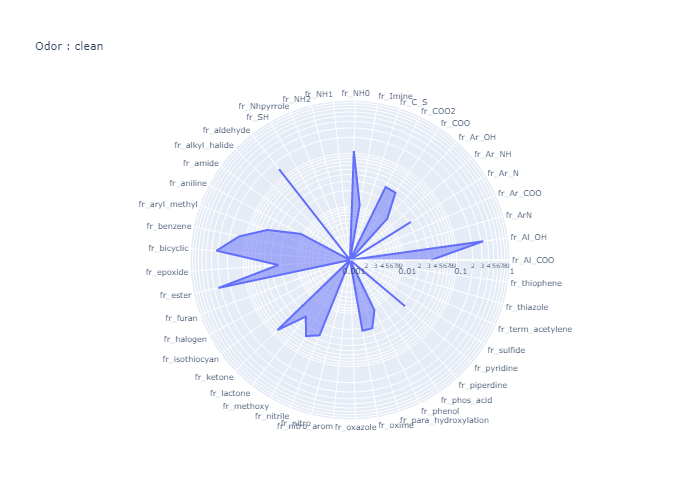


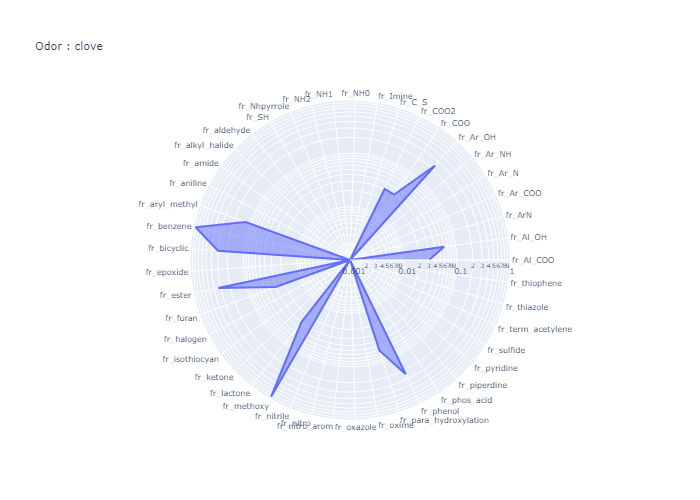

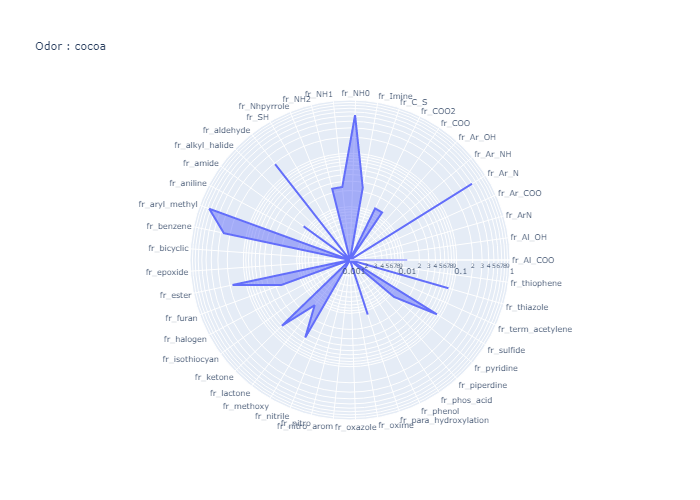


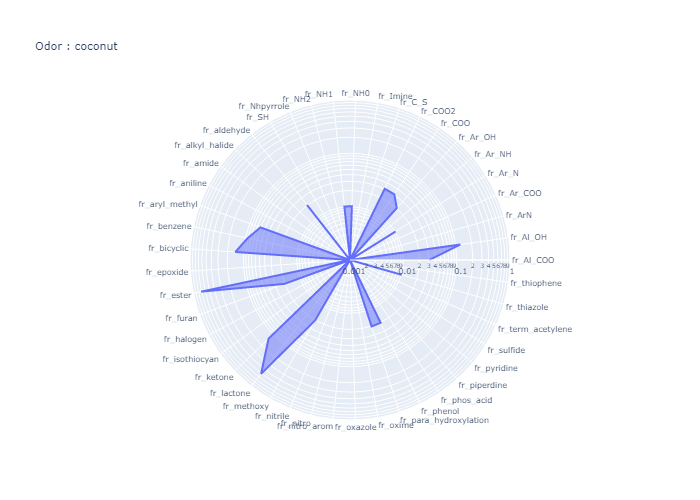

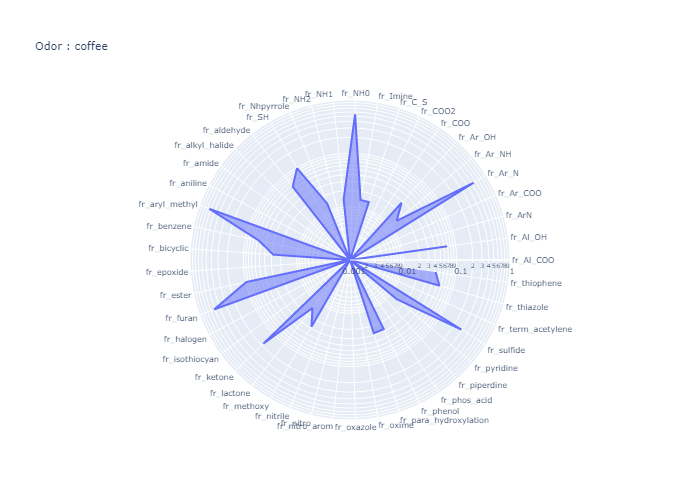


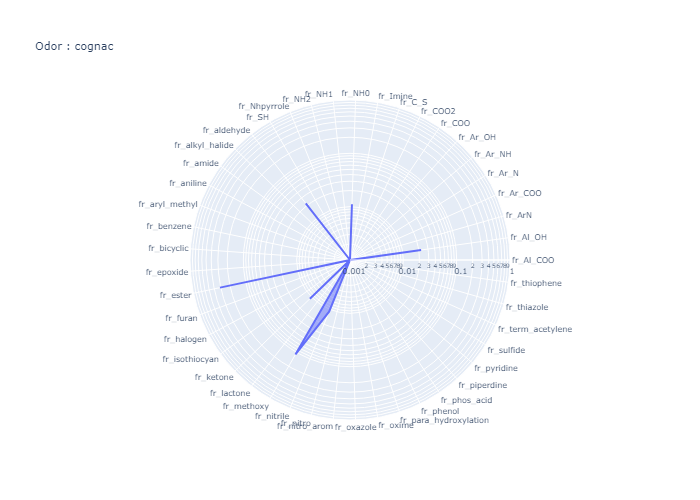

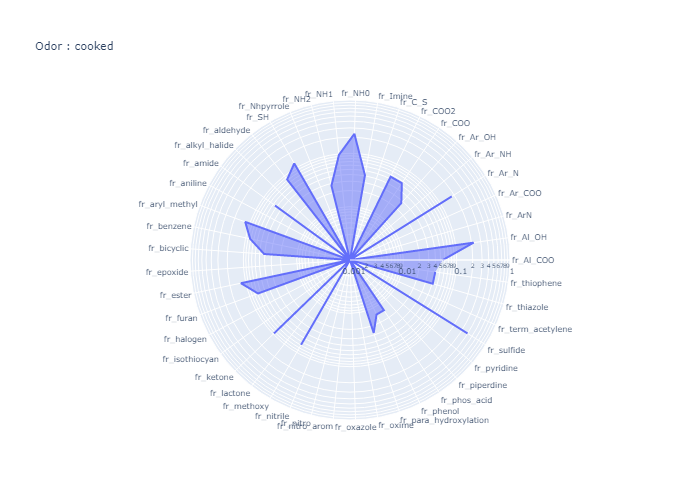


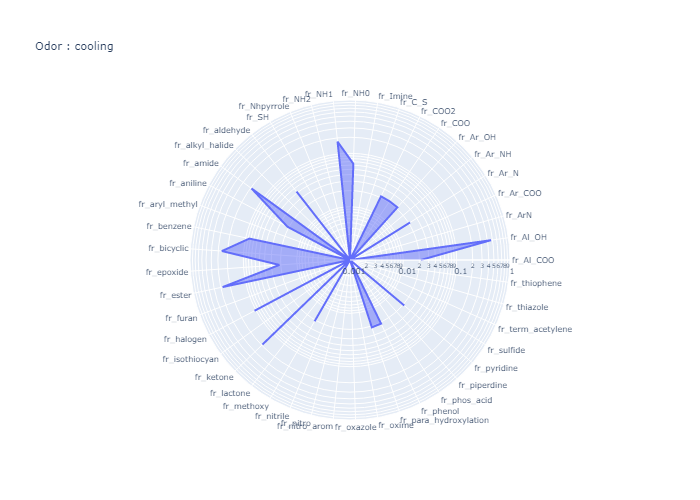

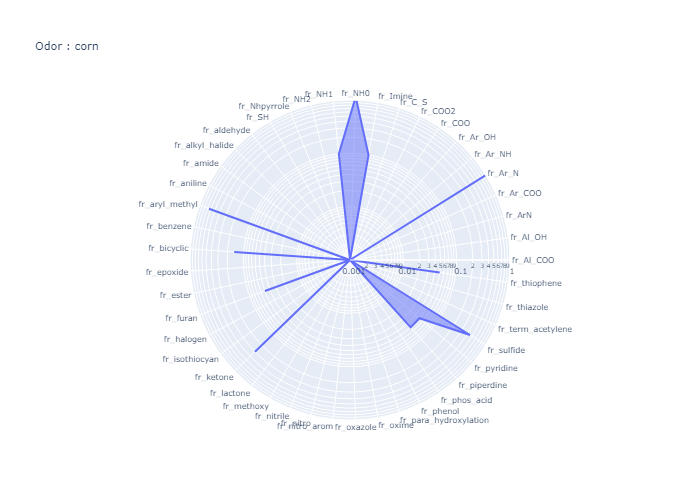


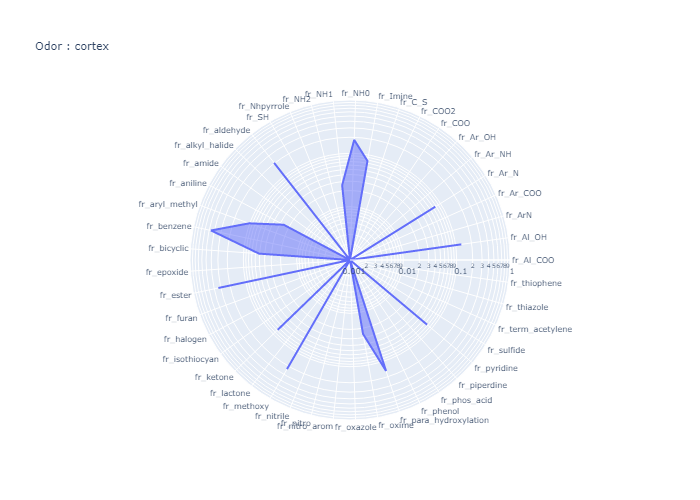

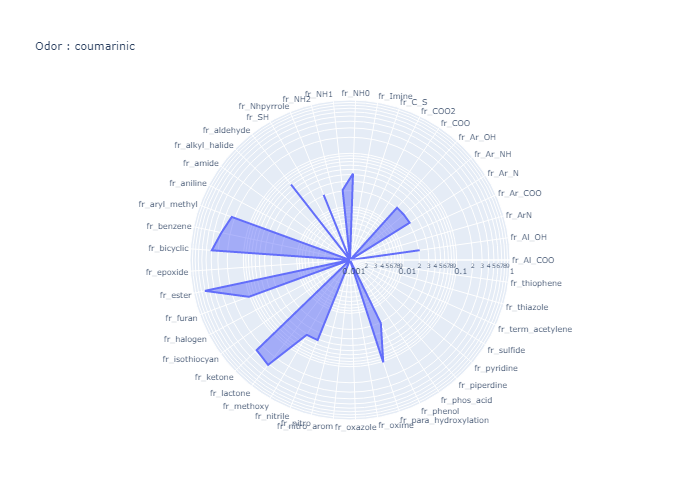


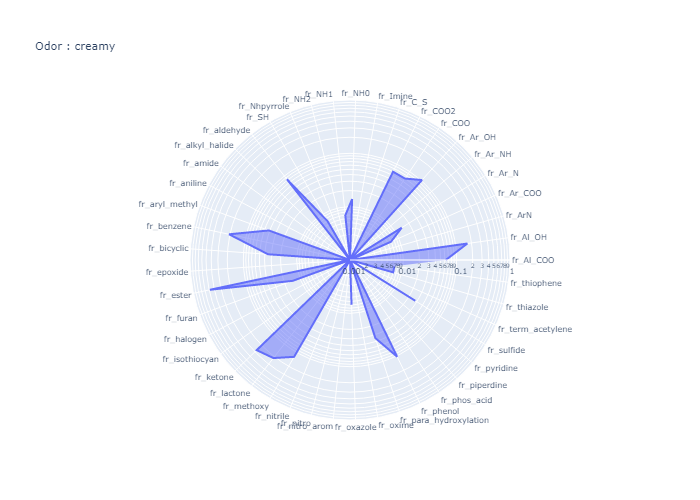

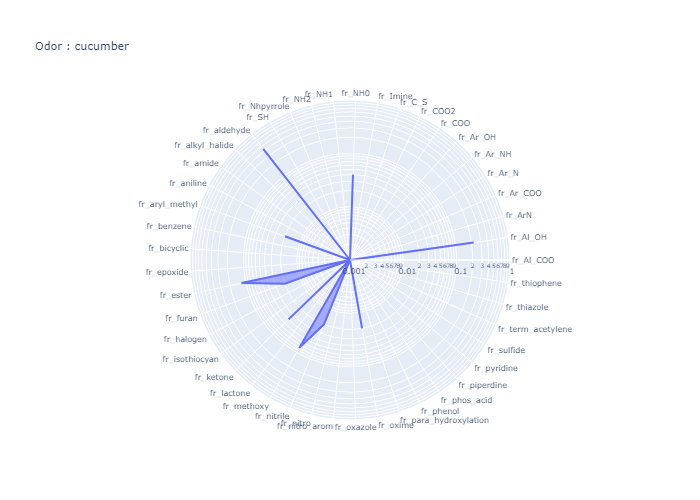


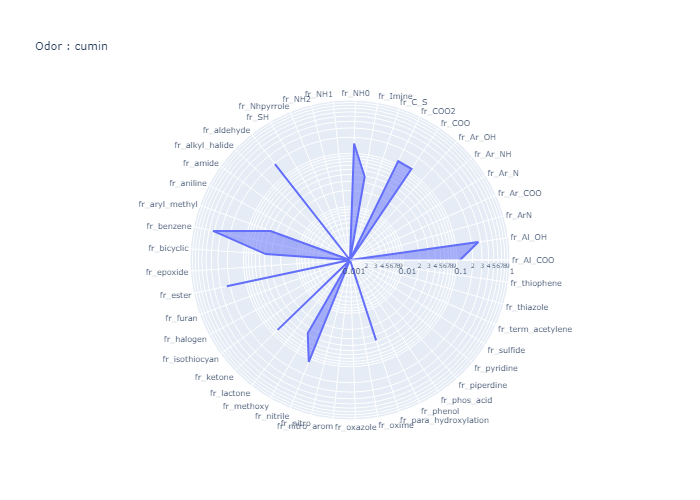

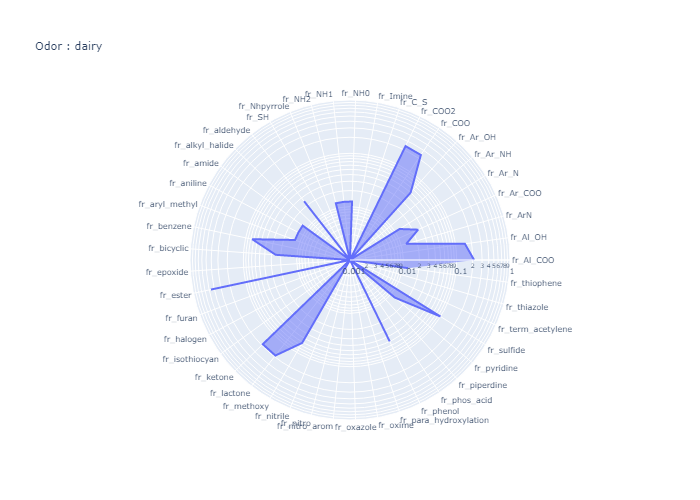


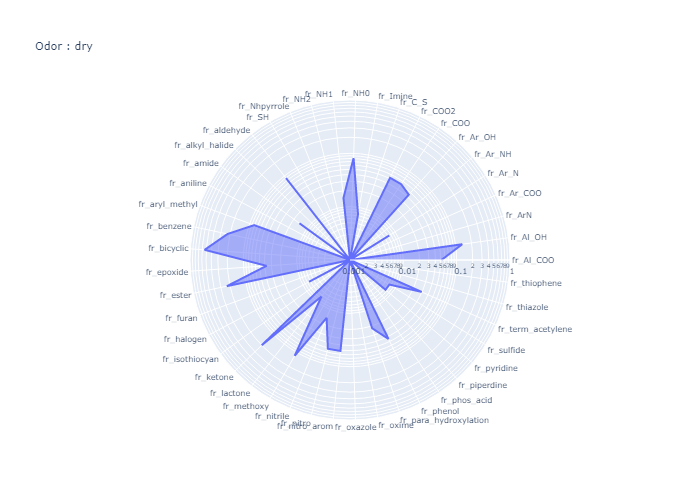

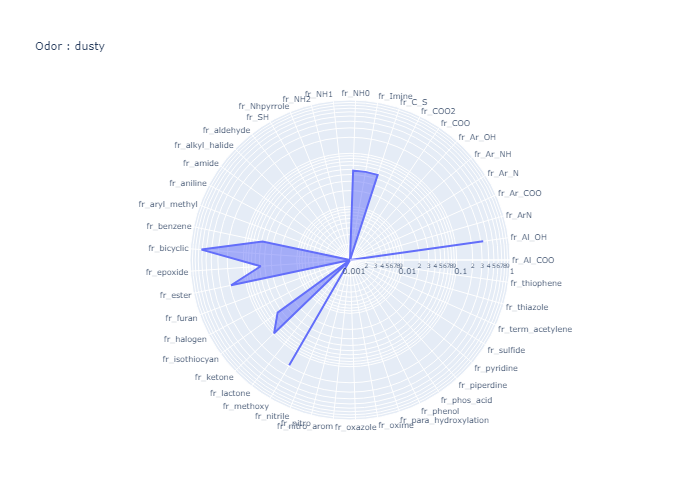


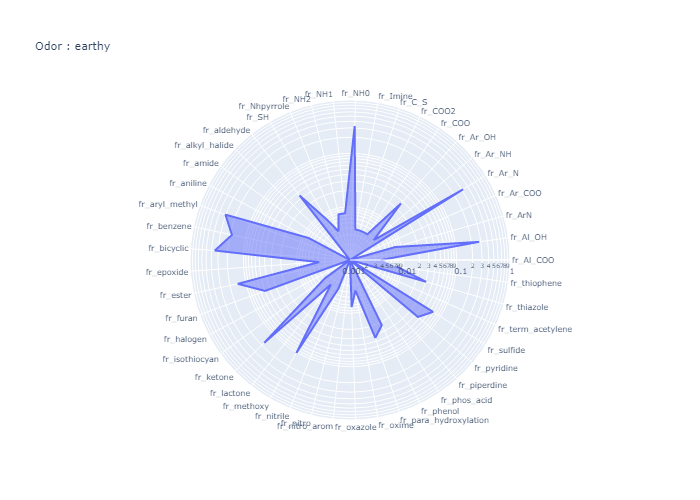

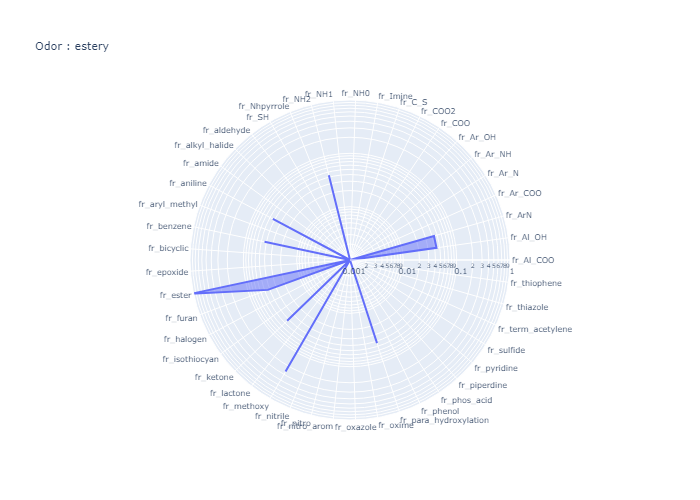


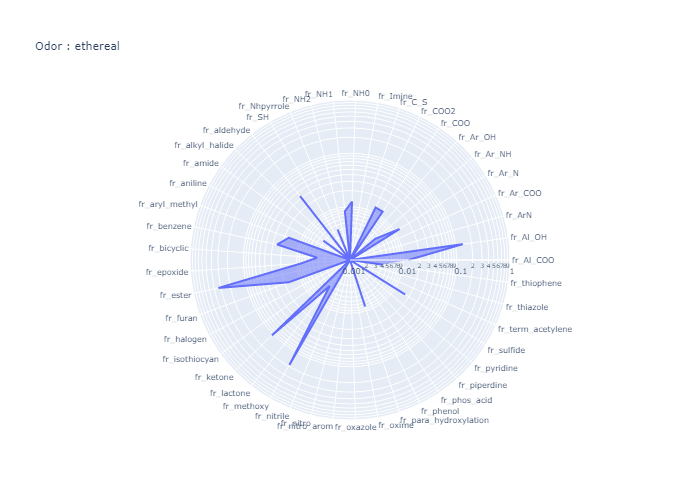

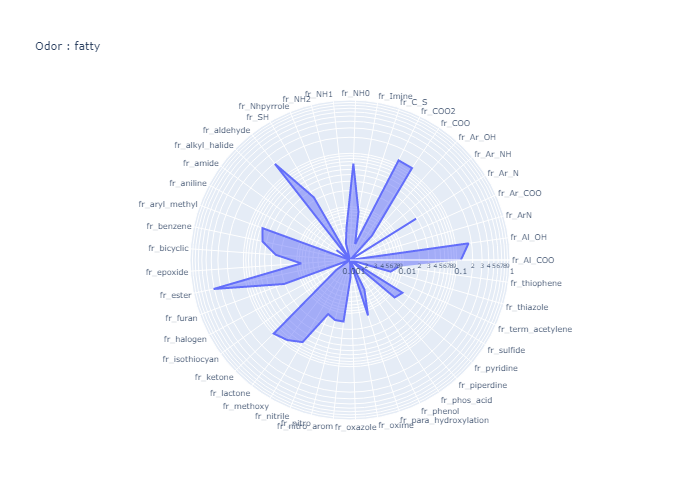


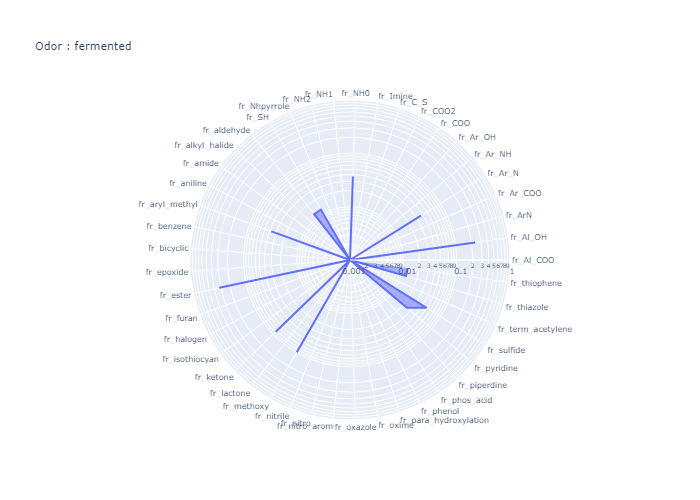

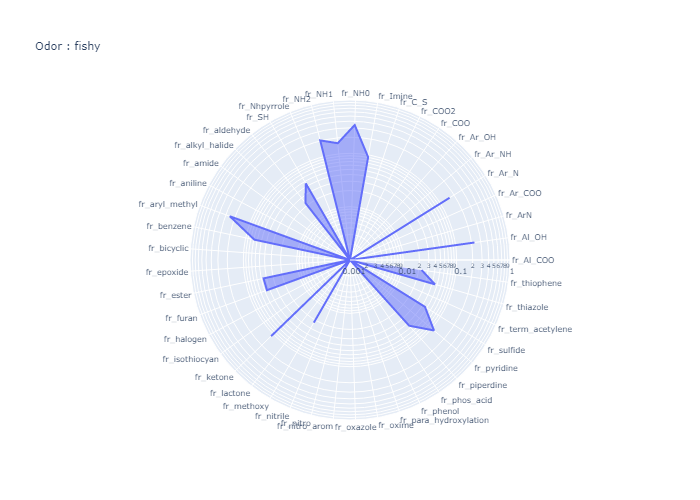


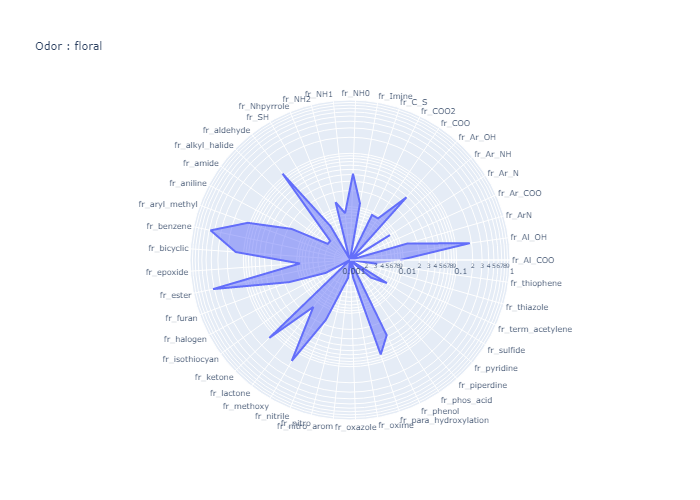

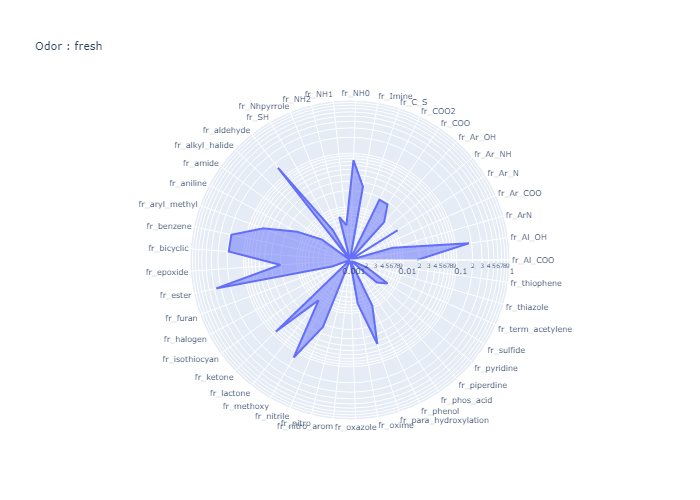


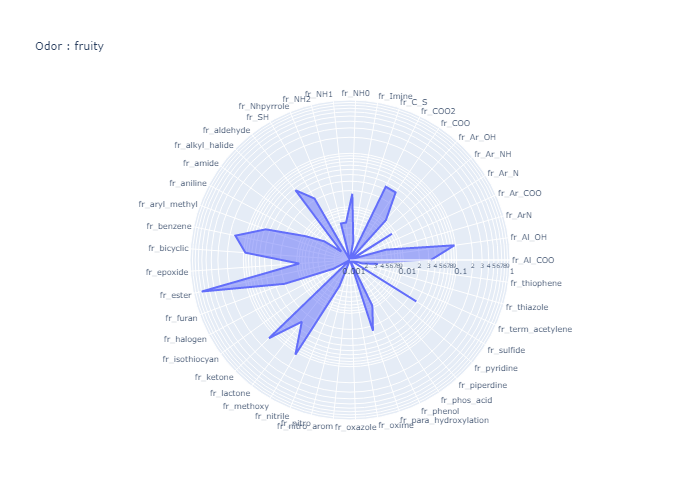

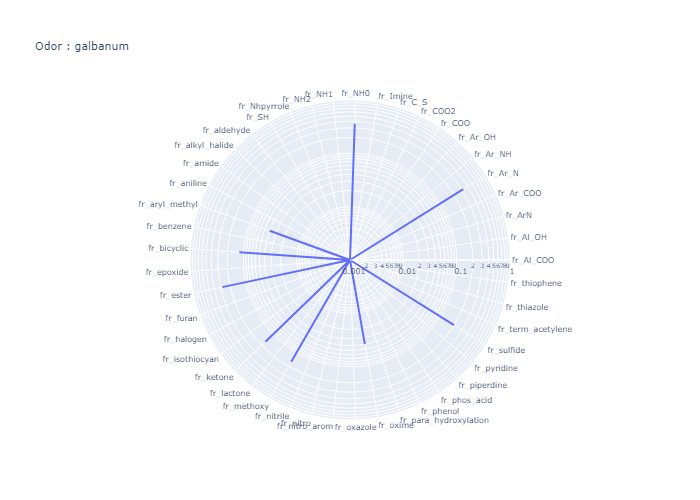


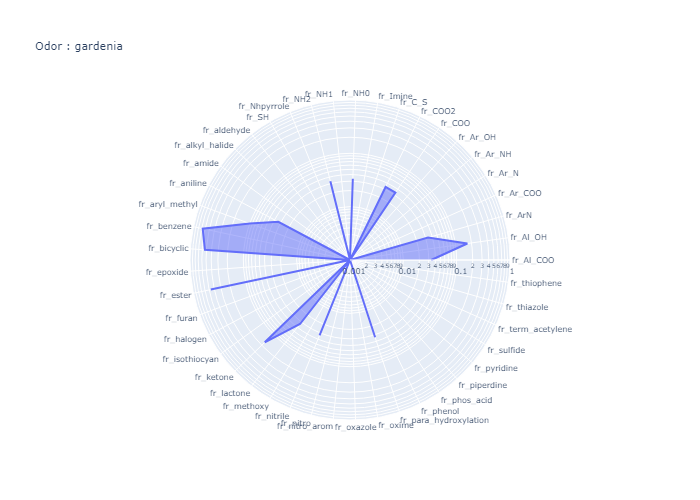

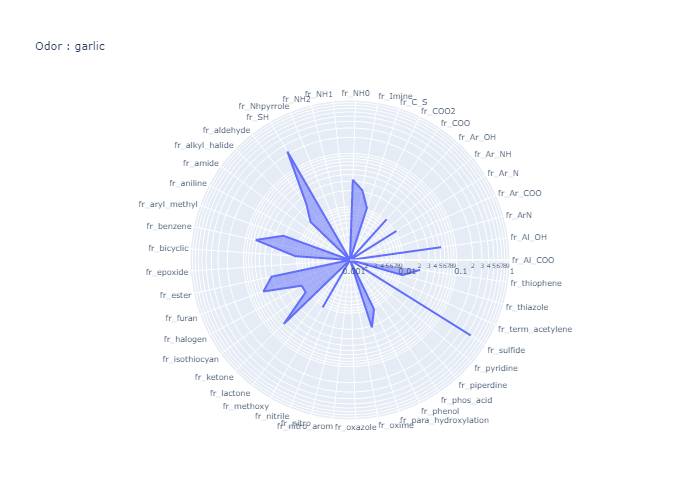


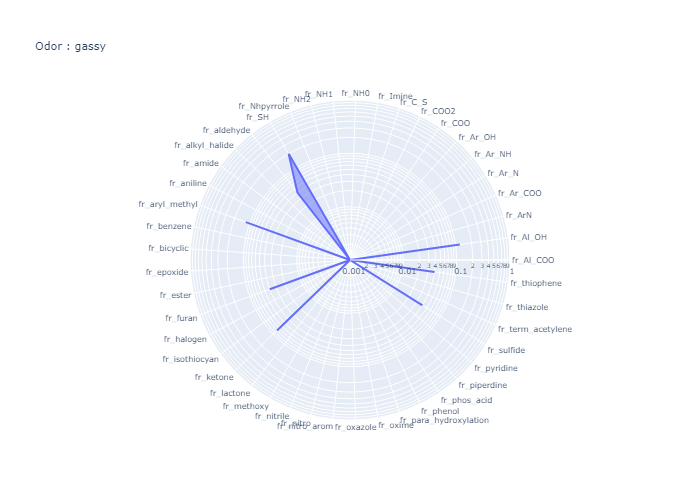

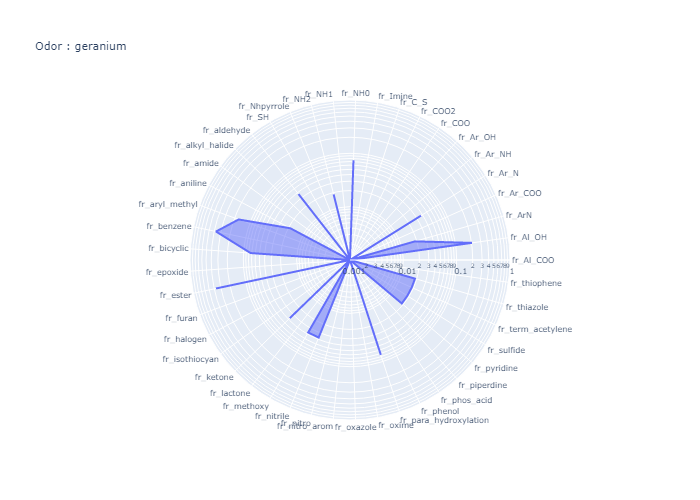


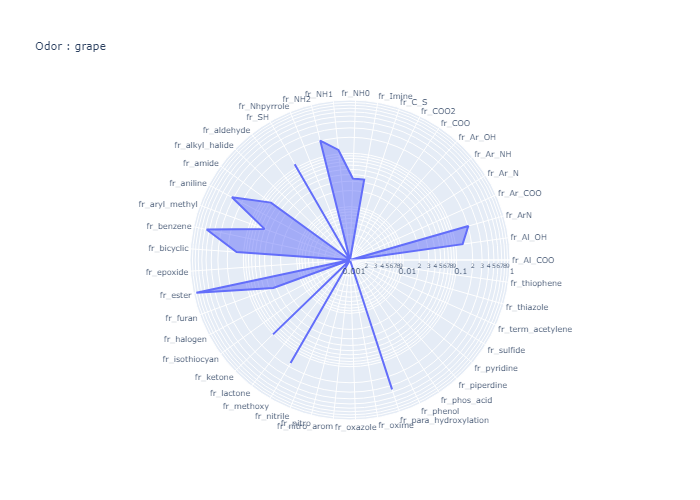

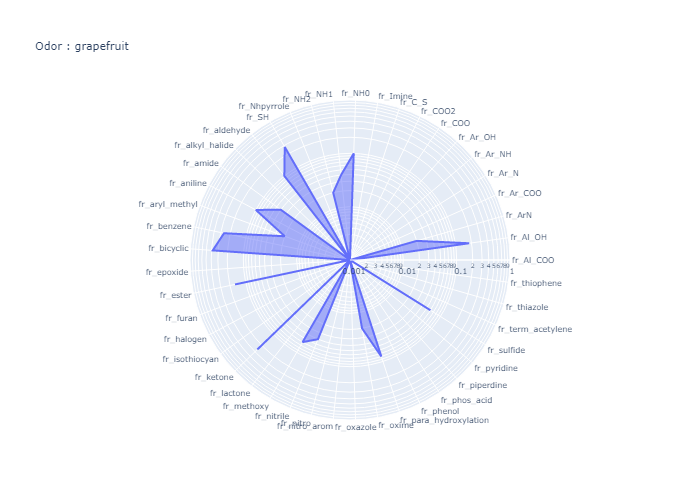


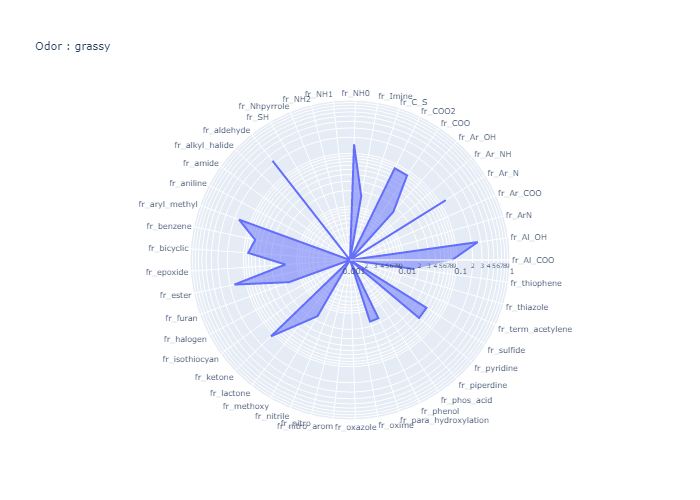

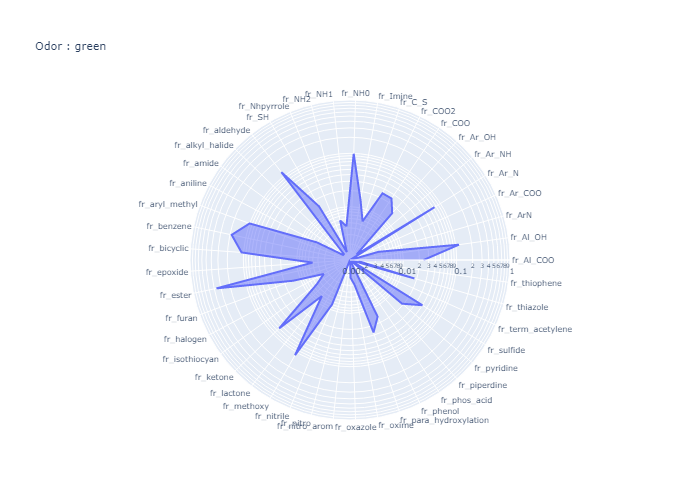


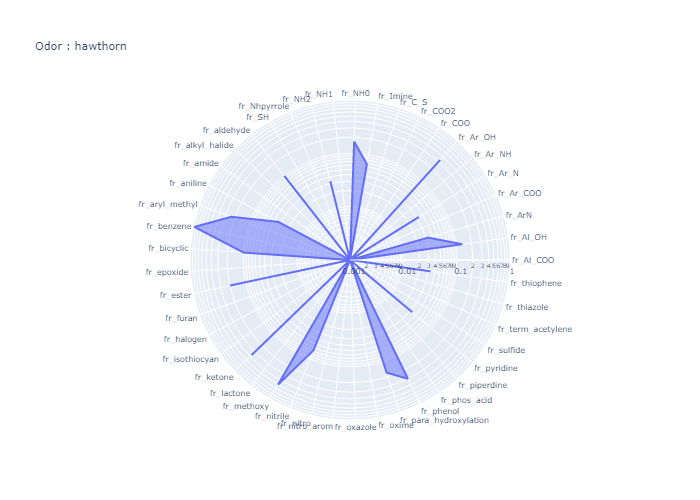

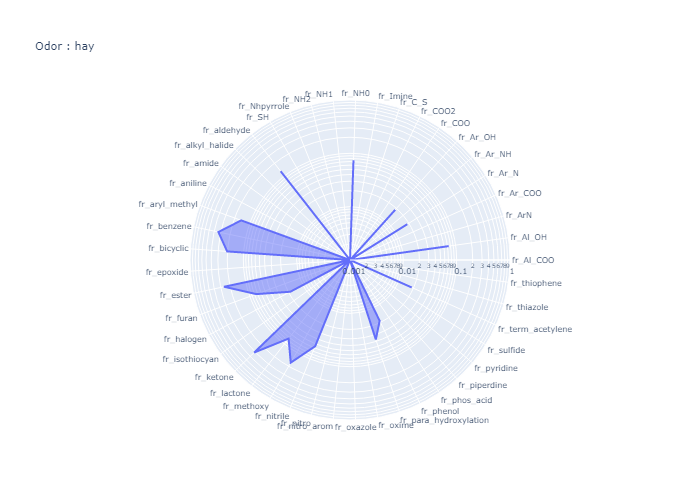


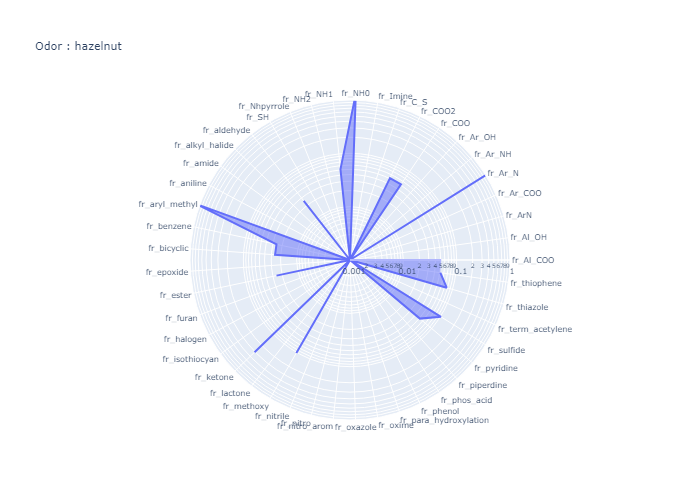

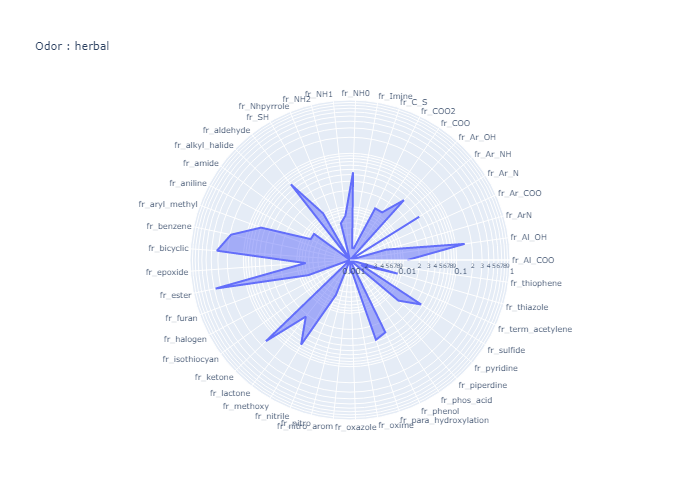


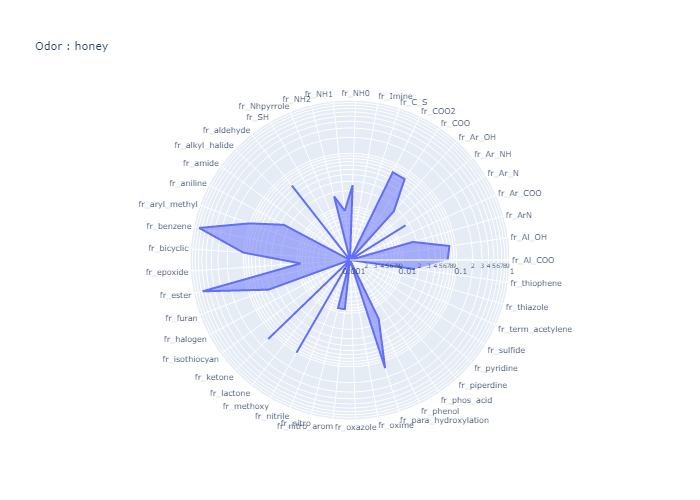

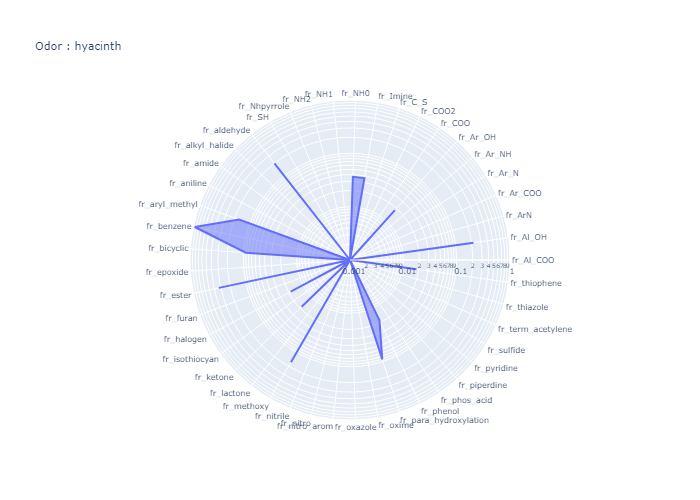


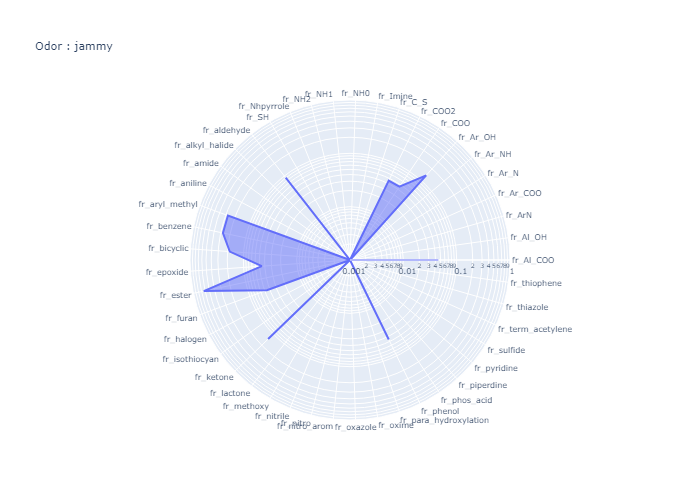

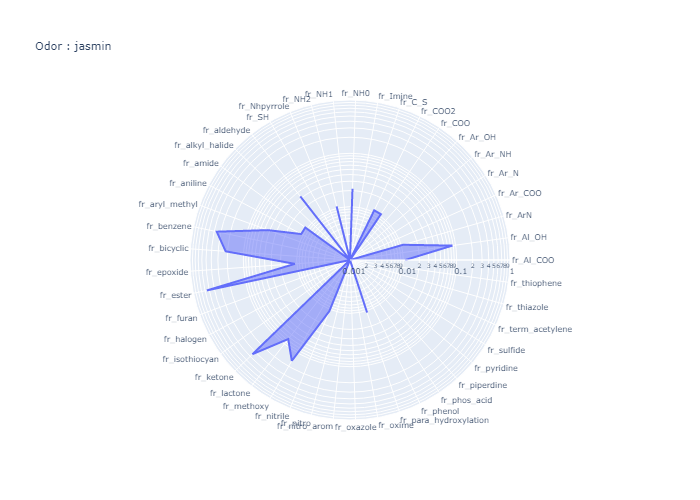


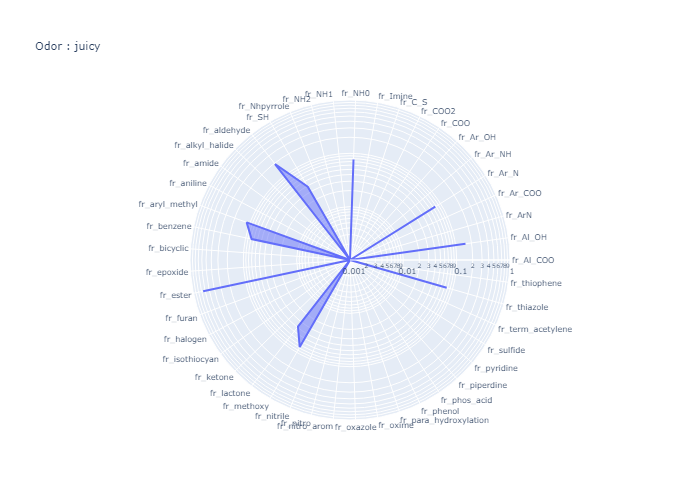

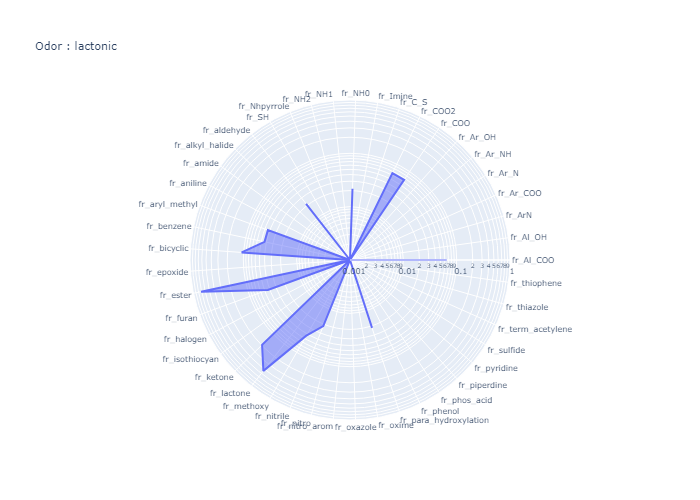


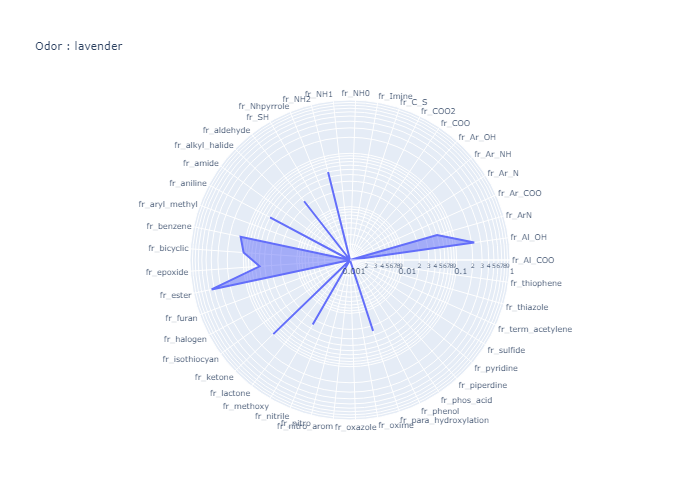

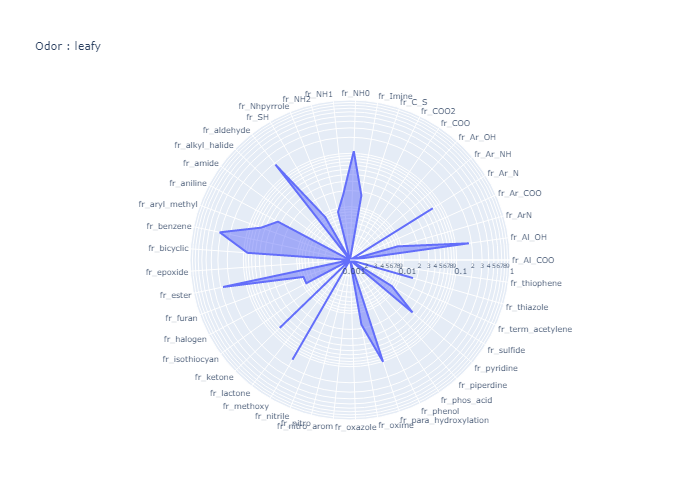


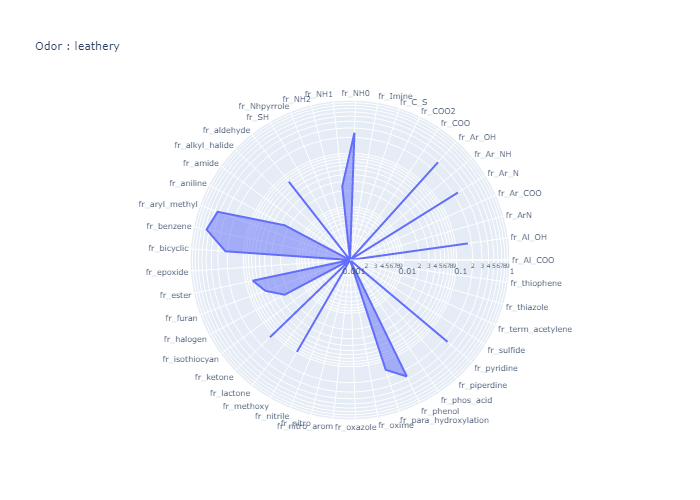

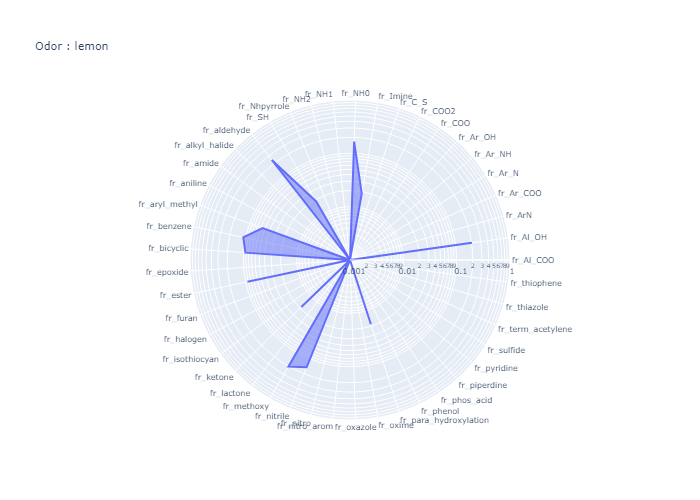


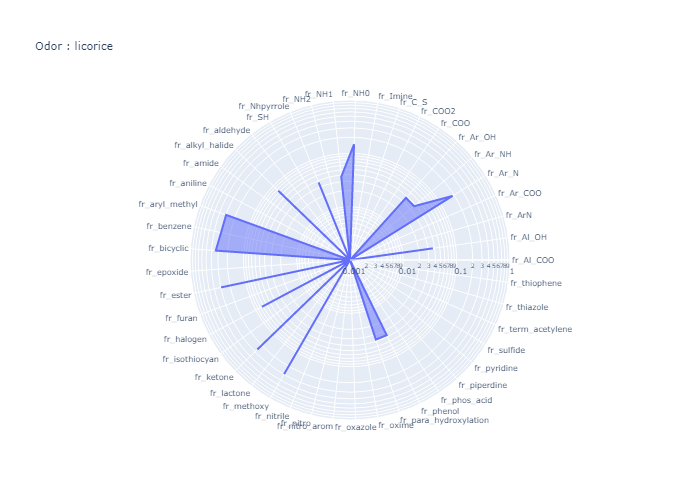

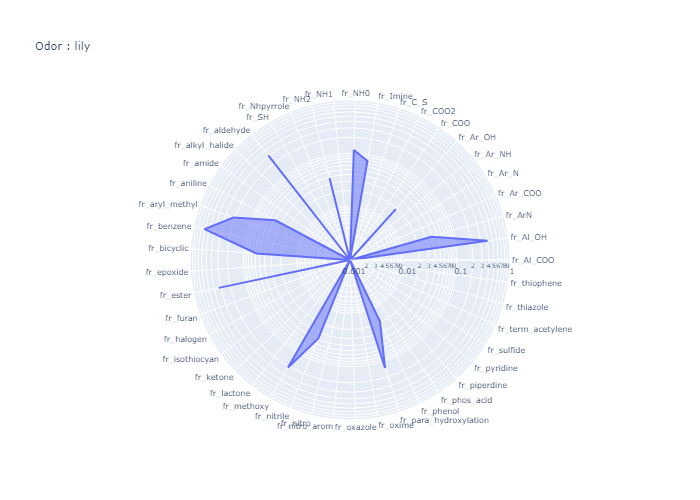


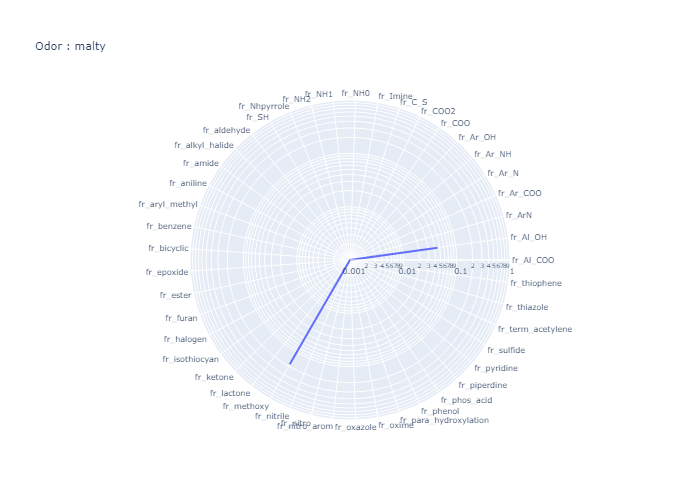

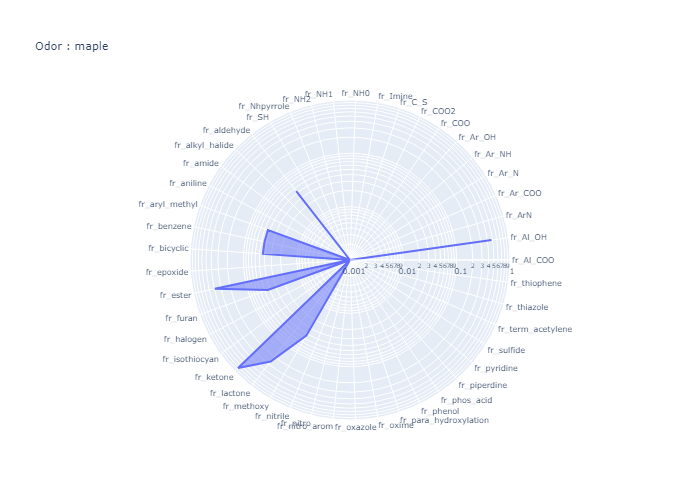


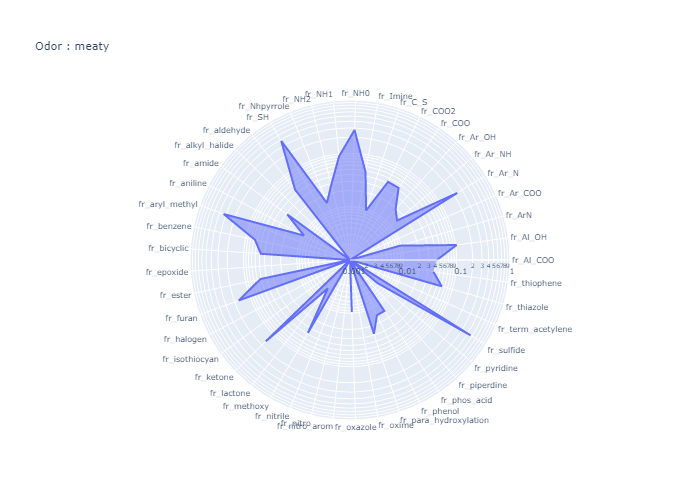

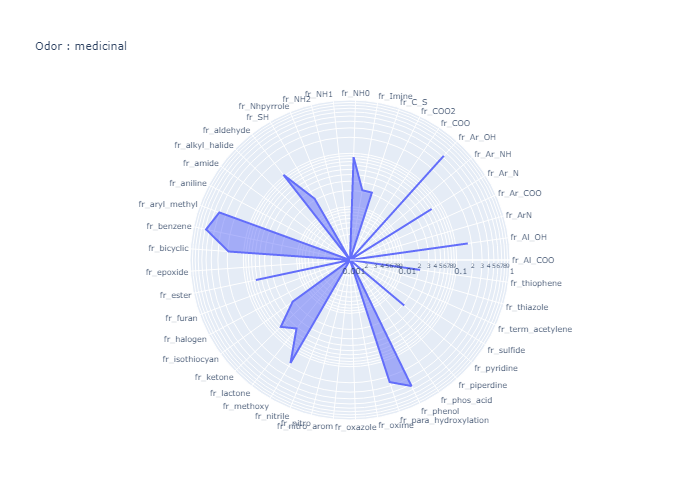


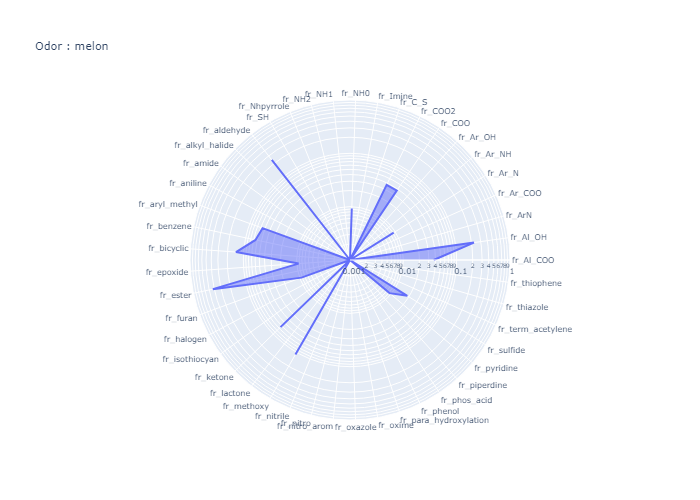

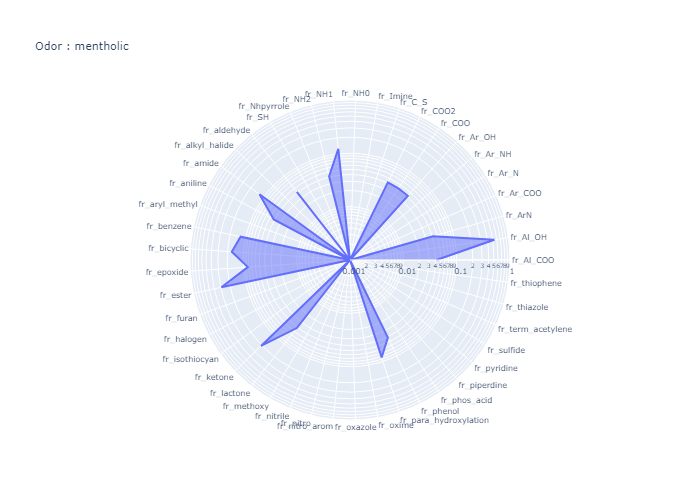


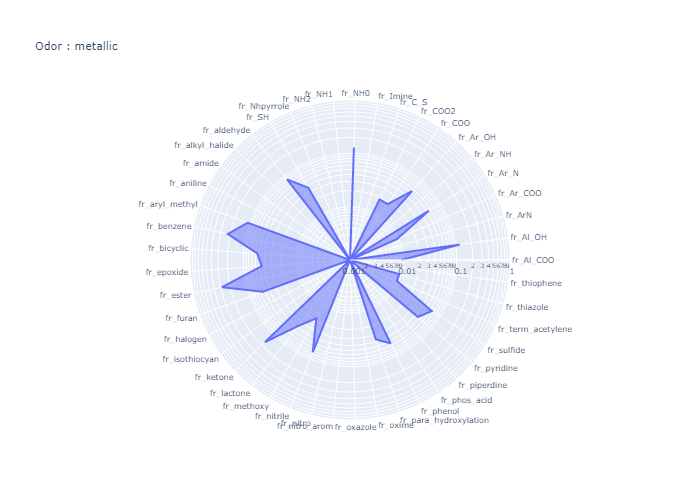

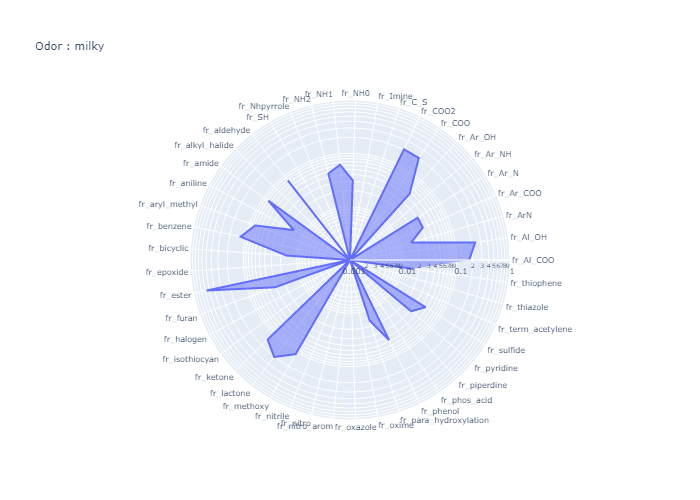


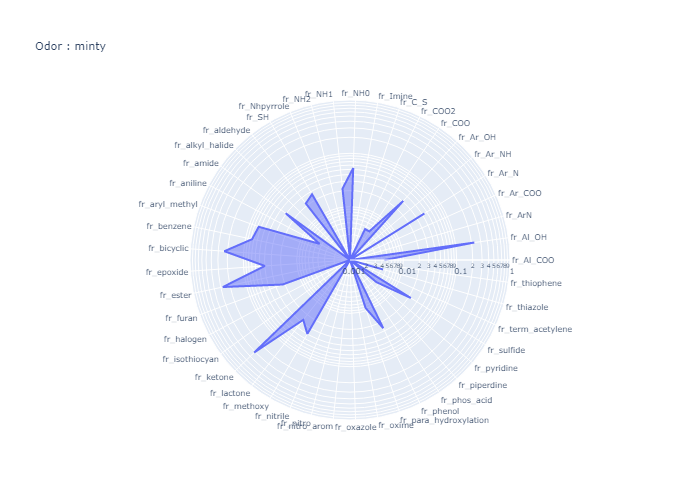

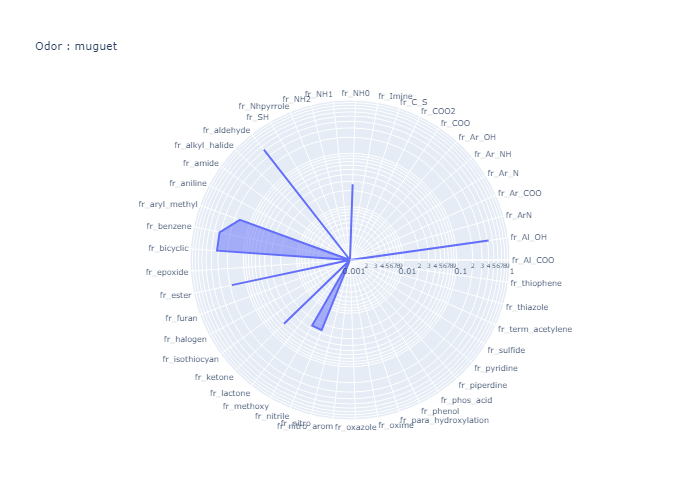


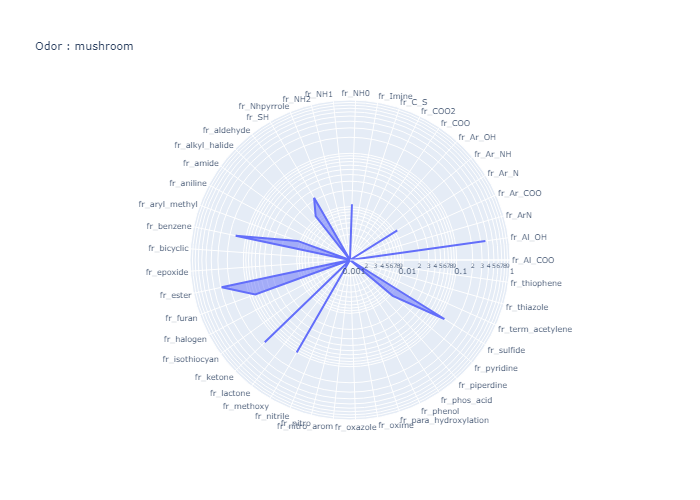

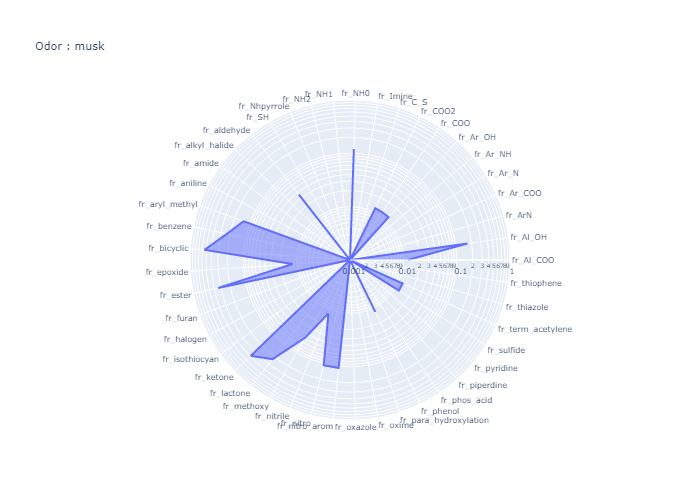

Supplement: Supplementary file 1 — Supplementary Information 1. [file 41598_2022_23176_MOESM1_ESM.docx]
