# Supplementary material for: Application of artificial intelligence to decode the relationships between smell, olfactory receptors and small molecules
Source: Sci Rep. 2022 Nov 5;12:18817. doi: 10.1038/s41598-022-23176-y (PMC9637086; doi:10.1038/s41598-022-23176-y)

**Figure S2**: Radar plots based on the frequency of 64 physicochemical properties observed on the set compounds associated to an olfactory receptor. Each radar plot corresponds to one olfactory receptor.


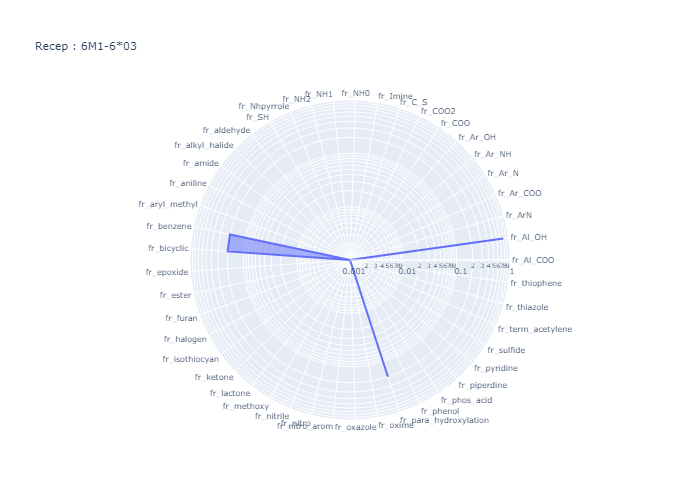

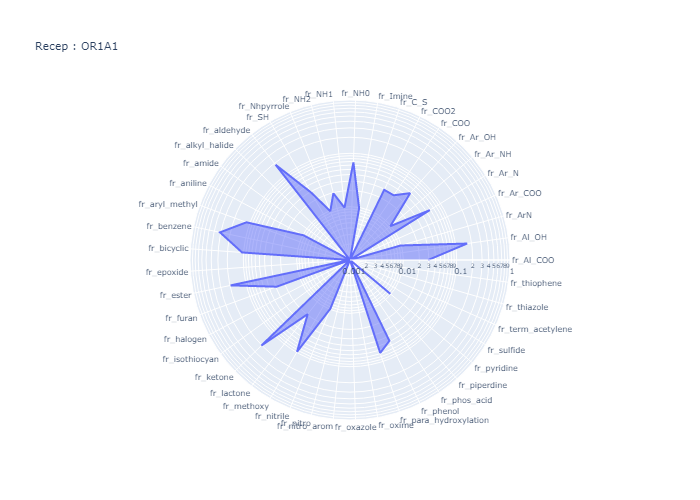


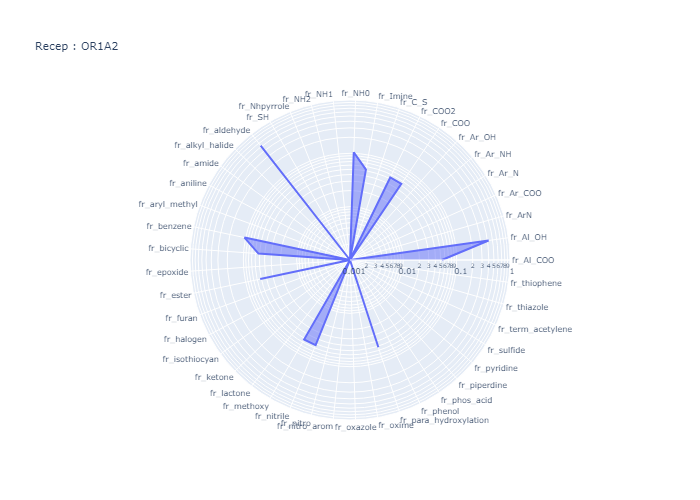

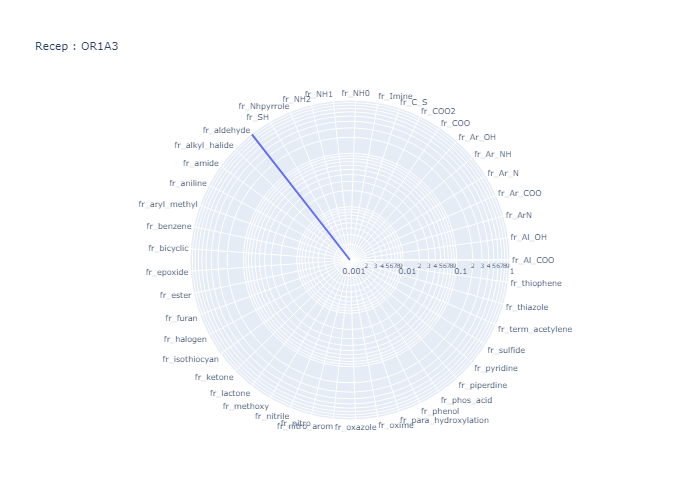


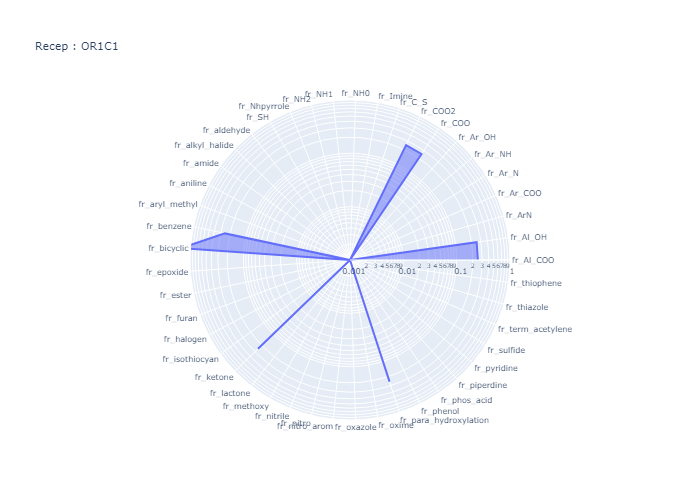

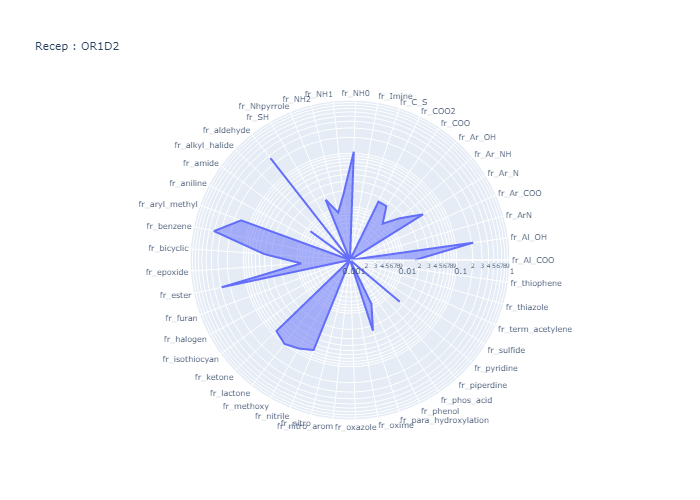


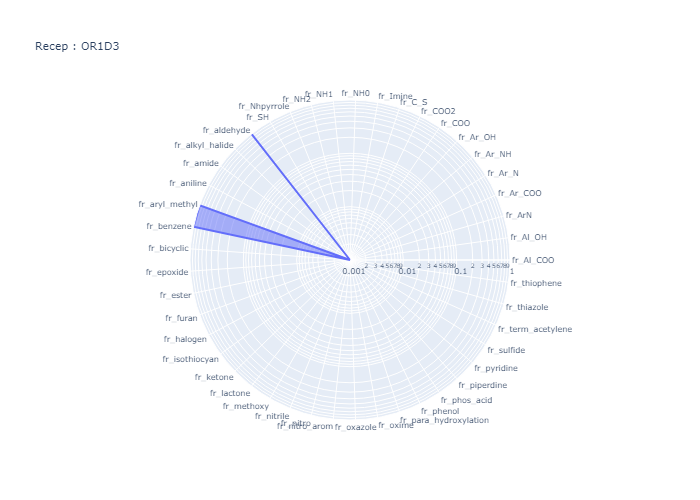

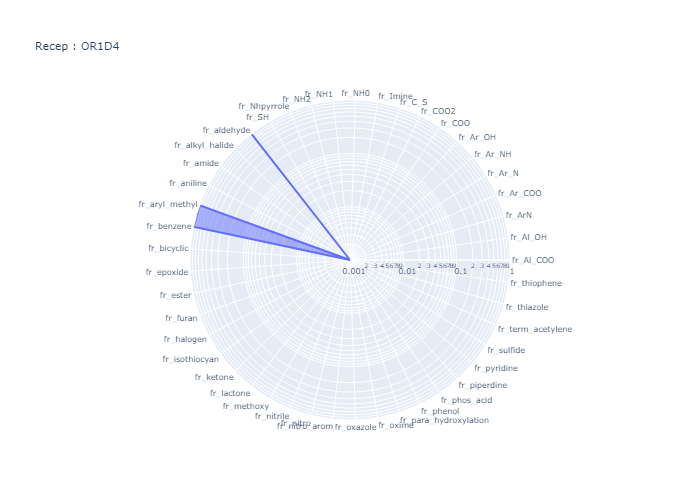


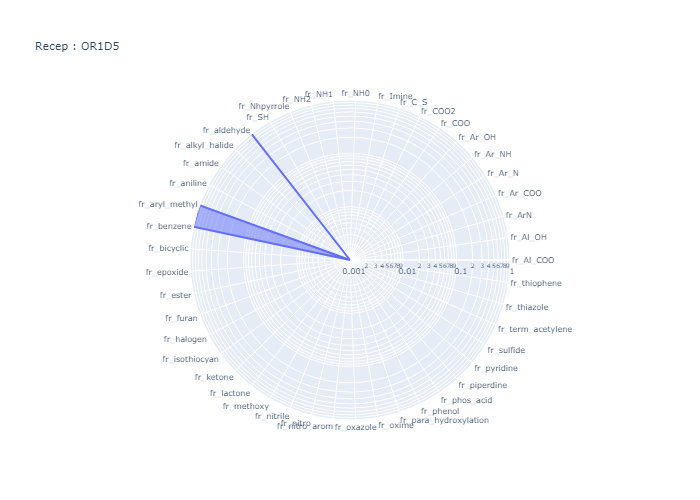

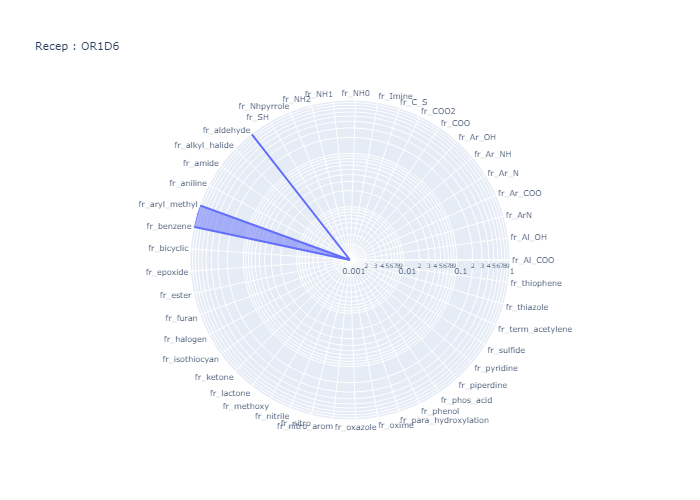


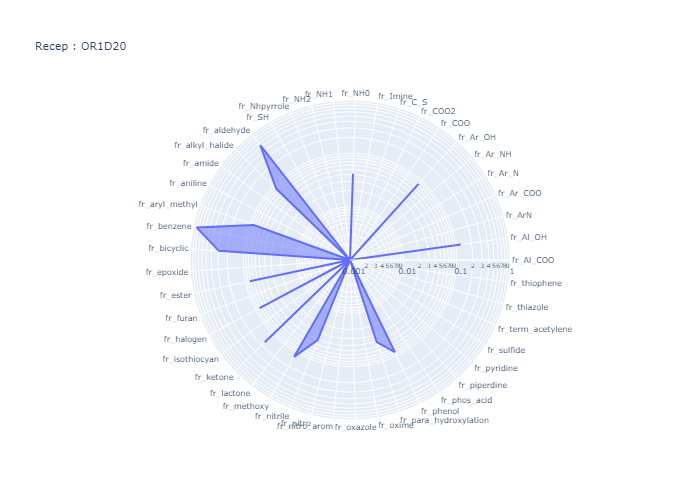

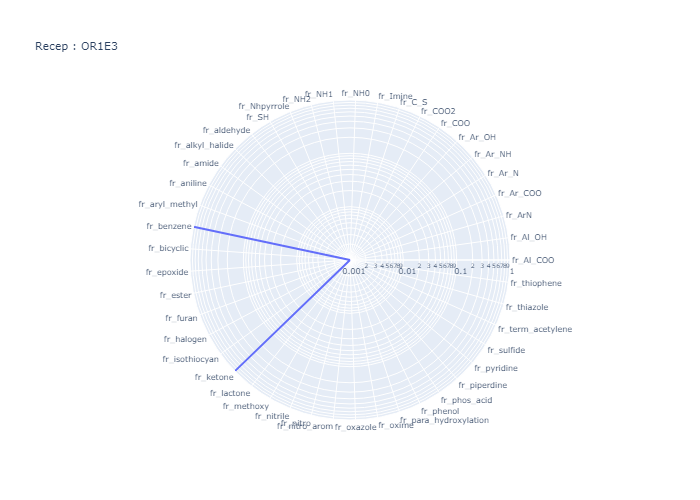


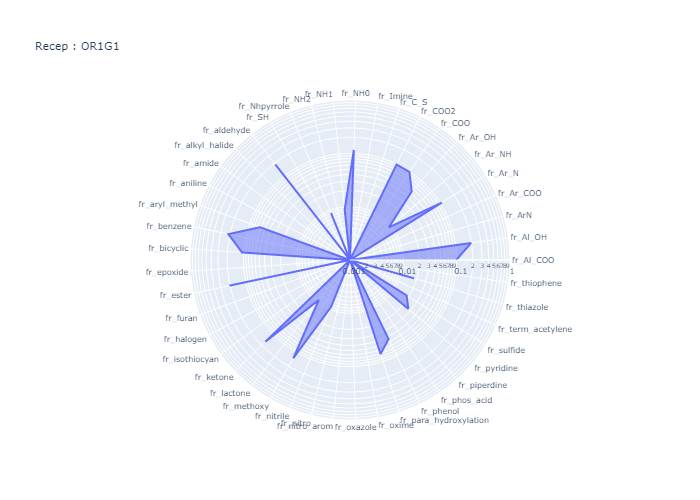

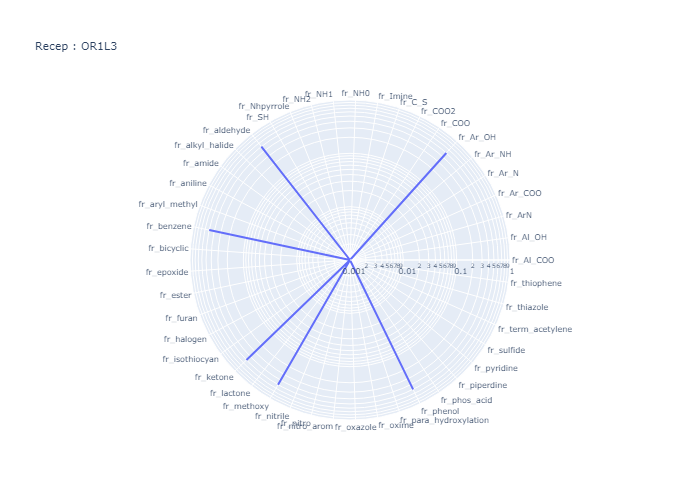


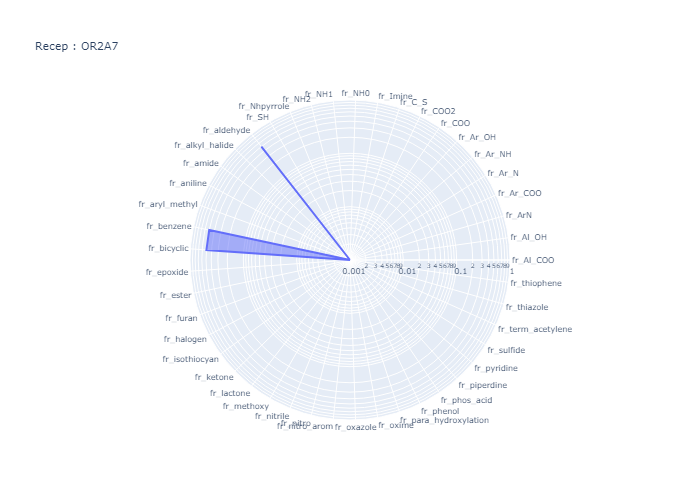

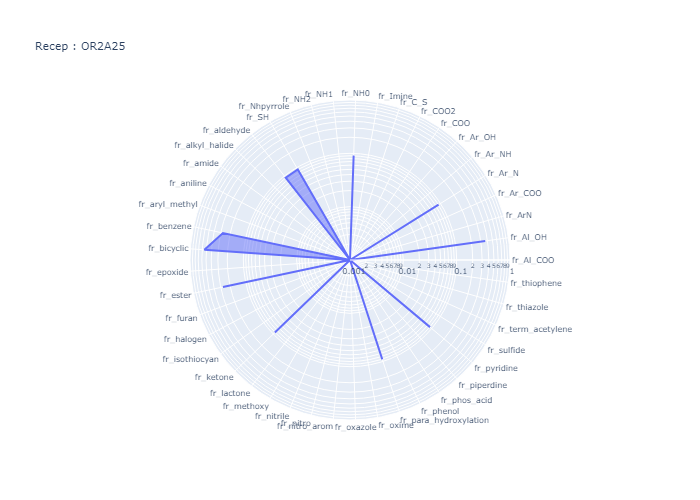


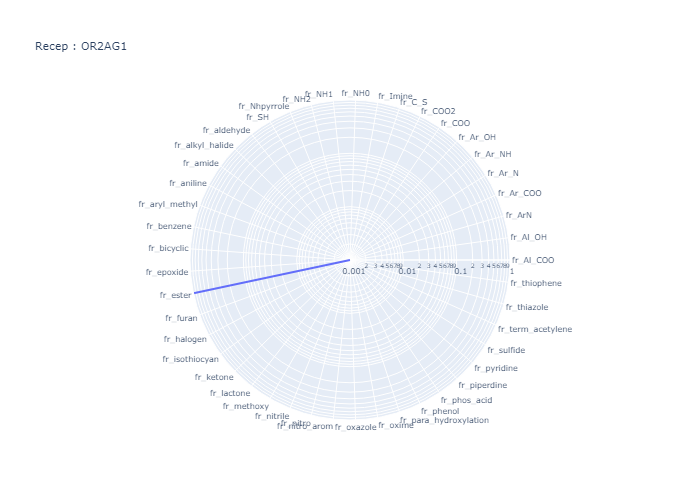

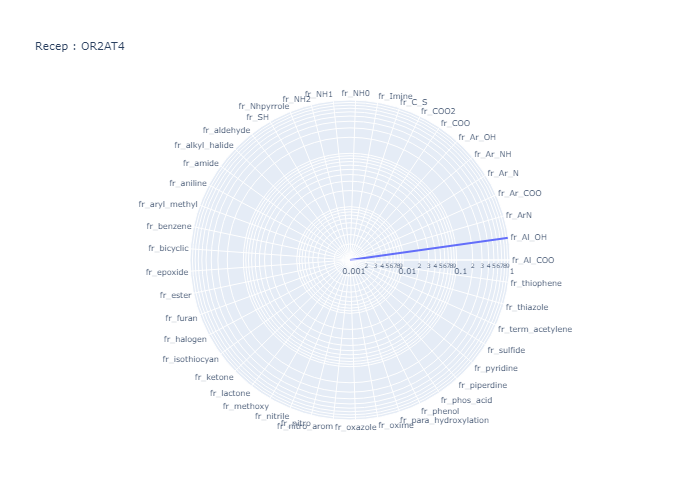


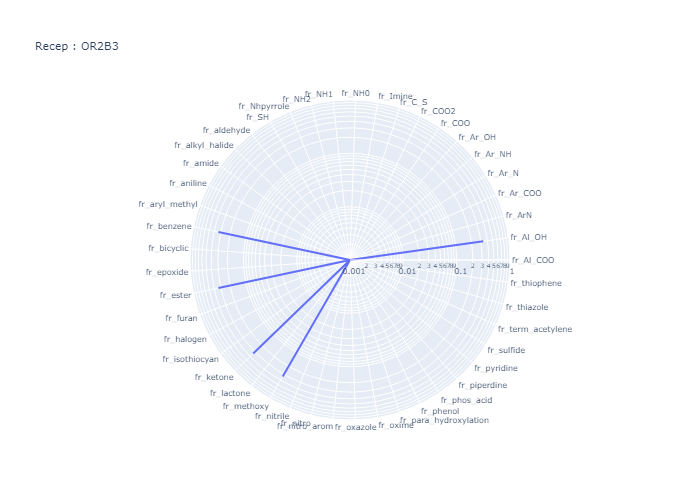

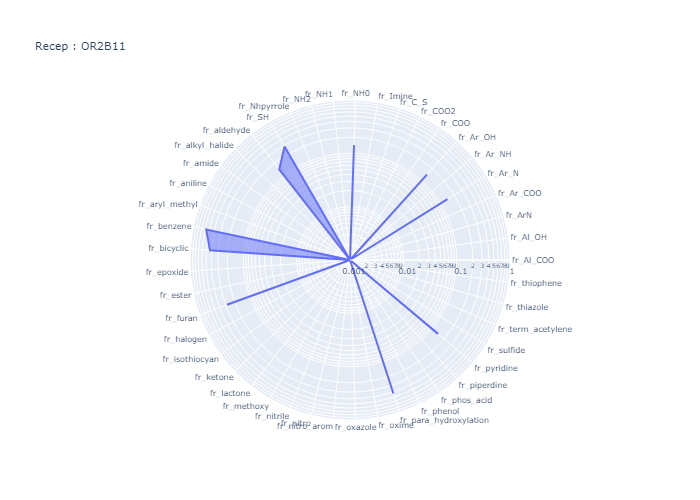


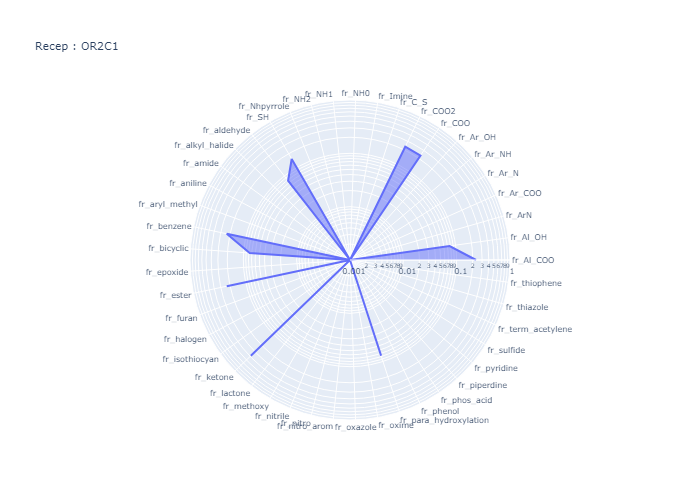

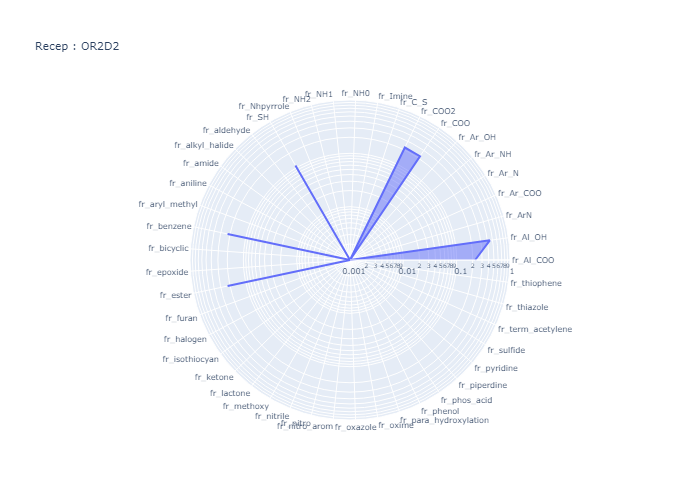


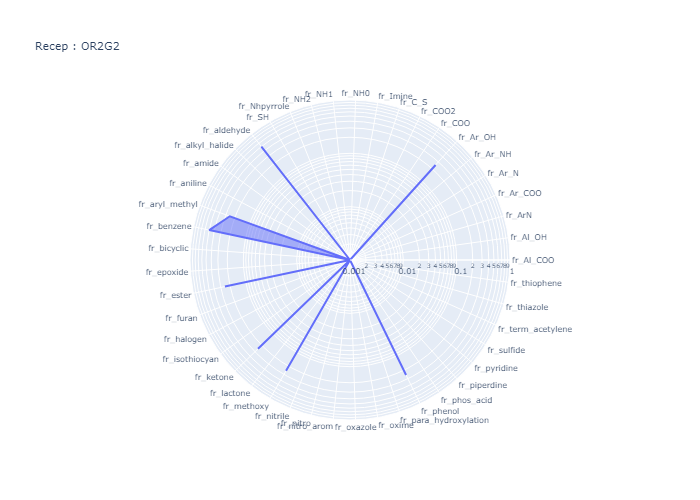

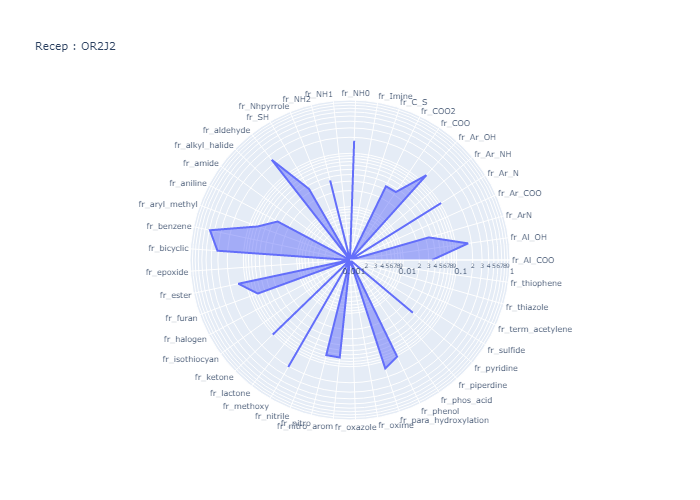


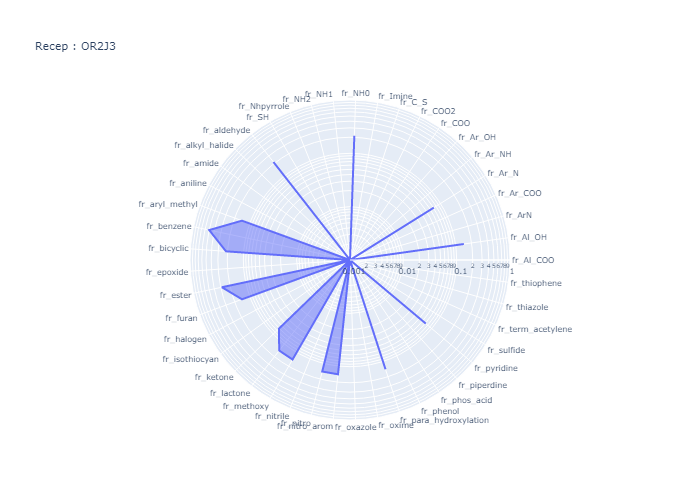

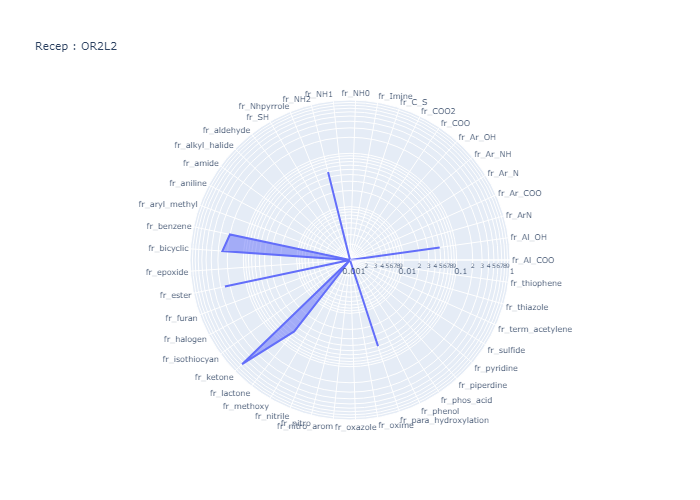


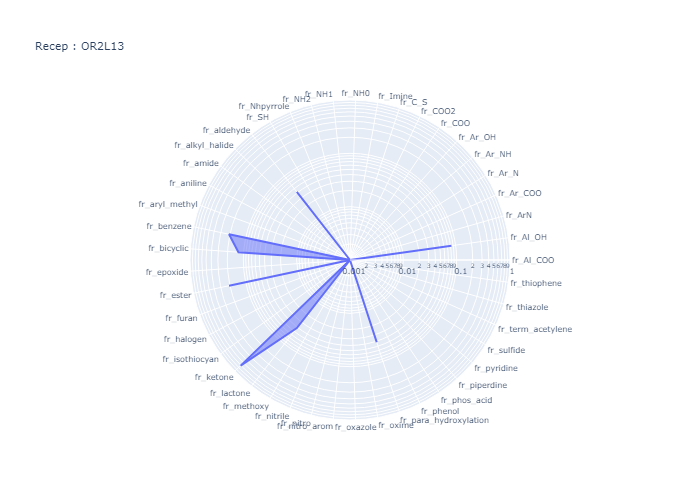

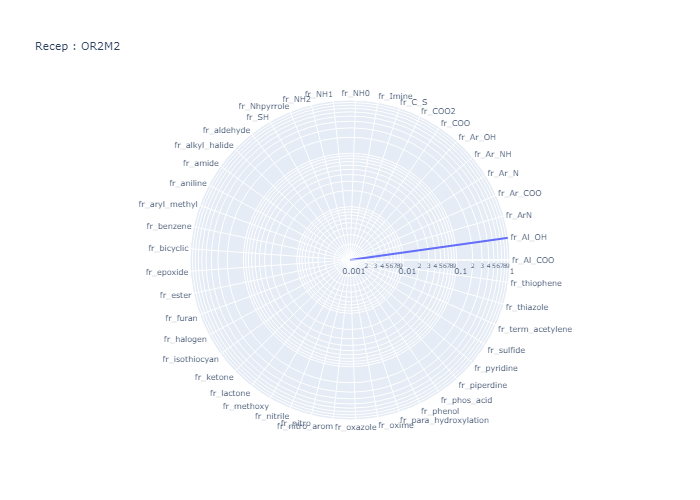


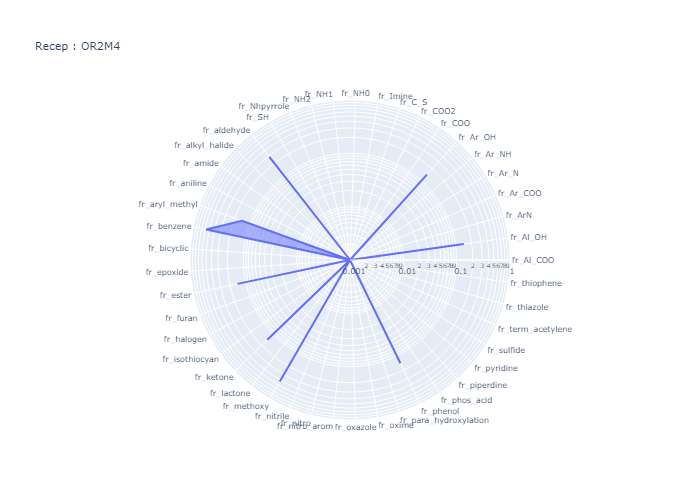

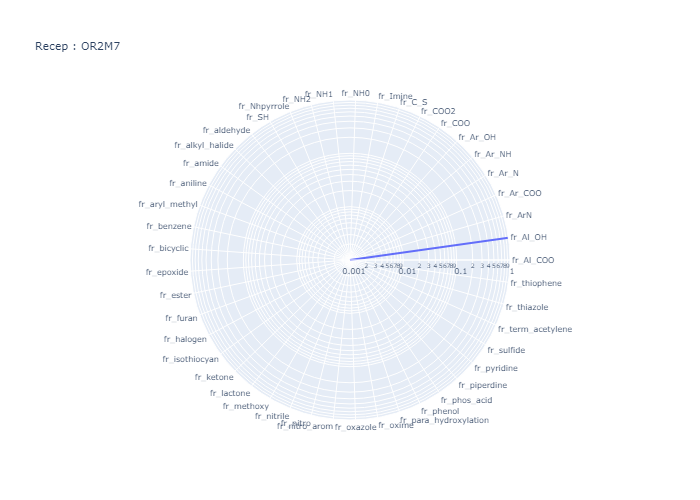


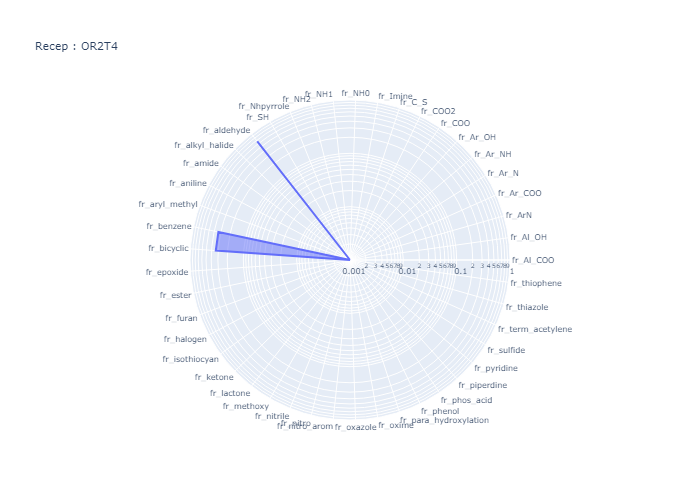

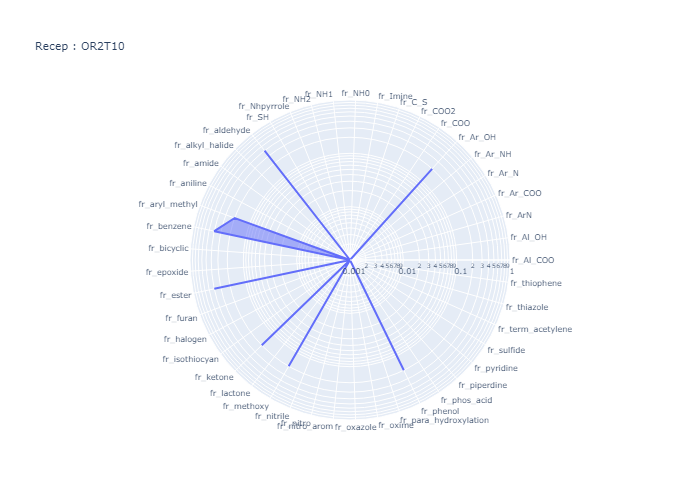


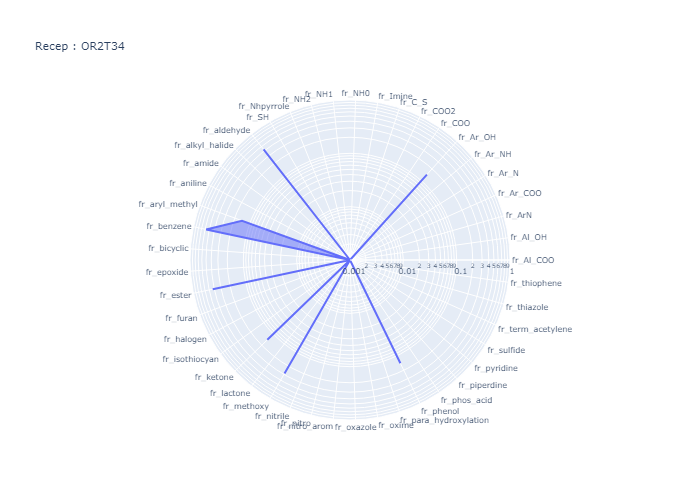

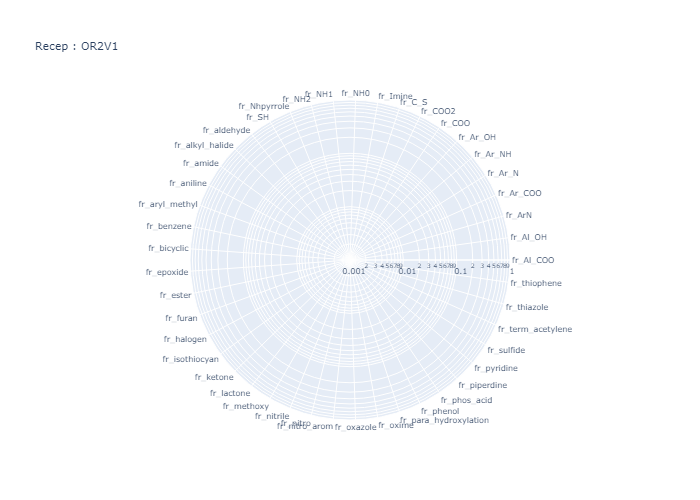


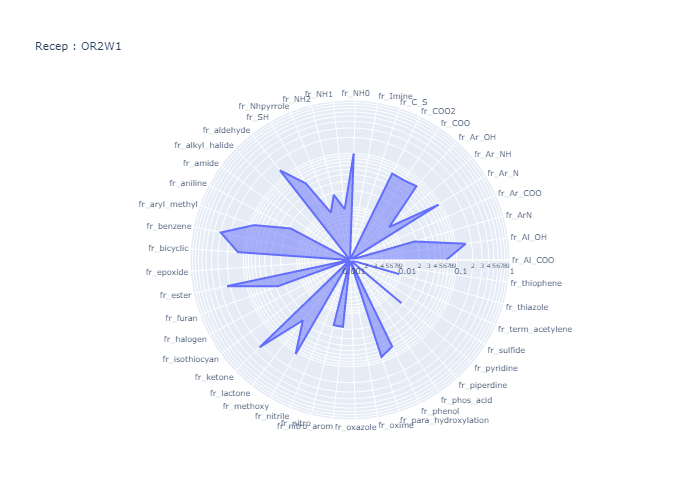

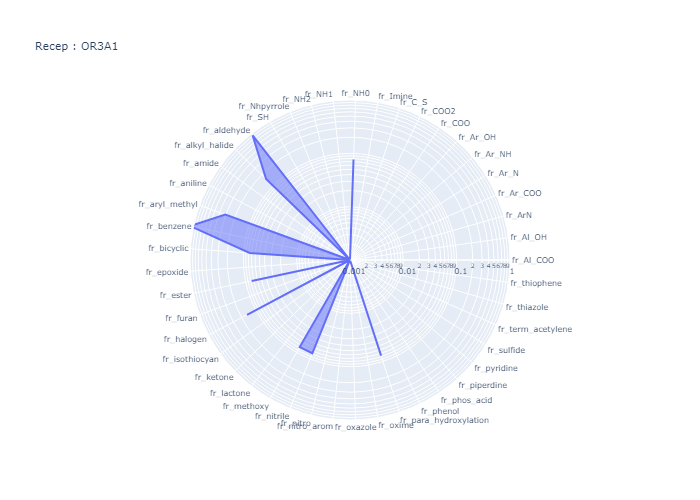


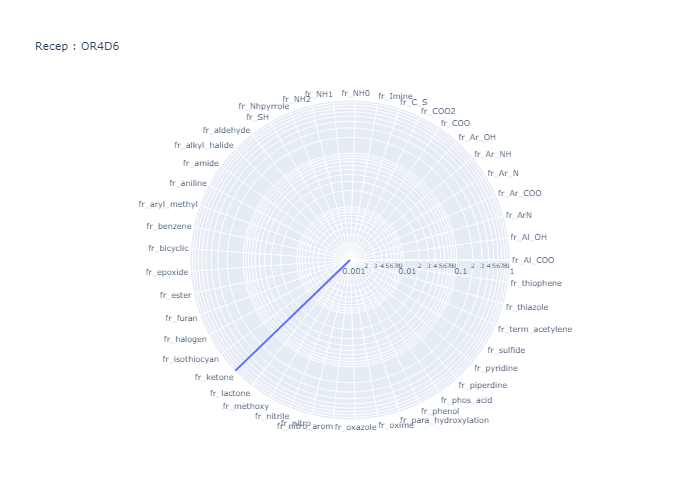

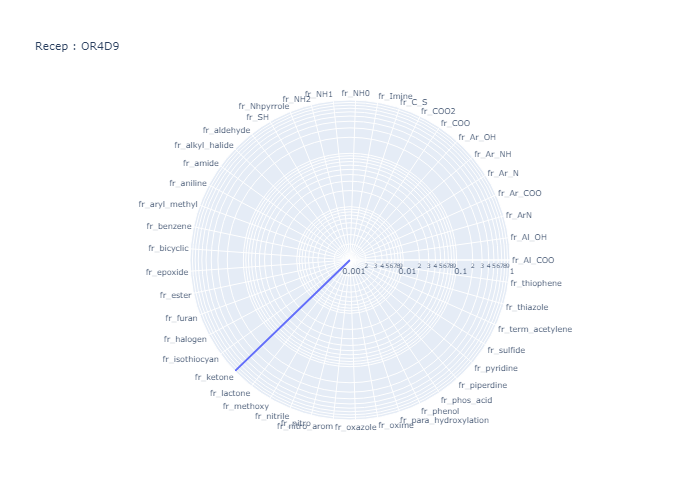


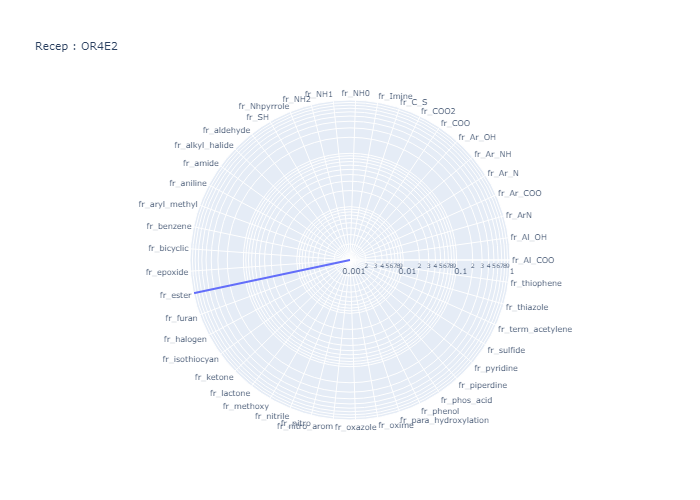

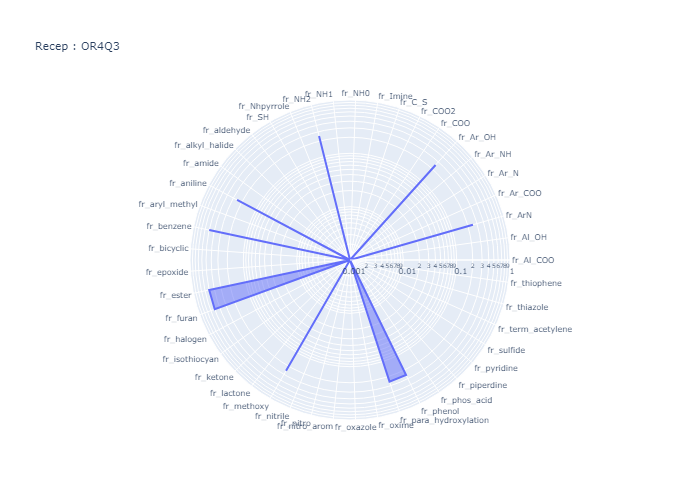


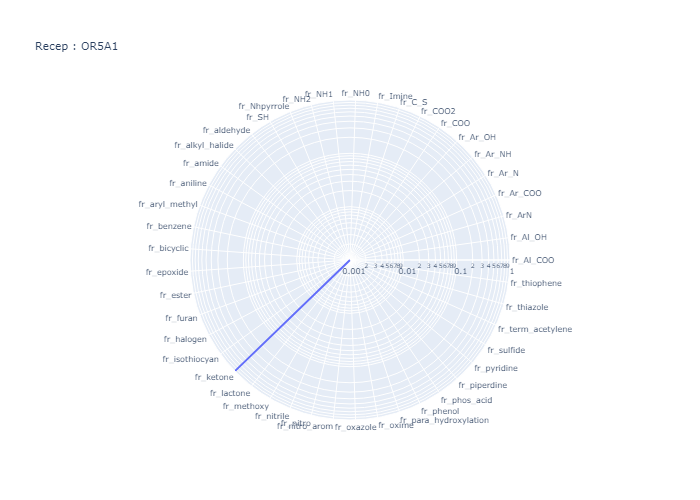

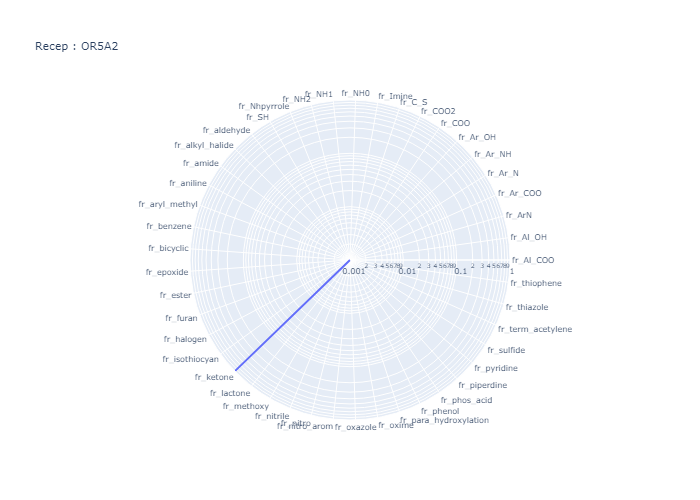


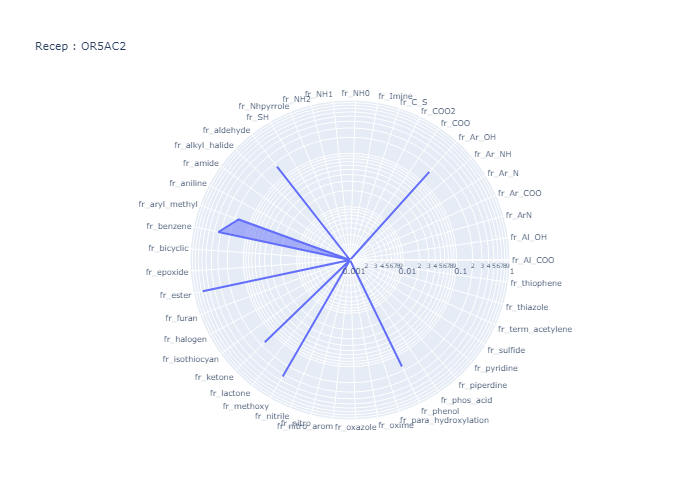

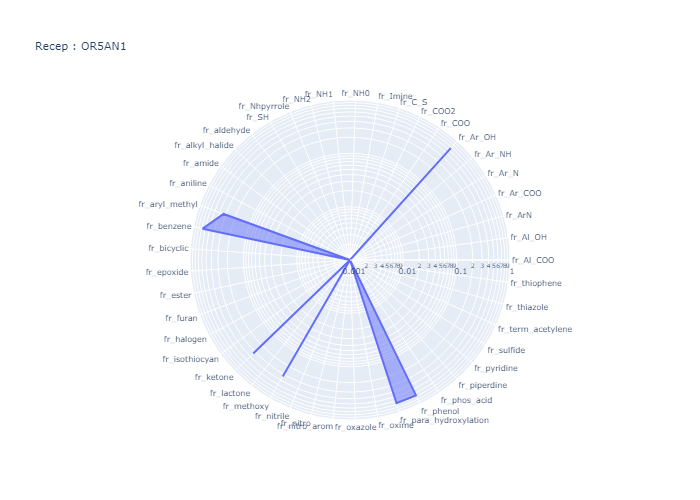


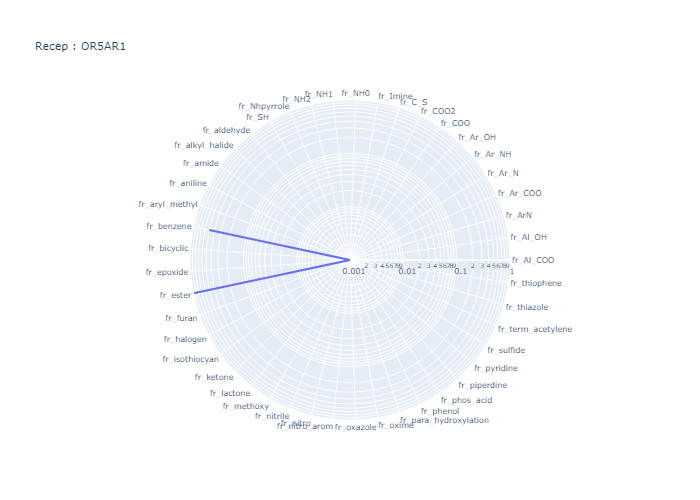

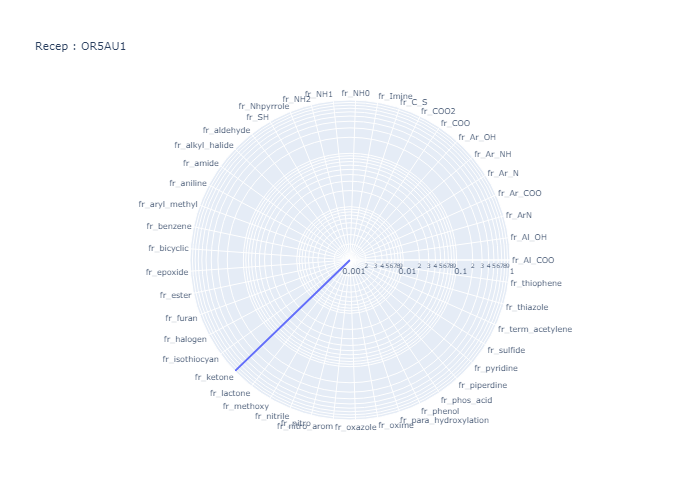


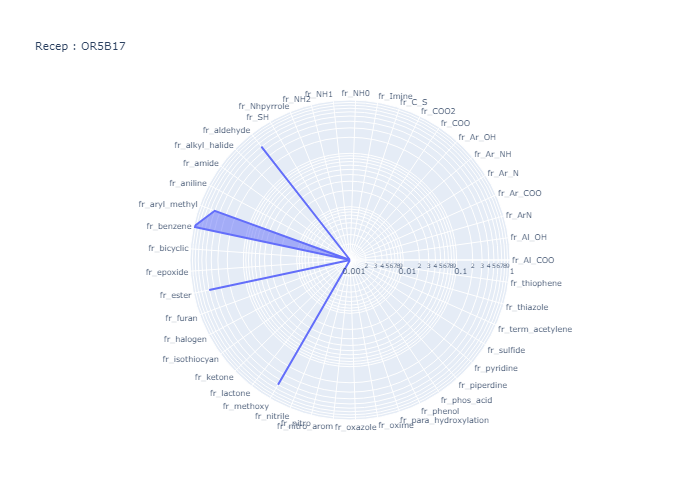

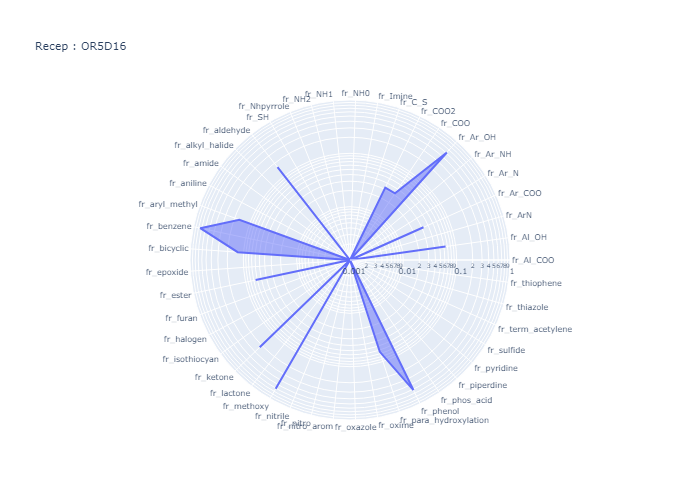


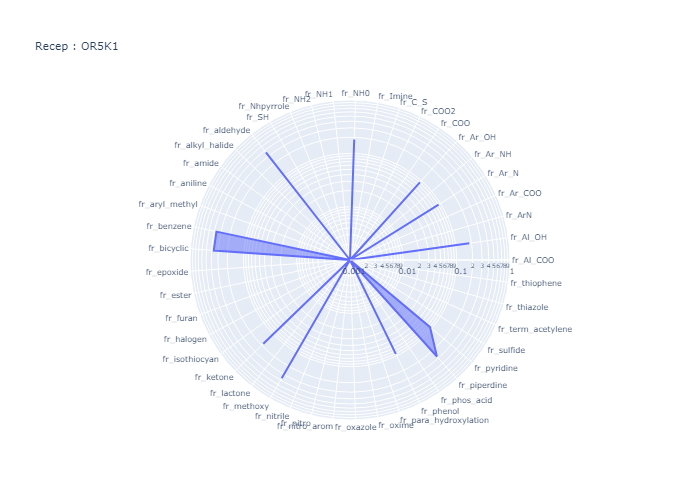

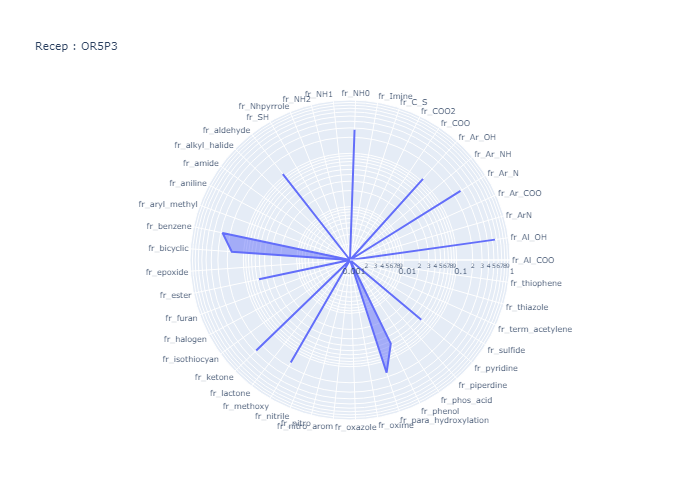


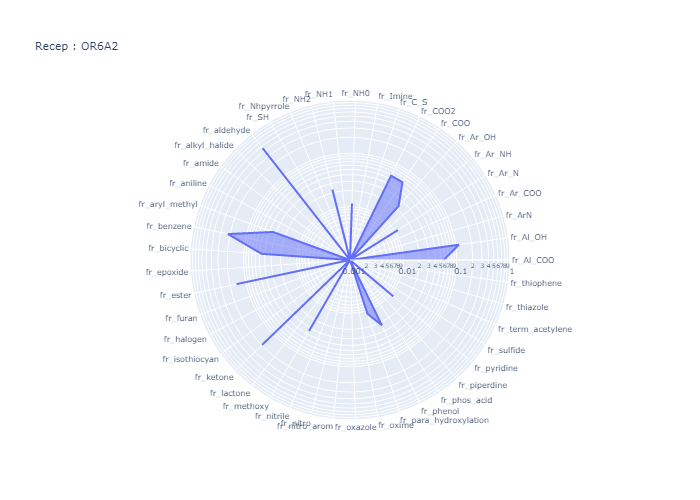

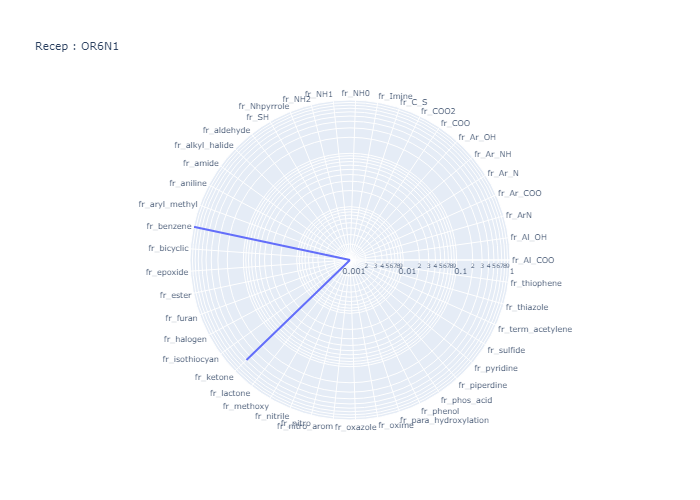


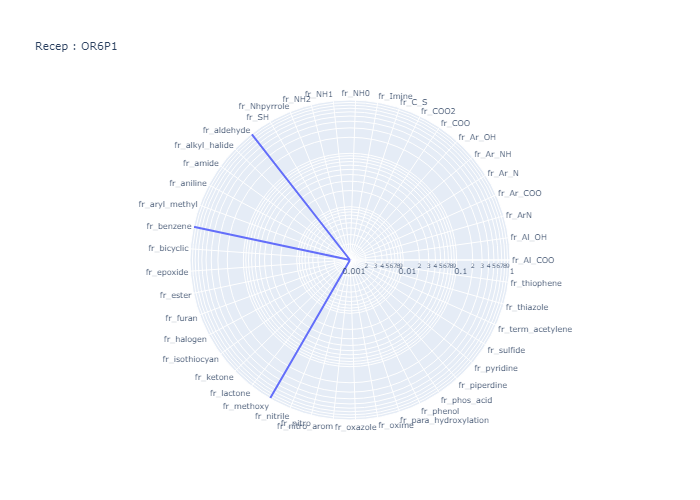

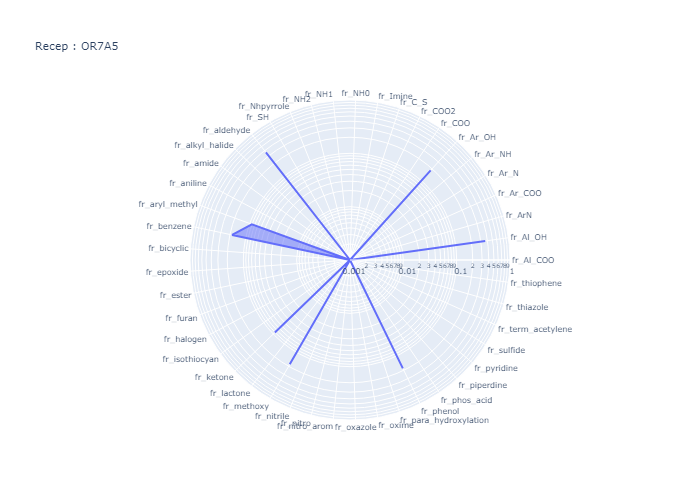


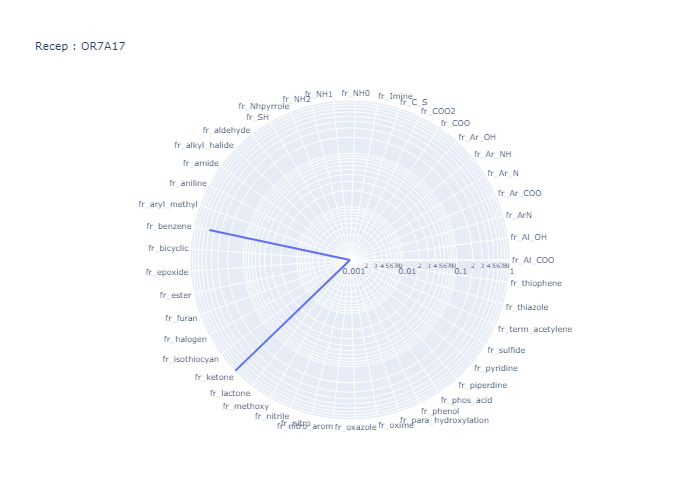

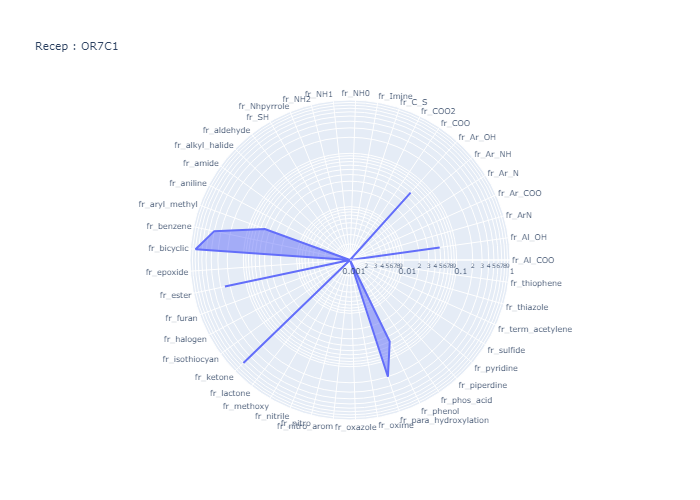


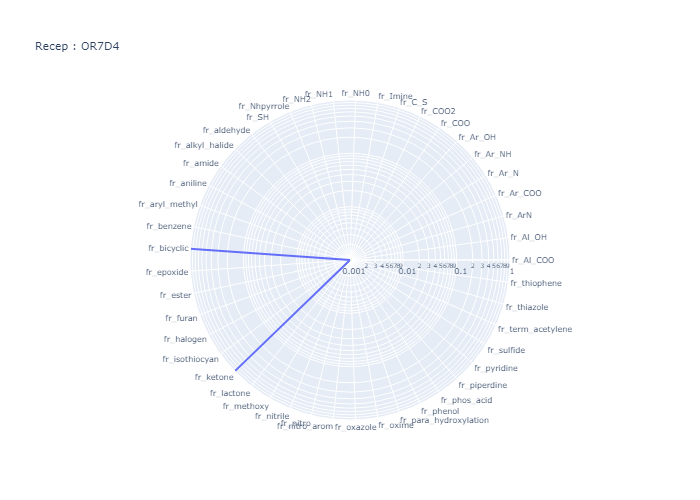

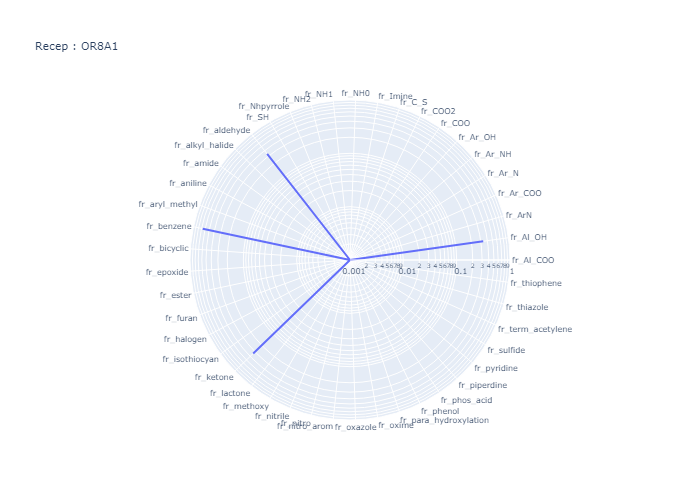


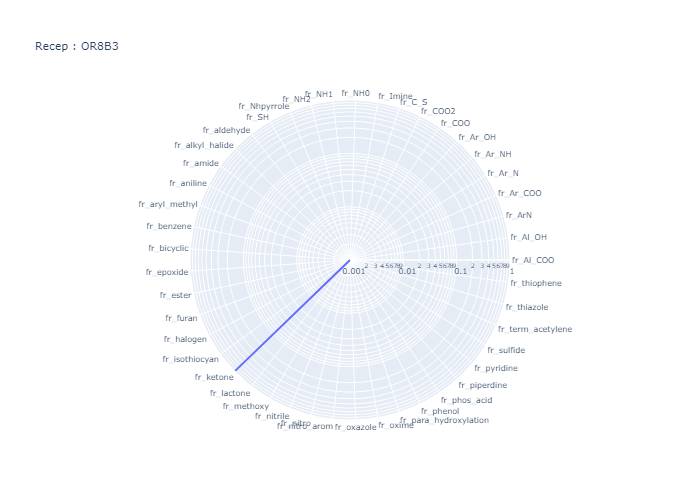

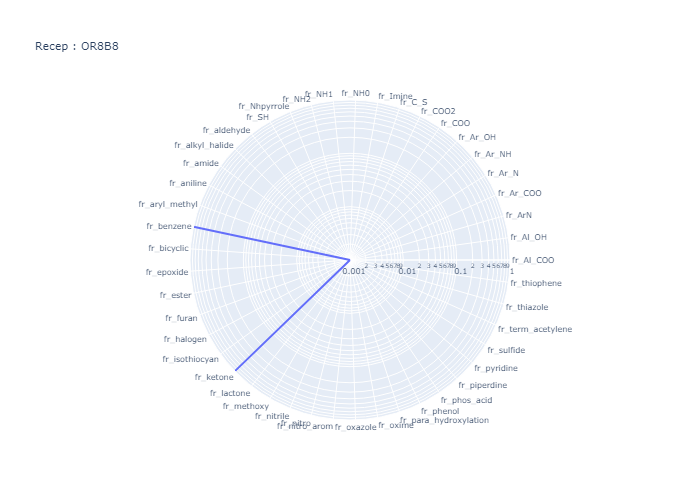


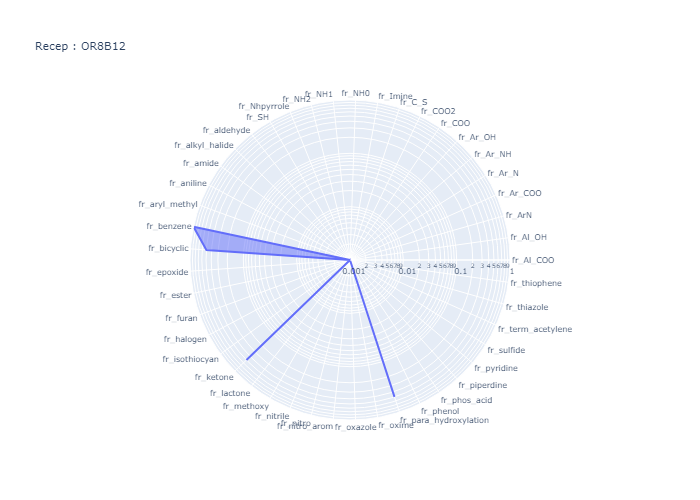

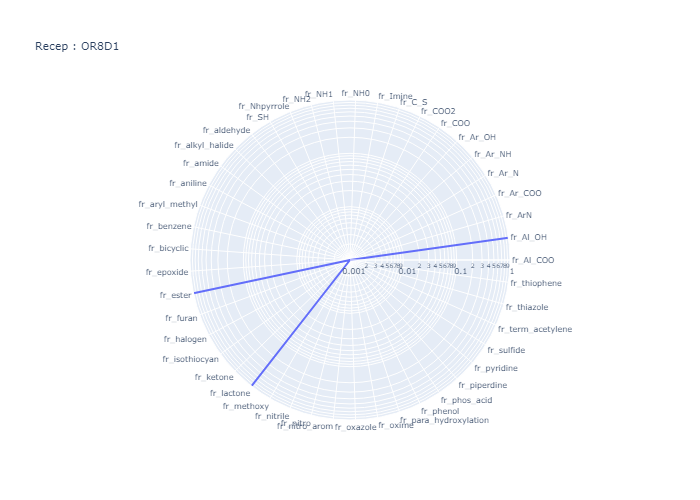


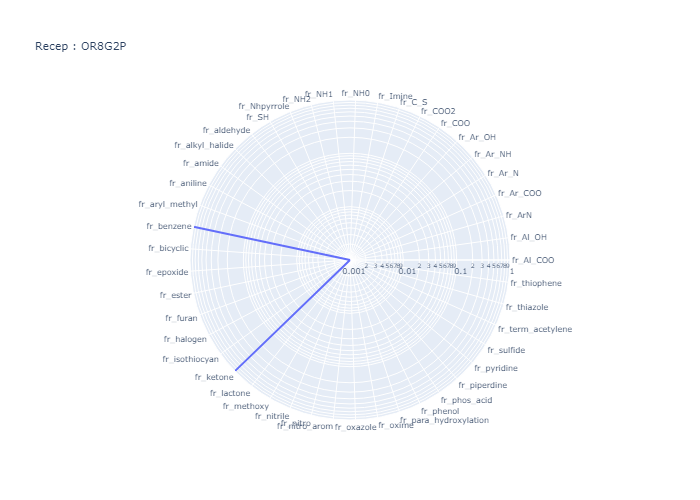

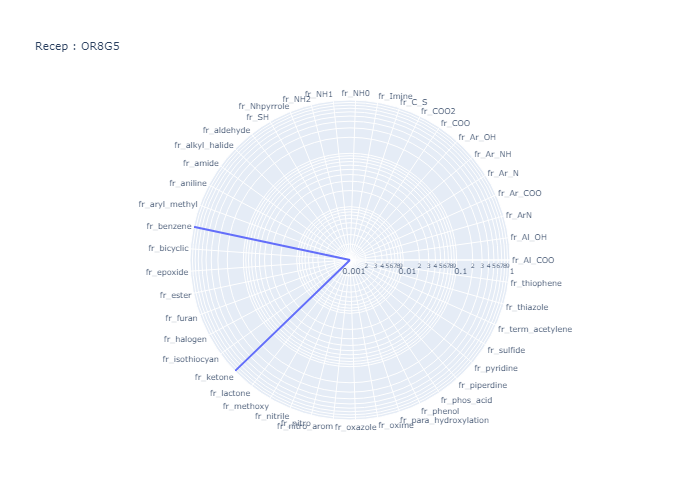


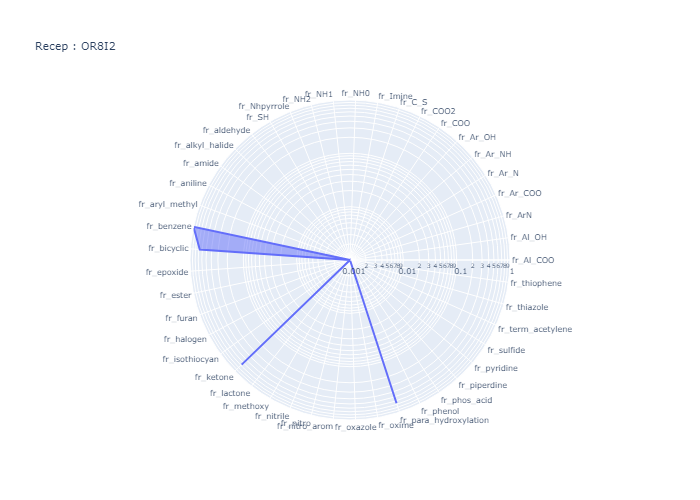

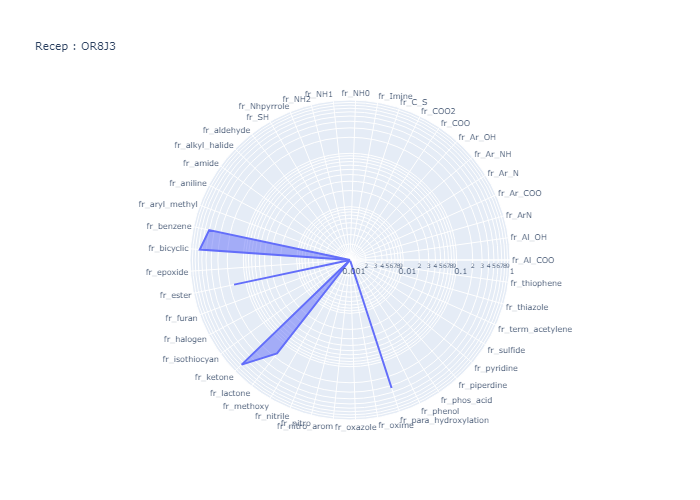


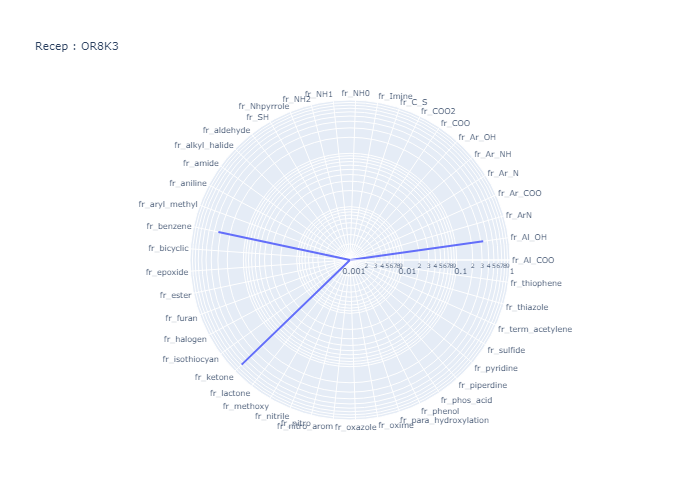

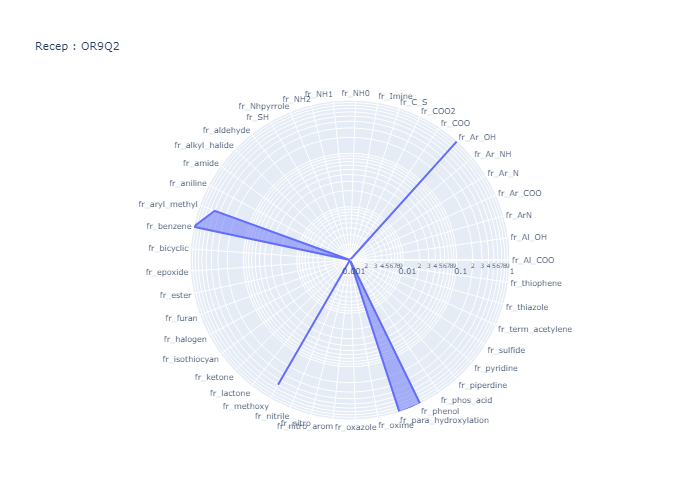


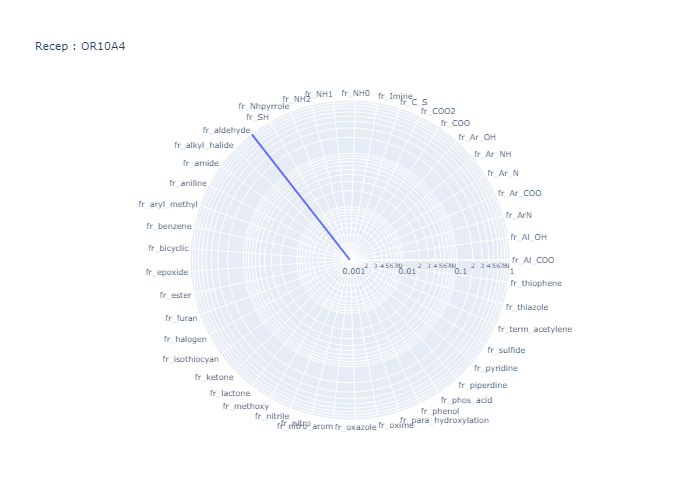

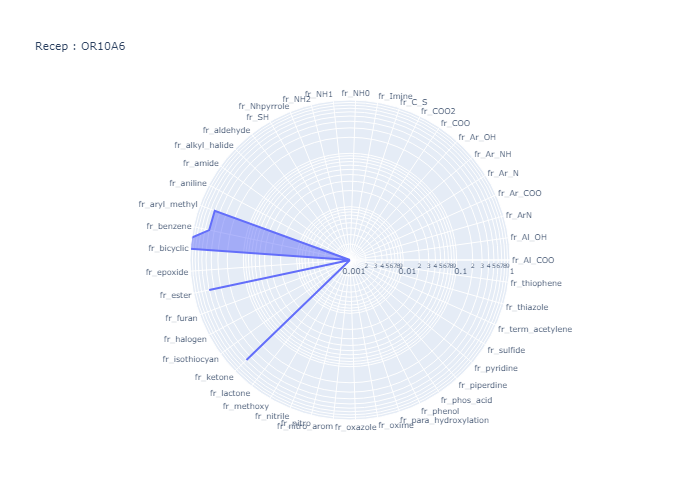


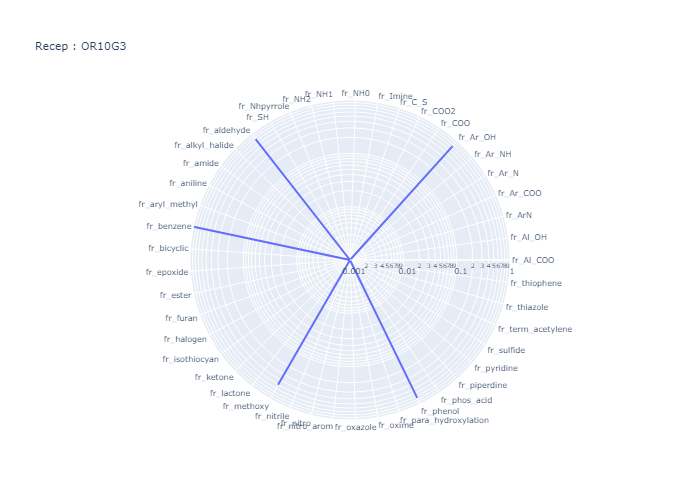

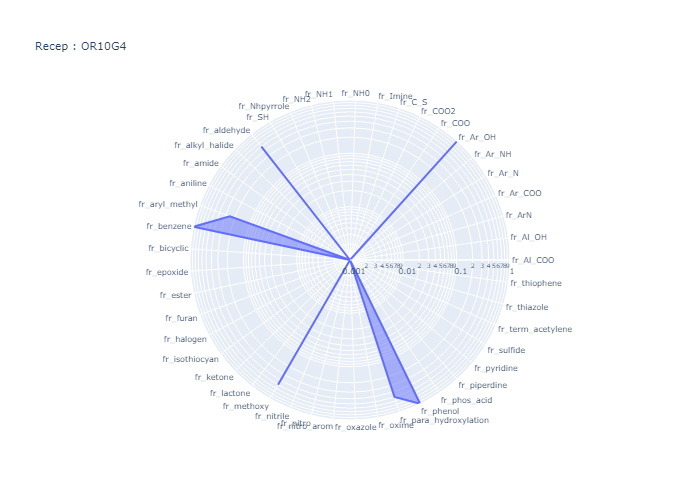


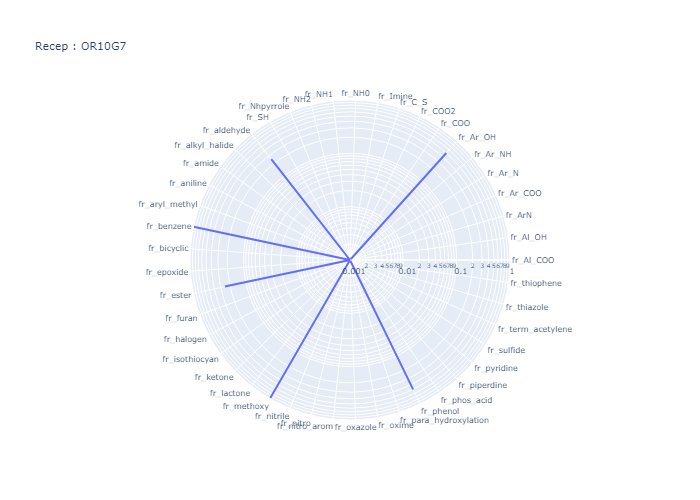

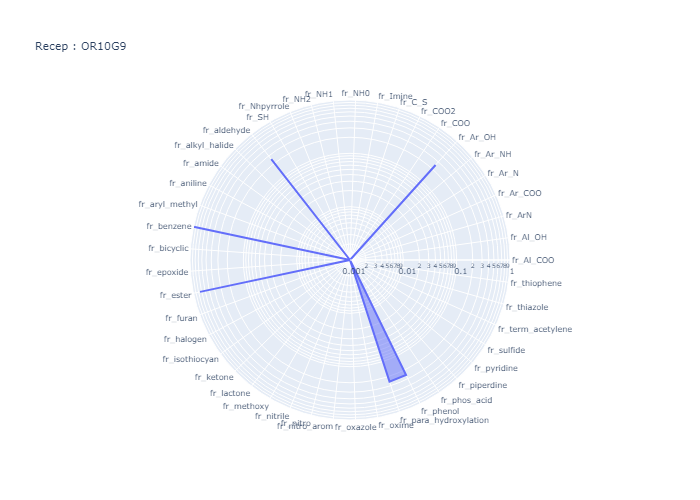


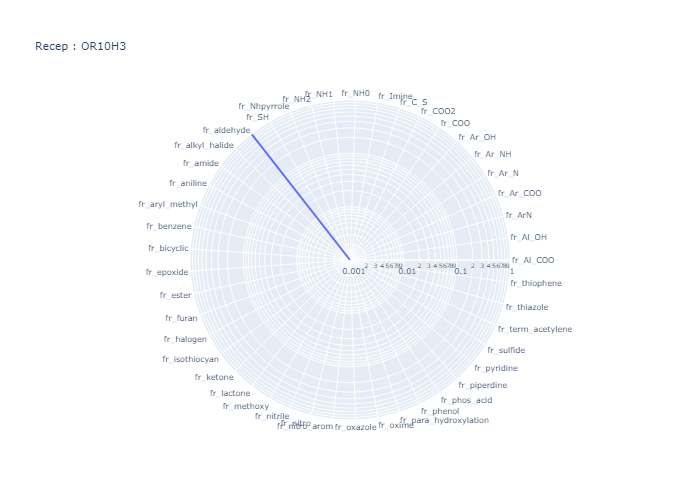

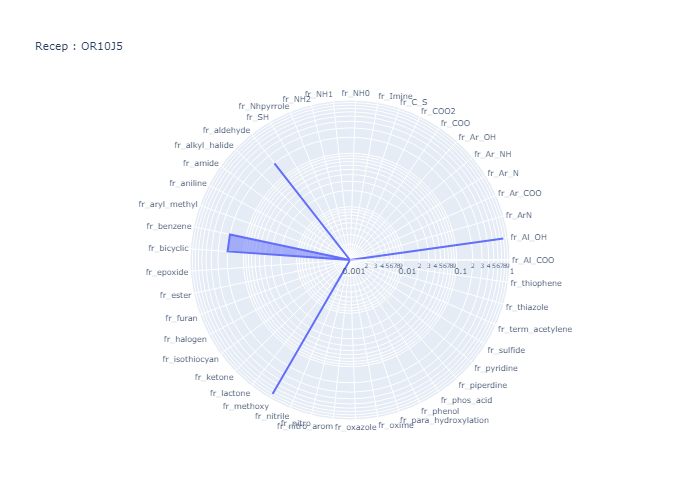


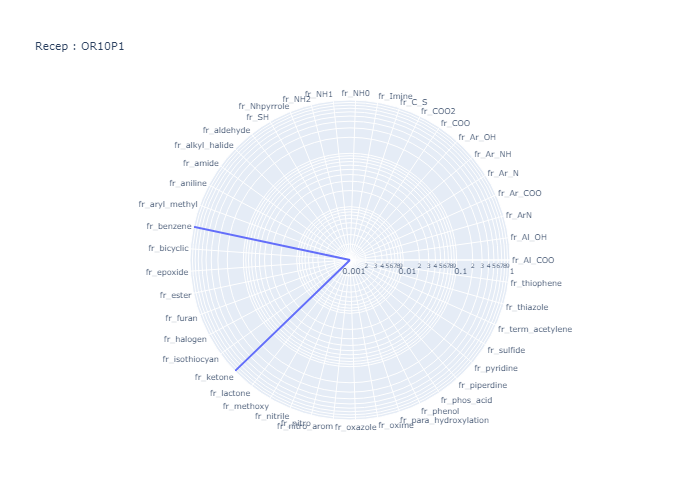

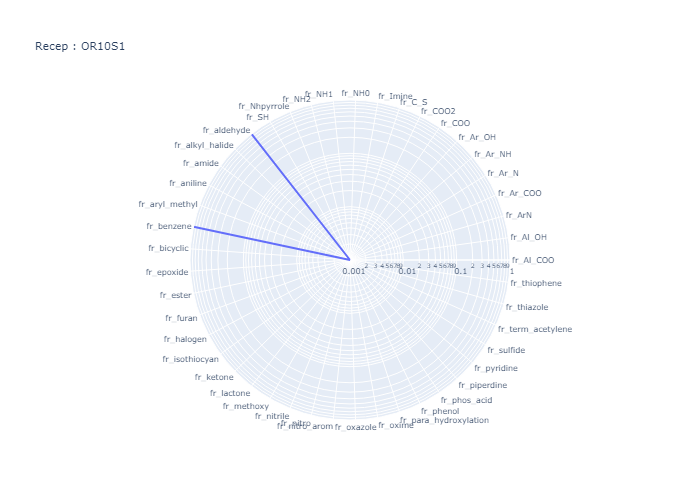


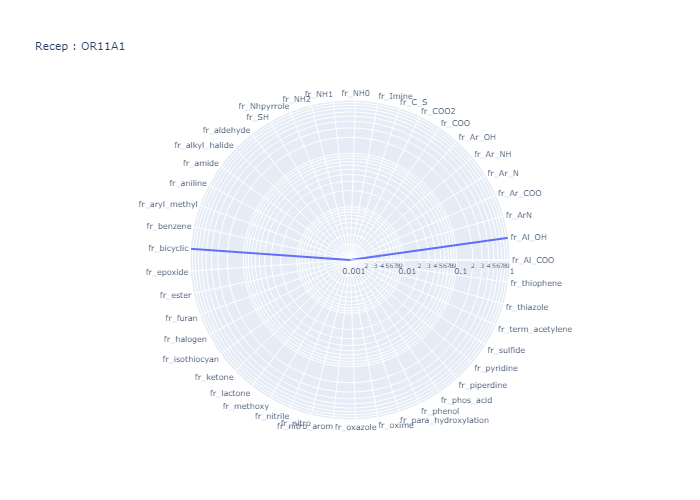

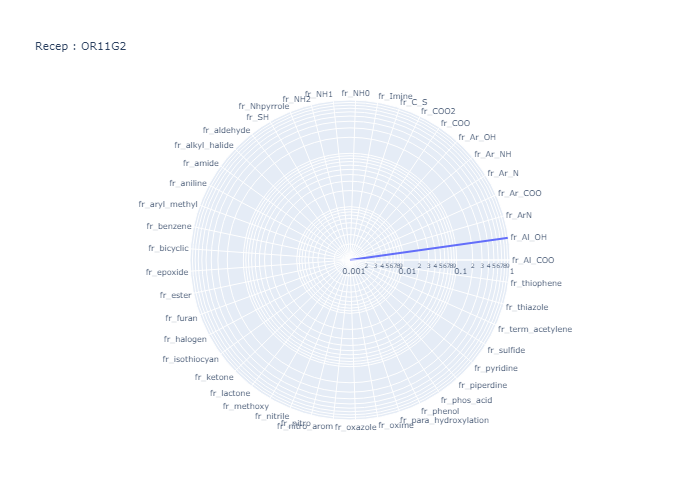


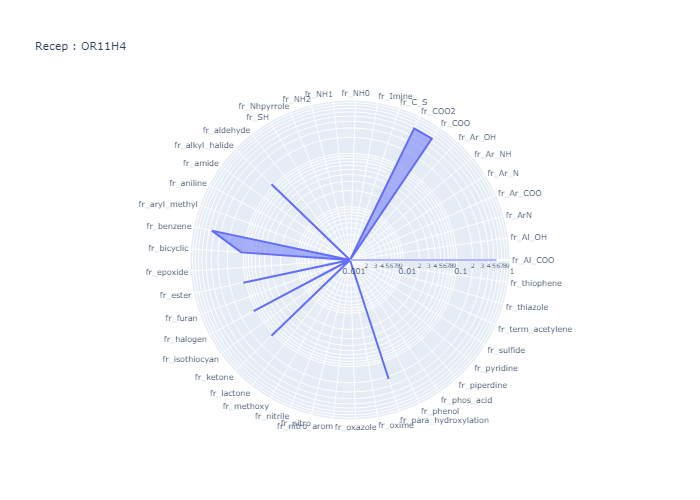

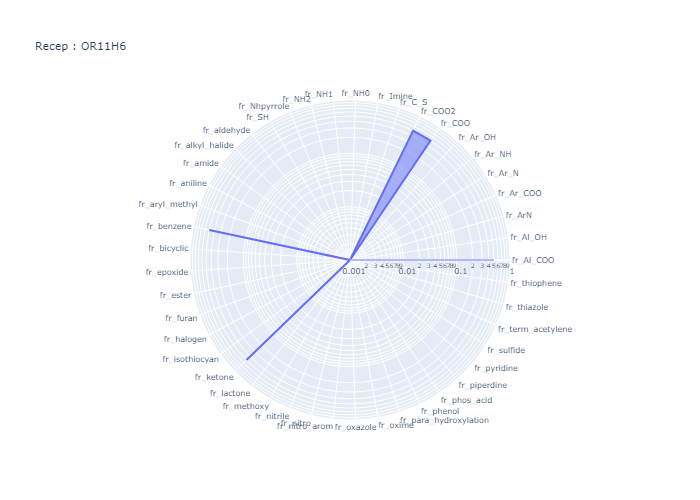


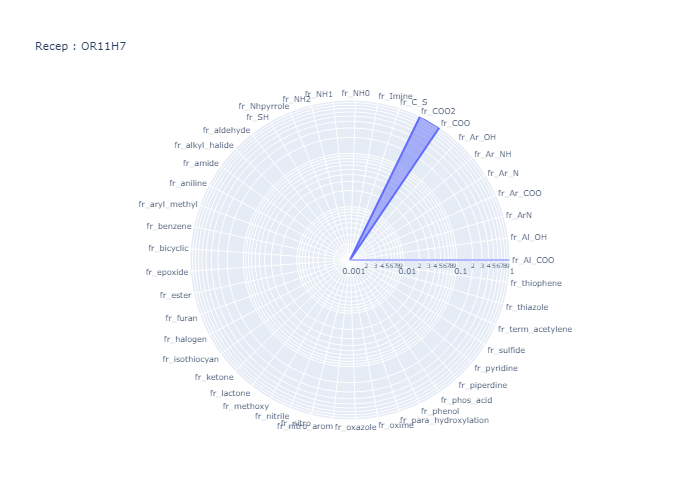

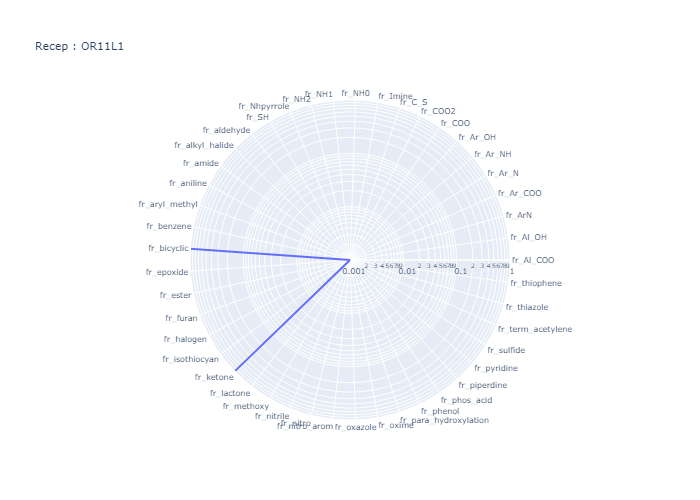


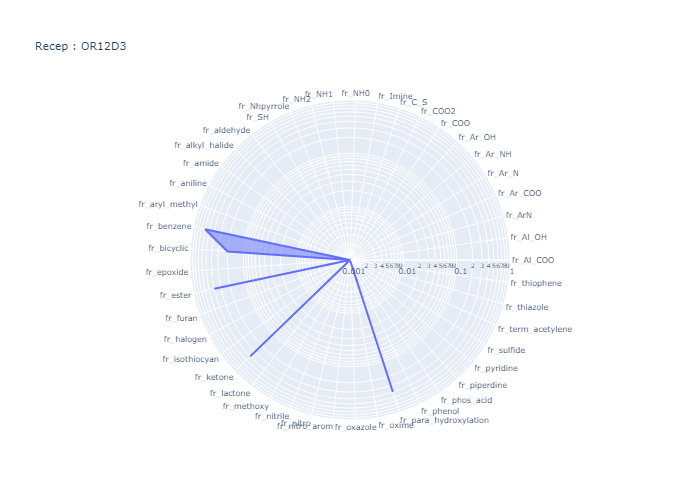

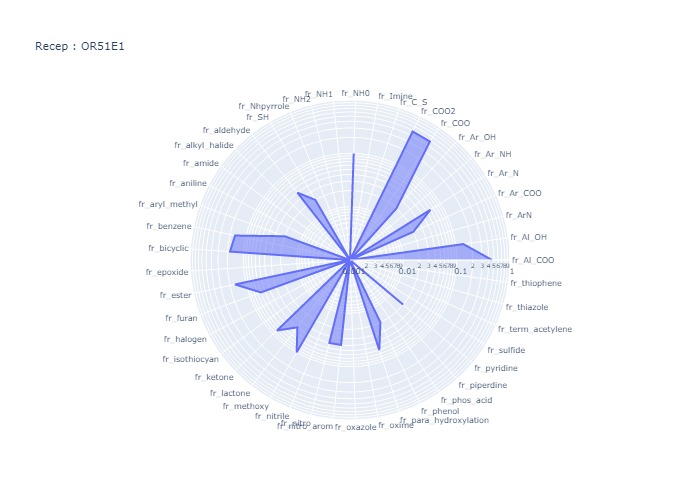


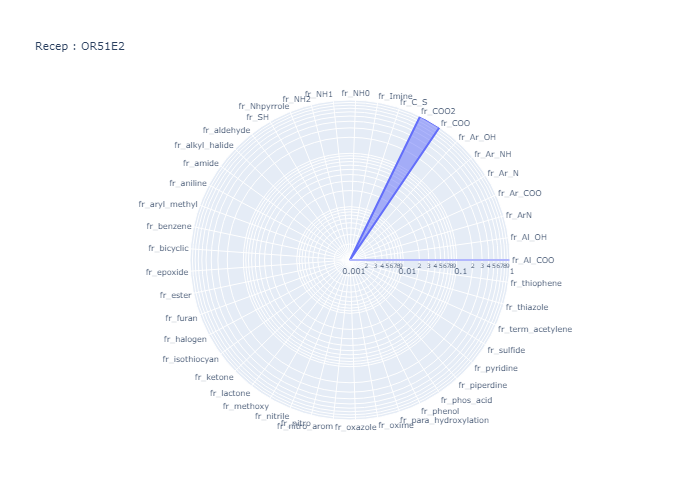

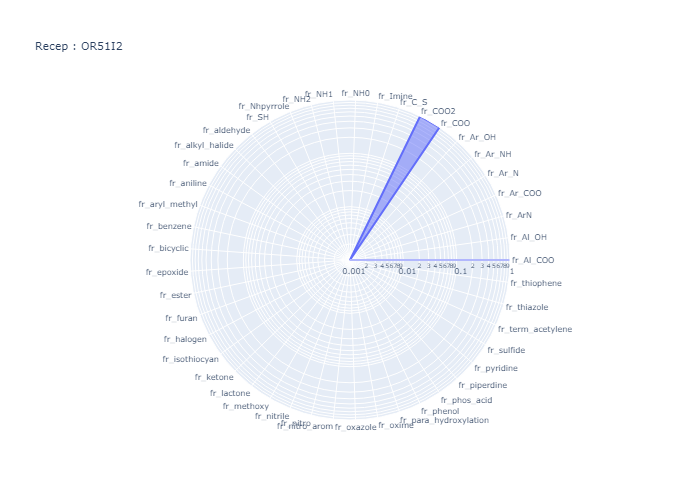


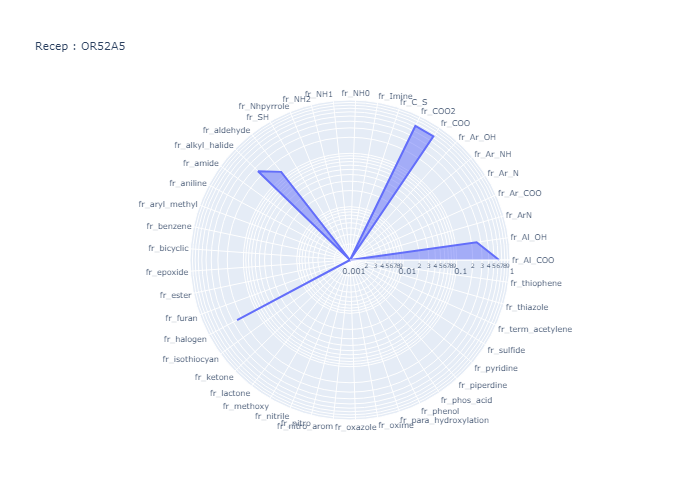

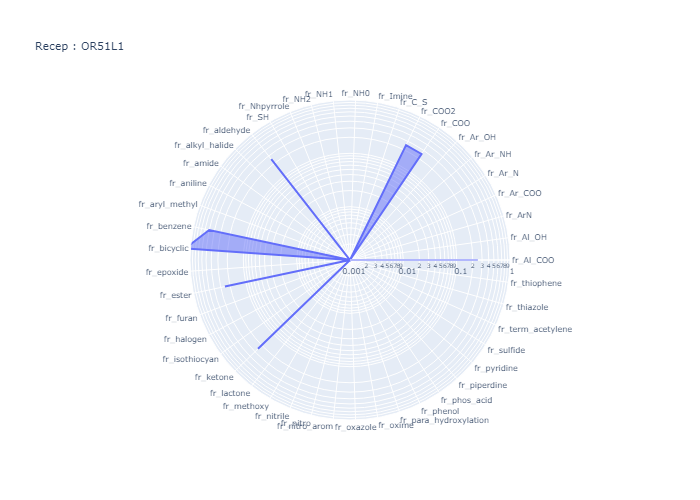


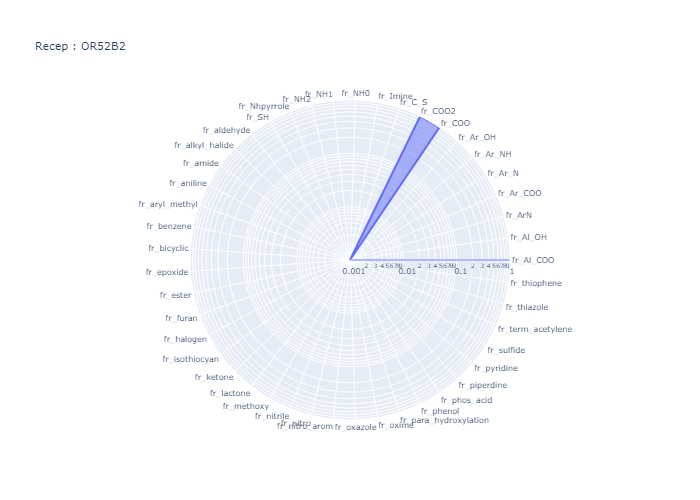

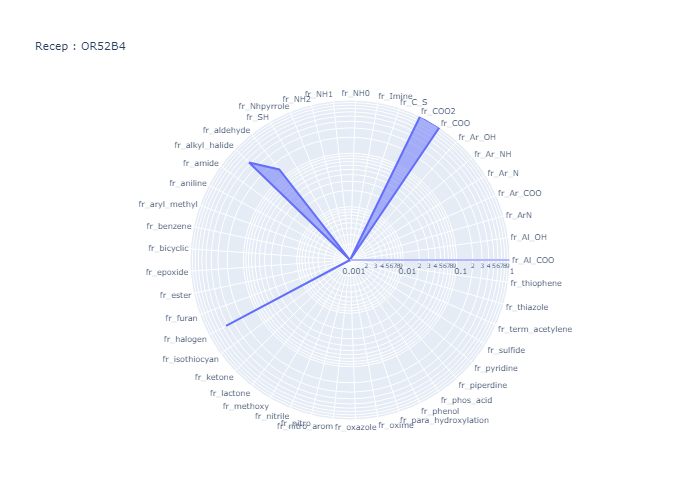


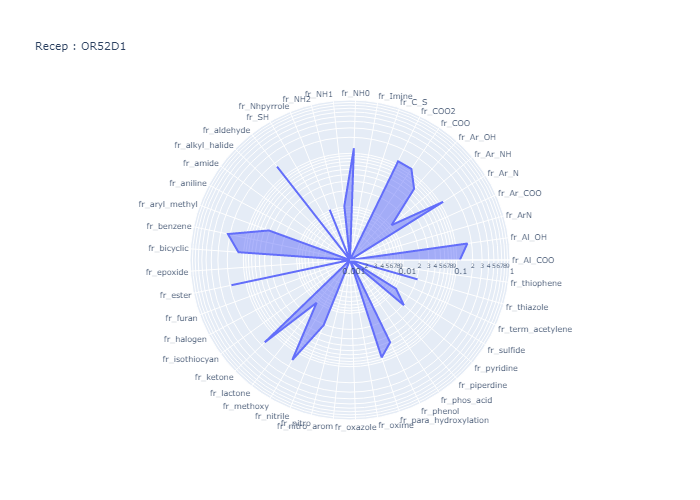

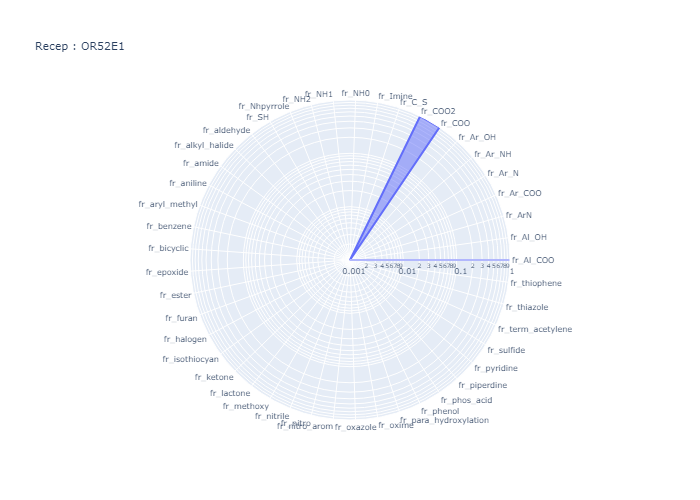


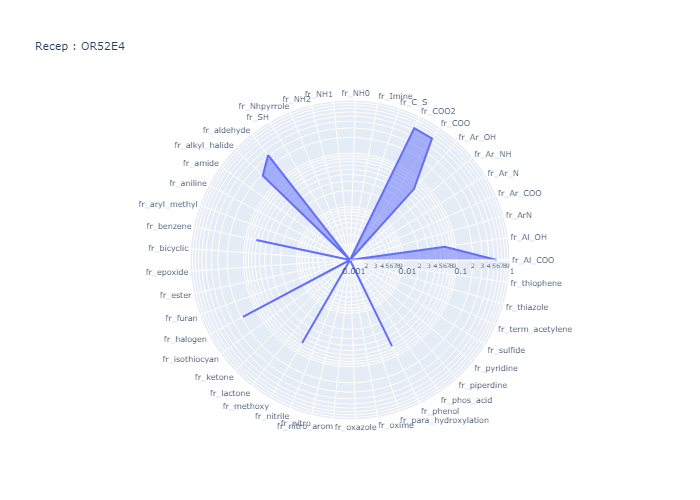

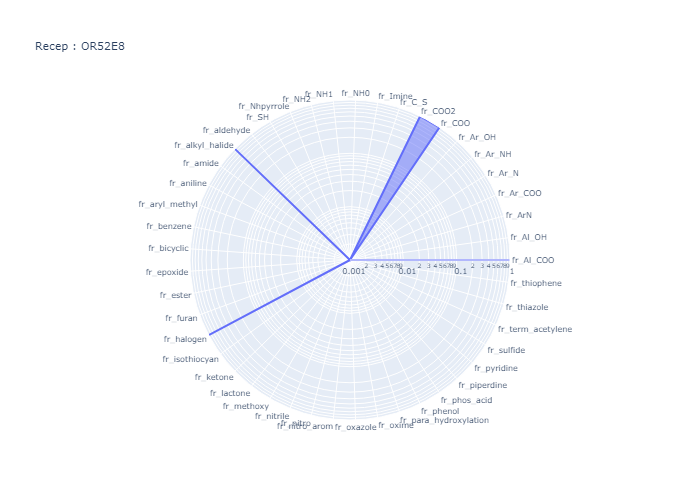


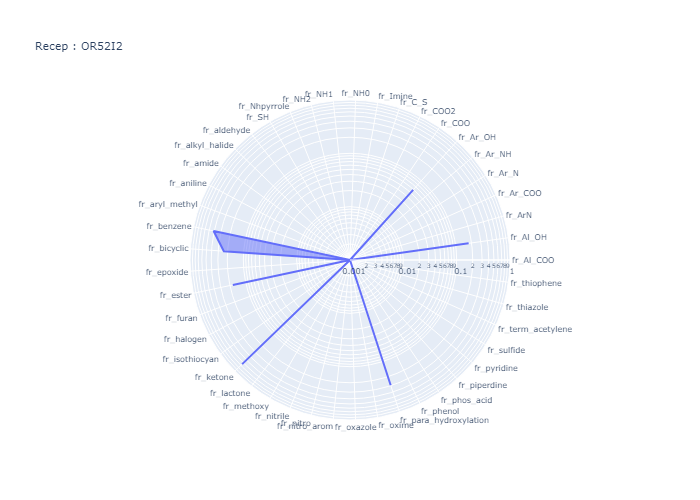

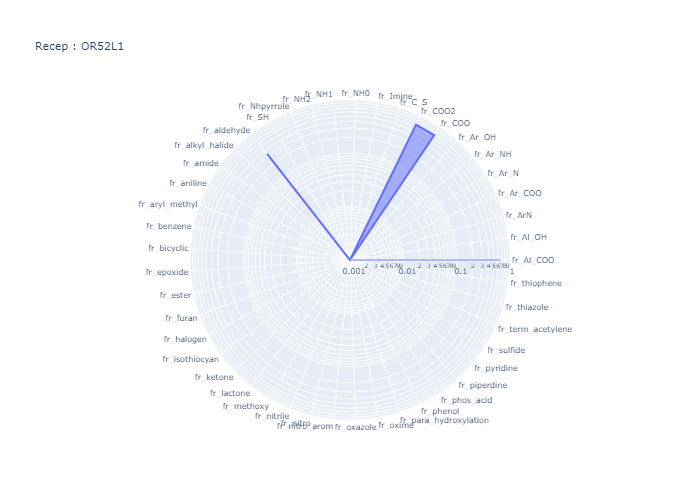


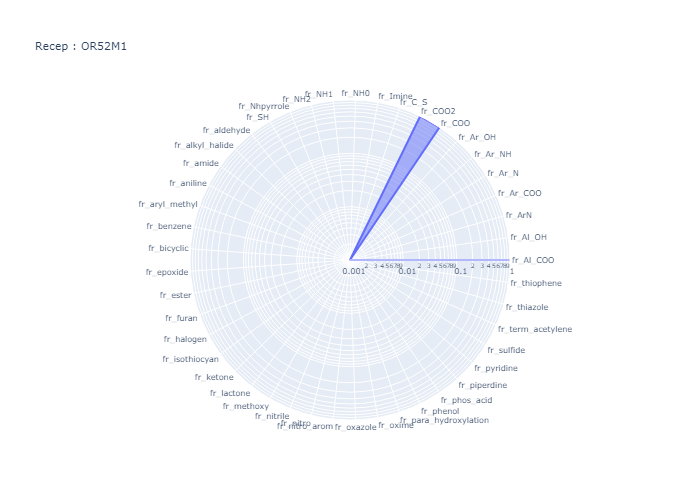

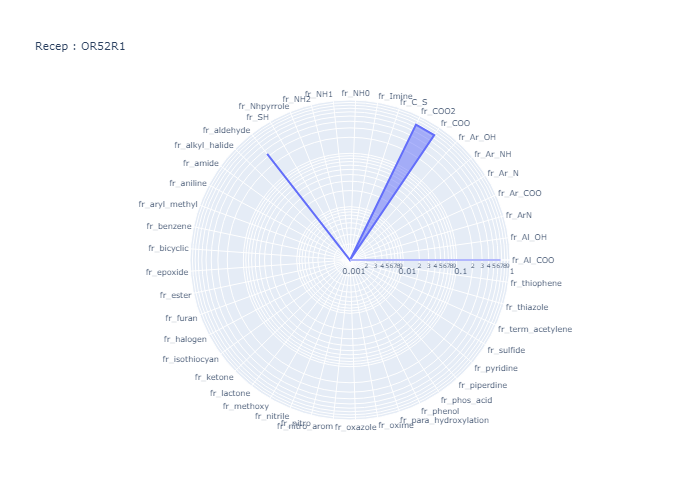

Supplement: Supplementary file 2 — Supplementary Information 2. [file 41598_2022_23176_MOESM2_ESM.docx]
